# Ketone body oxidation increases cardiac endothelial cell proliferation

**DOI:** 10.15252/emmm.202114753

**Published:** 2022-02-18

**Authors:** Eva‐Maria Weis, Patrycja Puchalska, Alisa B Nelson, Jacqueline Taylor, Iris Moll, Sana S Hasan, Matthias Dewenter, Marco Hagenmüller, Thomas Fleming, Gernot Poschet, Agnes Hotz‐Wagenblatt, Johannes Backs, Peter A Crawford, Andreas Fischer

**Affiliations:** ^1^ Division Vascular Signaling and Cancer German Cancer Research Center (DKFZ) Heidelberg Germany; ^2^ Division of Molecular Medicine Department of Medicine University of Minnesota Minneapolis MN USA; ^3^ Bioinformatics and Computational Biology Program University of Minnesota Minneapolis MN USA; ^4^ Institute of Experimental Cardiology Heidelberg University Hospital Heidelberg Germany; ^5^ German Center for Cardiovascular Research (partner site Heidelberg/Mannheim) Heidelberg Germany; ^6^ Department of Internal Medicine I and Clinical Chemistry University Hospital Heidelberg Heidelberg Germany; ^7^ Centre for Organismal Studies (COS) Heidelberg University Heidelberg Germany; ^8^ Core Facility Omics IT and Data Management (ODCF) German Cancer Research Center (DKFZ) Heidelberg Germany; ^9^ Department of Biochemistry, Molecular Biology, and Biophysics University of Minnesota Minneapolis MN USA; ^10^ Institute for Clinical Chemistry University Medical Center Göttingen Göttingen Germany; ^11^ European Center for Angioscience (ECAS) Medical Faculty Mannheim University of Heidelberg Mannheim Germany

**Keywords:** angiogenesis, endothelial cell, heart, ketogenic diet, ketone bodies, Cardiovascular System, Metabolism, Vascular Biology & Angiogenesis

## Abstract

Blood vessel formation is dependent on metabolic adaption in endothelial cells. Glucose and fatty acids are essential substrates for ATP and biomass production; however, the metabolism of other substrates remains poorly understood. Ketone bodies are important nutrients for cardiomyocytes during starvation or consumption of carbohydrate‐restrictive diets. This raises the question whether cardiac endothelial cells would not only transport ketone bodies but also consume some of these to achieve their metabolic needs. Here, we report that cardiac endothelial cells are able to oxidize ketone bodies and that this enhances cell proliferation, migration, and vessel sprouting. Mechanistically, this requires succinyl‐CoA:3‐oxoacid‐CoA transferase, a key enzyme of ketone body oxidation. Targeted metabolite profiling revealed that carbon from ketone bodies got incorporated into tricarboxylic acid cycle intermediates as well as other metabolites fueling biomass production. Elevation of ketone body levels by a high‐fat, low‐carbohydrate ketogenic diet transiently increased endothelial cell proliferation in mouse hearts. Notably, in a mouse model of heart hypertrophy, ketogenic diet prevented blood vessel rarefication. This suggests a potential beneficial role of dietary intervention in heart diseases.

## Graphical abstract

Vascular endothelial cells are shown to be capable of taking up and oxidizing ketone bodies, which enhances cell proliferation, migration and vessel sprouting.

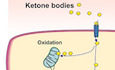

**The paper explained**

**Problem**

Ketone bodies are produced by the liver, for example, during starvation and are an important source for energy production in heart and brain. Endothelial cells are located at the interface between blood and parenchymal cells and thereby are intimately involved in the transport of ketone bodies. Whether vascular endothelial cells are able to use ketone bodies was not known, however, it was shown before that lymphatic endothelial cells can break down ketone bodies and that this stimulates lymphangiogenesis.

**Results**

Here, we describe that vascular endothelial cells can take up ketone bodies and can use these to generate biomass. Vascular endothelial cells from different organs express succinyl‐CoA:3‐oxoacid‐CoA transferase (SCOT), the key enzyme for ketone body oxidation which eventually results in acetyl‐CoA production. Metabolic profiling of cultured endothelial cells supplemented with labeled ketone bodies (acetoacetate and β‐hydroxybutyrate) showed that its breakdown products were incorporated into TCA cycle intermediates, amino acids and lipids indicating that ketone bodies can be used as a source for biomass production in endothelial cells. Moreover, under starvation, ketone bodies increased ATP production in endothelial cells. Under cell culture conditions, ketone bodies promoted endothelial cell proliferation and angiogenesis. In mice, ketogenic diet only transiently increased endothelial cells proliferation in the heart but not other organs. Although ketogenic diet did not cause an increase in blood vessel density in the heart, it prevented vessel rarefication in a model of heart hypertrophy.

**Impact**

This study shows that ketone bodies can contribute to biomass and ATP production in endothelial cells, two processes that are underlying cell proliferation and angiogenesis. Whether this can be utilized to prevent vessel rarefication, which for instance is typically observed during ageing, is unknown, but should be considered for being explored in future studies.

## Introduction

Expansion of the blood vessel network is prerequisite not only for development but also in the adult organism during tissue growth, regeneration, or wound healing. While insufficient blood vessel formation prevents regeneration of ischemic tissue, excessive and deregulated angiogenesis is implicated in several ocular diseases and cancer (Potente *et al*, 2011). In the last years, it became clear that angiogenesis is not only coordinated by growth factors and signaling transduction pathways but is also dependent on adaption in cellular metabolism (Eelen *et al*, 2018). For example, sprouting of new vessel branches is dependent on increased glycolysis (De Bock *et al*, 2013; Schoors *et al*, 2014), whereas fatty acid beta‐oxidation (FAO) is key for biomass generation and endothelial cell (EC) proliferation (Schoors *et al*, 2015). Also, lymphatic EC proliferation, migration, and sprouting rely on FAO (Wong *et al*, 2017). A recent study reported that lymphatic ECs can not only oxidize fatty acids but also ketone bodies and that this promotes formation of new lymphatic vessels in mice (Garcia‐Caballero *et al*, 2019; Puchalska & Crawford, 2019). However, it has not yet been reported whether also vascular ECs can take up and oxidize ketone bodies and if this would affect their angiogenic properties.

Ketone body metabolism enables mammals to endure periods of carbohydrate restriction. During starvation, intensive endurance sports or consumption of carbohydrate‐restrictive diets, glycogen stores get depleted and insulin levels decline. This stimulates lipolysis of triacylglycerides in adipocytes and release of free fatty acids and glycerol into the bloodstream. Free fatty acids are taken up by non‐adipocytes and broken down into acetyl‐CoA moieties via FAO to fuel the tricarboxylic acid (TCA) cycle, thereby contributing to energy production (Puchalska & Crawford, 2017). In the liver, however, TCA cycle intermediates are shunt into gluconeogenesis in situations of glucose deprivation. This results in a surplus of acetyl‐CoA in hepatic mitochondria whenever FAO‐derived acetyl‐CoA exceeds flux through the TCA cycle. Under these circumstances, acetyl‐CoA molecules are enzymatically converted into the ketone bodies acetoacetate and β‐hydroxybutyrate (Puchalska & Crawford, 2021). In the healthy, fed state serum ketone body (acetoacetate and β‐hydroxybutyrate) concentrations in humans are low (50–200 µM). Physiological ketosis, for example, during starvation increases serum ketone body concentrations to around 1–2 mM, whereas pathological ketosis, as observed in untreated type 1 diabetes patients, is characterized by very high serum ketone body concentrations up to 20 mM (Robinson & Williamson, 1980).

Newly synthesized ketone bodies are released from hepatocytes into the bloodstream and function as energy‐rich alternative metabolic fuel in particular for heart and skeletal muscle. The rate of ketone body oxidation in extrahepatic organs increases proportionally to serum ketone body concentrations. In myocytes, D‐β‐hydroxybutyrate dehydrogenase (BDH1) catalyzes the oxidation of β‐hydroxybutyrate, the predominant ketone body molecule in blood, to acetoacetate. Acetoacetate is then transferred onto CoA through the activity of succinyl‐CoA:3‐oxoacid‐CoA transferase (SCOT, encoded by *3‐oxoacid CoA‐transferase 1* (*Oxct1*)). Acetoacetyl‐CoA can then catalyzed by mitochondrial thiolase into acetyl‐CoA fueling the TCA cycle for energy production (Cotter *et al*, 2013).

Notably, ketone bodies and other circulating nutrients need to pass the endothelial barrier, the inner surface of blood vessels, before being taken up by tissue cells. Whereas the sinusoidal endothelium in the liver allows free diffusion, the continuous endothelium in, for example, muscle, lung, or brain requires transcellular transport (Augustin & Koh, 2017). This raises the question whether ECs function to transport ketone bodies from blood toward tissue cells only or whether they are also able to use ketone bodies themselves. Based upon the outstanding importance of cellular metabolism for controlling angiogenesis (Eelen *et al*, 2018), this study aimed at analyzing ketone body metabolism in vascular ECs. The study focused on cardiac ECs, as the heart is one of the main consumers of ketone bodies during physiological ketosis.

## Results

### Cardiac ECs oxidize ketone bodies via the TCA cycle

**Figure 1:**
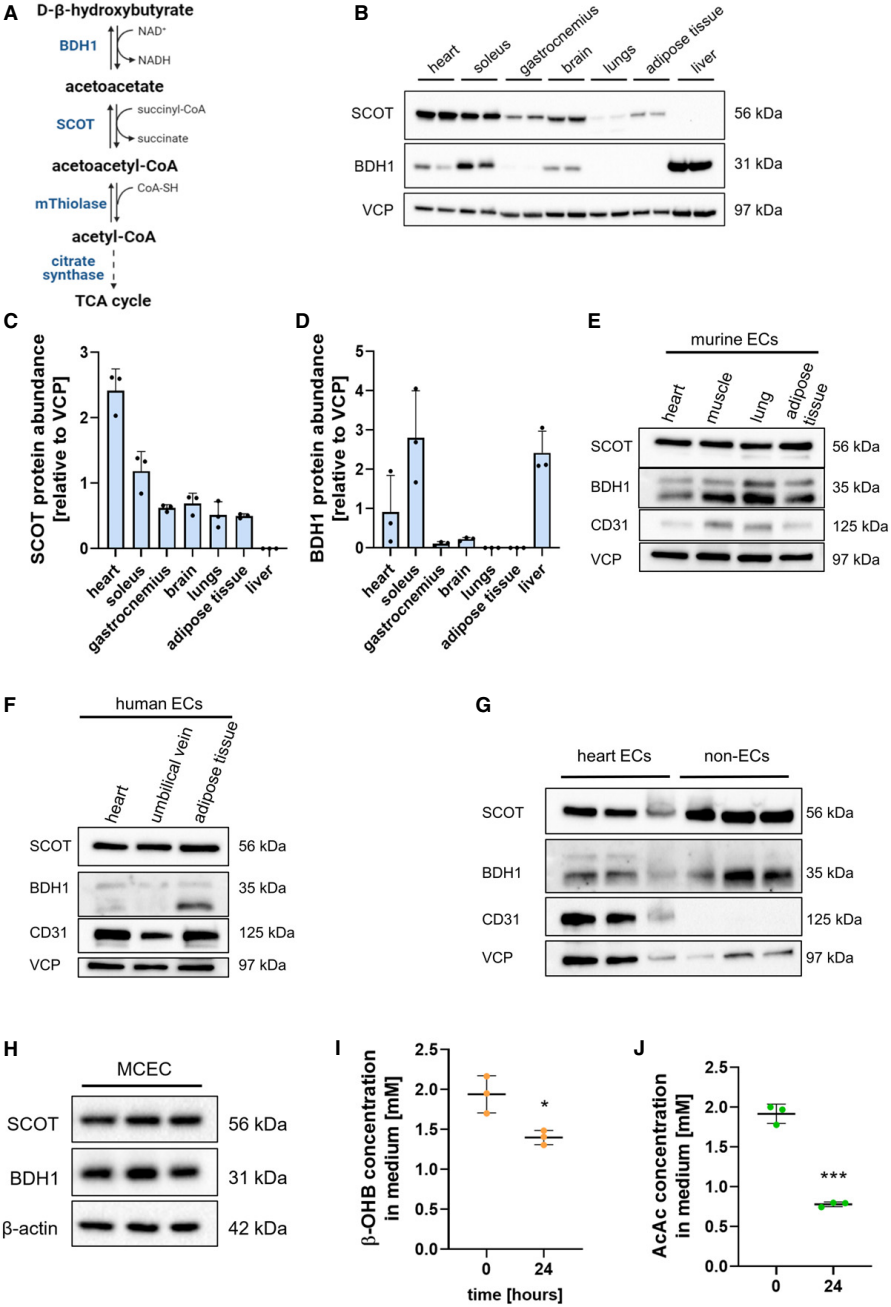
Key enzymes of ketone body oxidation are expressed in cardiac endothelial cells AOxidation of ketone bodies requires the enzymes SCOT and BDH1 and yields acetyl‐CoA which enters the tricarboxylic acid (TCA) cycle.BImmunoblot of SCOT, BDH1, and the loading control VCP across various organs obtained from adult C57Bl/6J mice.C, DQuantification of SCOT and BDH1 expression levels relative to VCP in various murine organs.EImmunoblot of SCOT, BDH1, CD31, and the loading control VCP in murine endothelial cells isolated from heart, muscle, lung, and adipose tissue.FImmunoblot of SCOT, BDH1, CD31, and the loading control VCP in human cardiac ECs, HUVECs, and adipose tissue ECs.GImmunoblot of SCOT, BDH1, CD31, and VCP in cardiac endothelial cells (EC) and non‐endothelial cell fraction (non‐EC) isolated from adult C57Bl/6J mice.HImmunoblot of SCOT, BDH1, and β‐actin in immortalized murine cardiac endothelial cells (MCEC).I, JKetone body concentration in cell culture medium of MCEC treated with 2 mM D‐β‐hydroxybutyrate (β‐OHB) or 2 mM acetoacetate (AcAc) after 24 h. Data are presented as mean ± SD. *n* = 3; *P < 0.05; ***P < 0.001 unpaired Student’s *t*‐test. Source data are available online for this figure.

Oxidation of ketone bodies (Fig 1A) requires the key enzyme SCOT. Heart, skeletal muscle, and brain are the main consumers of ketone bodies (Puchalska & Crawford, 2017) and SCOT protein was abundantly present in tissue lysates of these organs obtained from adult C57Bl/6J mice, as expected (Fig 1B and C). We also detected weak SCOT expression in adipose tissue, whereas it was not detectable in lung and liver. Expression of the oxidoreductase BDH1, which reversibly converts D‐β‐hydroxybutyrate into acetoacetate, showed a variable organ expression pattern with highest expression in the liver, the organ that is responsible for ketone body production (Fig 1B and D). In the heart, cardiomyocytes oxidize ketone bodies, but whether other cell types in the heart also oxidize ketones is unknown (Schugar *et al*, 2014; Horton *et al*, 2019). Indeed, we detected high SCOT and BDH1 expression levels in freshly isolated murine cardiomyocytes. Surprisingly however, we also observed expression of both proteins in the non‐cardiomyocyte fraction, in particular in CD31^+^ vascular ECs (Fig 1G).

Endothelial SCOT and BDH1 protein expression was not only restricted to cardiac ECs but also detectable in ECs derived from skeletal muscle, lung, and adipose tissue of adult mice (Fig 1E). Also, ECs derived from human umbilical cord vein (HUVEC), heart, and adipose tissue expressed SCOT and BDH1 protein (Fig 1F). Analysis of single‐cell RNA sequencing data from isolated ECs of multiple murine organs (Kalucka *et al*, 2020) revealed that Oxct1 mRNA (encoding SCOT protein) gets transcribed in ECs of all organs analyzed. In the heart, Oxct1 mRNA expression was uniformly distributed between arterial, capillary, and venous ECs (Appendix Fig S1). To test for potential upstream regulators of Oxct1 and Bdh1, immortalized murine cardiac ECs (MCEC) as well as primary human cardiac microvascular ECs and HUVECs were treated with inflammatory cytokines (IL1β, IL6, TNFα), pro‐angiogenic VEGF‐A, cultured under normoxic versus hypoxic conditions and starved versus FCS‐treated conditions. However, these experiments did not lead to the identification of an upstream regulatory factor (Appendix Fig S2).

To test whether expression of SCOT and BDH1 would allow vascular ECs to metabolize ketone bodies, we first analyzed whether MCEC are able to take up ketone bodies from the culture medium. These cells express the ketolytic enzyme SCOT as well as BDH1 (Fig 1H). When MCEC were cultured in the presence of 2 mM acetoacetate or 2 mM D‐β‐hydroxybutyrate, there was a marked decrease of ketone body concentration 24 h later in the culture medium (Fig 1I and J). This indicates that MCEC take up ketone bodies.

**Figure 2:**
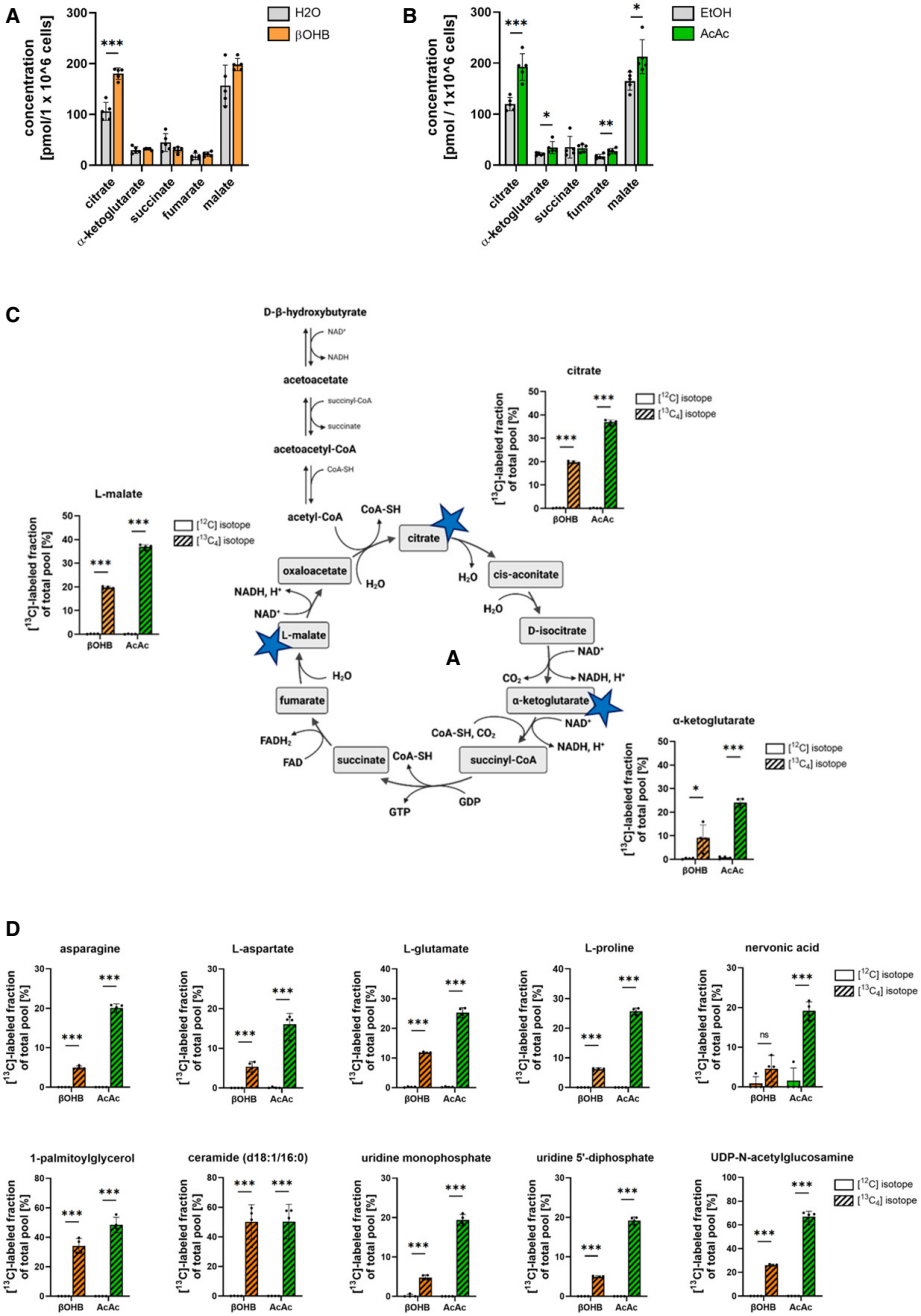
Ketone body treatment increases TCA cycle intermediates in MCEC A, BMurine cardiac endothelial cells (MCEC) were treated with 2 mM D‐β‐hydroxybutyrate (β‐OHB) or 2 mM acetoacetate (AcAc) for 24 h. Quantification of concentrations of TCA cycle intermediates compared to control treatment.CScheme of ketone body oxidation and the tricarboxylic acid (TCA) cycle. MCEC were incubated with 1 mM ^13^C_4_‐β‐hydroxybutyrate or 1 mM ^13^C_4_‐acetoacetate for 24 h or the same concentration of unlabeled ketone bodies ([^12^C] isotope). The [^13^C]‐labeled fractions of the TCA cycle intermediates citrate, α‐ketoglutarate and malate (marked with blue star) were quantified with an untargeted metabolomics approach indicating that ^13^C carbon from ketone bodies was incorporated into TCA cycle intermediates.DQuantifications of [^13^C]‐labeled fractions of amino acids (asparagine, l‐aspartate, l‐glutamate, l‐proline), putative lipid species (nervonic acid, 1‐palmitoylglycerol, ceramide (d18:1/16:0)) and uridine diphosphate (UDP) species (uridine monophosphate, uridine 5'‐diphosphate, UDP‐N‐acetylglucosamine) in MCEC treated with 1 mM ^13^C_4_‐β‐hydroxybutyrate or 1 mM ^13^C_4_‐acetoacetate for 24 h or the same concentration of unlabeled ketone bodies ([^12^C] isotope). Data are presented as mean ± SD. *n* ≥ 4; ns, not significant; *P < 0.05; **P < 0.01; ***P < 0.001 unpaired Student’s *t*‐test.

Interestingly, targeted metabolite profiling also showed that treatment of MCEC with both acetoacetate or D‐β‐hydroxybutyrate increased the absolute concentration of several tricarboxylic acid (TCA) cycle intermediates such as citrate (Fig 2A and B). To investigate the ability of MCEC to metabolize ketone bodies, isotope tracing untargeted metabolomics using ketone bodies was performed. MCEC that were treated with uniformly labeled [U‐^13^C_4_] acetoacetate or [U‐^13^C_4_] D‐β‐hydroxybutyrate for 24 h had incorporated the ^13^C isotope into numerous metabolites indicating that cardiac ECs do not only take up ketone bodies but also metabolize them. Most importantly, ^13^C incorporation was observed in TCA cycle intermediates, amino acids and putative purines and lipids (Fig 2C and D). The ^13^C incorporation rate was almost always higher in the presence of acetoacetate compared to D‐β‐hydroxybutyrate. This is most likely due to the fact that D‐β‐hydroxybutyrate oxidation requires an additional enzymatic oxidation step into acetoacetate by BDH1 (Fig 1A). Together, these data indicate that cardiac ECs take up ketone bodies to feed the TCA cycle and to generate biomass.

### Ketone bodies are a source for mitochondrial respiration in cultured ECs

**Figure EV1:**
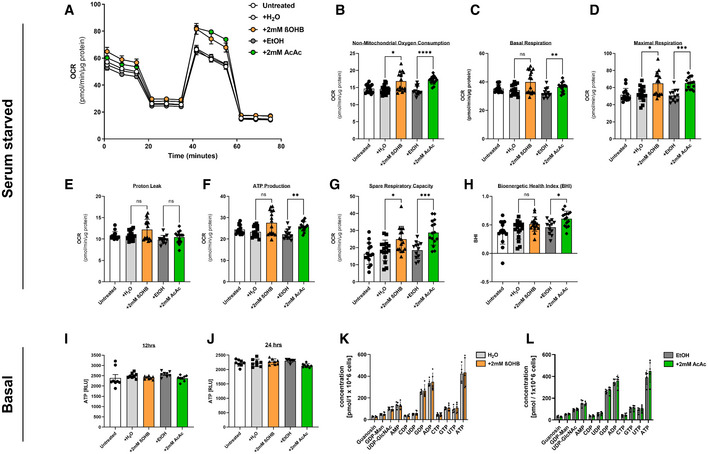
Effects of ketone bodies on mitochondrial respiration Mitochondrial function of MCECs stimulated with ketone bodies was characterized using the Seahorse Bioanalyzer by measuring the rate of oxygen consumption (OCR) following sequential additions of oligomycin, FCCP and antimycin/rotenone (A) to the cells to determine non‐mitochondrial oxygen consumption (B), basal respiration (C), maximal respiration (D), proton leakage (E), ATP production (F), spare respiratory capacity (G), and the bioenergetic health index (H), respectively.
A–HMCECs were starved for 1 h before addition of 2 mM R‐β‐hydroxybutyrate (βOHB) compared to H_2_O and acetoacetate (AcAc) compared to ethanol for 24 h.I–LMCECs were cultured in basal medium before addition of ketone bodies (2 mM) and solvent control. (I, J) Cellular ATP contents after 12 and 24 h. (K, L) Targeted metabolomics using UPLC to determine cellular nucleotides upon treatment with ketone bodies for 24 h. Data are presented as mean ± SD. One‐way ANOVA using nonparametric (Kruskal–Wallis) test. ****P < 0.0001, ***P < 0.001, **P < 0.01, *P < 0.05, NS > 0.05.

To test whether ketone bodies can also be oxidized by cultured ECs to feed to mitochondrial electron transfer chain and ATP production, we employed Seahorse technology (XF Cell Mito Stress Kit). This revealed that the addition of ketone bodies to the culture medium of serum‐starved MCEC allows for an increase in maximal mitochondrial respiration and ATP production (Fig EV1A–H).

When MCEC were cultured in full medium under basal conditions, further addition of ketone bodies did not increase the amount of ATP (Fig EV1I and J). Also, the amounts of other nucleotides were not changed by addition of ketone bodies (Fig EV1K and L). As ATP cannot be sufficiently stored by cells, this finding was not surprising. In summary, the experiments shown so far revealed that ECs can uptake and oxidize ketone bodies to either produce ATP (e.g., during starvation) or biomass.

### Ketone bodies promote angiogenic capacity of cultured cardiac ECs

**Figure 3:**
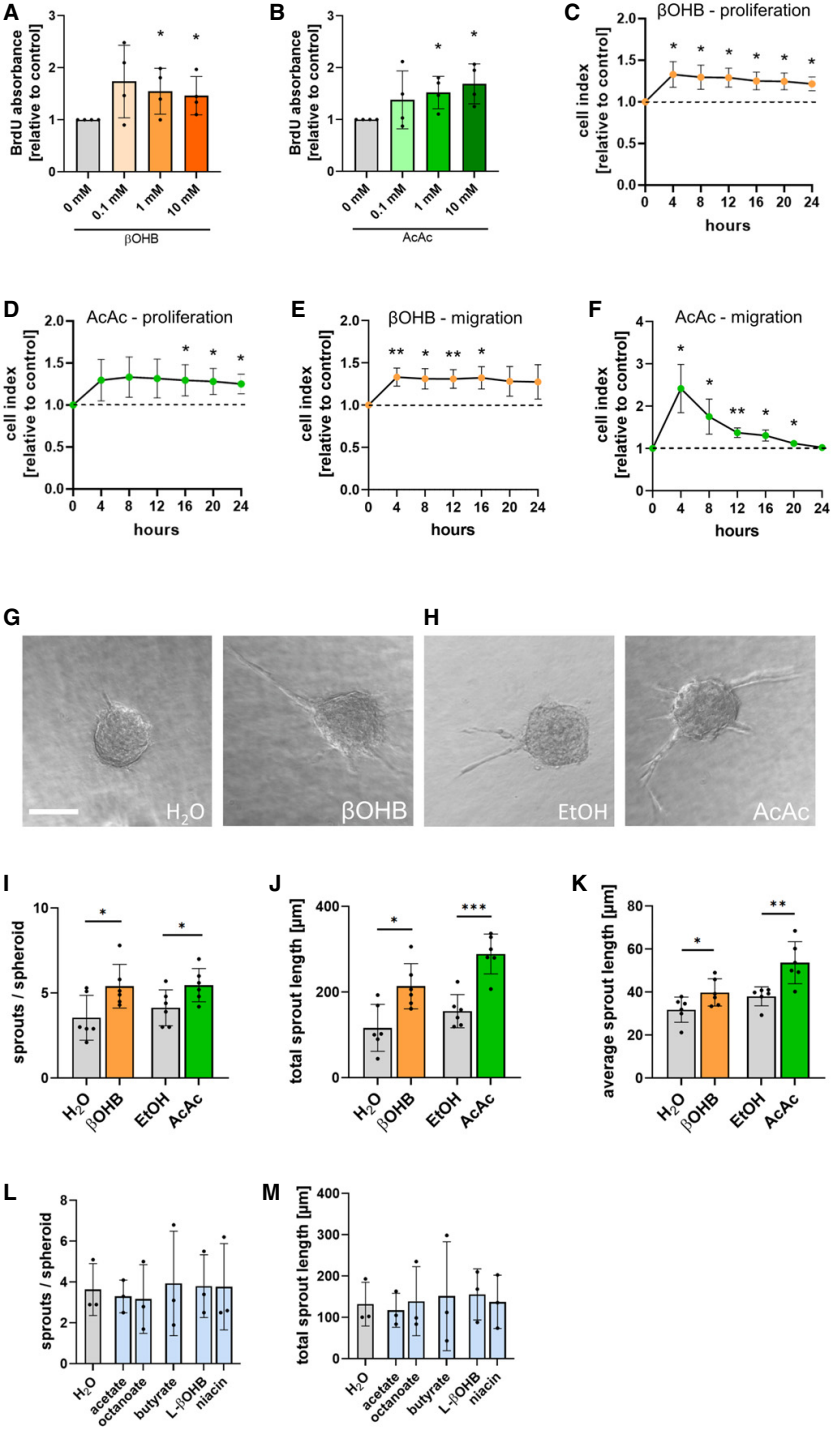
Ketone body treatment promotes endothelial cell proliferation, migration, and sprouting capacity A, BMurine cardiac endothelial cells (MCEC) were treated with different concentrations of D‐β‐hydroxybutyrate (βOHB) or acetoacetate (AcAc) for 24 h. Relative BrdU absorbance was quantified compared to control treatment (H_2_O for βOHB and EtOH for AcAc).C, DMCEC were treated with 10 mM D‐β‐hydroxybutyrate (βOHB) or 10 mM acetoacetate (AcAc) for 24 h. Electrical impedance was measured compared to control treatment (dashed line; H_2_O for βOHB and EtOH for AcAc).E, FMCEC were seeded onto Boyden chambers and treated with (E) 10 mM D‐β‐hydroxybutyrate (βOHB) or (F) 10 mM acetoacetate (AcAc) for 24 h. Electrical impedance was measured in the lower chamber compared to control treatment (dashed line; H_2_O for βOHB and EtOH for AcAc).G, HRepresentative images of MCEC spheroids treated with H_2_O, 30 mM R‐β‐hydroxybutyrate (βOHB), ethanol (EtOH) or 30 mM acetoacetate (AcAc) for 72 h; scale bar: 50 µm.I–MAngiogenic capacity of MCEC in response to ketone body treatment was analyzed using a spheroid‐based sprouting assay. Spheroids were treated with media containing 30 mM D‐β‐hydroxybutyrate (βOHB), 30 mM acetoacetate (AcAc), 10 mM acetate, 1 mM octanoate, 1 mM butyrate, 30 mM L‐β‐hydroxybutyrate (L‐ βOHB), 1 mM niacin or the respective controls (final concentration of reagents is diluted to approximately 10%) and analyzed after 48 h. The (I, L) average number of sprouts per spheroid, the (J, M) accumulated total sprout length and (K) average sprout length were quantified. Data are presented as mean ± SD. *n* ≥ 3; *P < 0.05; **P < 0.01; ***P < 0.001 unpaired Student’s *t*‐test.

**Figure EV2:**
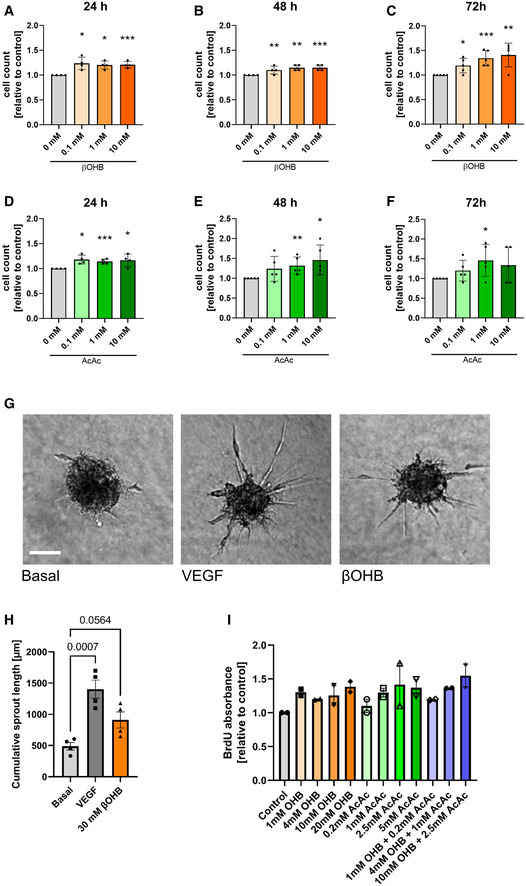
Effects of ketone body supplementation on sprouting potential A–CCell counts of MCECs treated with 0.1, 1 or 10 mM R‐β‐hydroxybutyrate (βOHB) for 24, 48, or 72 h compared to treatment with H_2_O (control).D–FCell counts of MCECs treated with 0.1, 1 or 10 mM acetoacetate (AcAc) for 24, 48, or 72 h compared to treatment with ethanol (control).GRepresentative images of HUVEC spheroids treated with H_2_O (control), recombinant VEGF‐A165, 30 mM R‐β‐hydroxybutyrate (βOHB) for 72 h; scale bar: 50 μm.HQuantification of the accumulated total sprout length per spheroid. *n* = 4.IRelative absorbance of BrdU incorporated into DNA of HUVEC treated with 1, 4 or 10 mM R‐β‐hydroxybutyrate (βOHB), 0.1, 1 or 10 mM acetoacetate (AcAc) or a combination of both for 24 h. Data are presented as mean ± SD. *n* ≥ 2. One‐way ANOVA; *P < 0.05; **P < 0.01; ***P < 0.001.

As biomass production is key for EC growth and cell division (Eelen *et al*, 2018), we investigated whether the presence of ketone bodies would allow cardiac ECs to proliferate faster. In the presence of D‐β‐hydroxybutyrate or acetoacetate in the culture media, MCEC showed higher levels of BrdU incorporation as a marker of DNA synthesis (Fig 3A and B). When cells were cultured on gold microelectrodes, elevated electrical impedance was observed, indicating increased cell proliferation rates in response to ketone body treatment (Fig 3C and D). Consistently, treatment with ketone bodies (0.1–10 mM) increased the number of MCEC upon 24, 48, and 72 h of culture compared to a solvent control (Fig EV2A–F). In addition, MCEC exposed to D‐β‐hydroxybutyrate or acetoacetate migrated faster in a Boyden chamber assay when compared to control treatment (Fig 3E and F).

EC proliferation and migration are prerequisites for angiogenesis, the formation of new blood vessels from pre‐existing ones (Potente *et al*, 2011). In a three‐dimensional spheroid‐based angiogenesis assay in collagen, treatment with D‐β‐hydroxybutyrate or acetoacetate resulted in increased capillary sprout formation (Fig 3G and H), as quantified by the numbers of sprouts and the cumulative and average sprout length (Fig 3I–K). Similar results were also obtained with human umbilical vein ECs (HUVEC), indicating that this effect is not restricted to cardiac‐specific ECs (Fig EV2G–I).

To test whether this effect is specific for D‐β‐hydroxybutyrate and acetoacetate, MCEC spheroids were cultured in the presence of other substrates which can feed into the TCA cycle such as acetate, the short‐chain fatty acid butyrate, or the medium‐chain fatty acid octanoate. However, these did not promote the angiogenic potential (Fig 3L and M). To rule out that the observed pro‐angiogenic effect of ketone bodies on cardiac ECs is based on oxidation‐independent mechanisms, we performed a series of experiments. First, MCEC were treated with ketone body enantiomer L‐β‐hydroxybutyrate, that is not a substrate of BDH1 and can therefore not be oxidized. Notably, only D‐β‐hydroxybutyrate (Fig 3I–K), but not L‐β‐hydroxybutyrate (Fig 3L–M) promoted tube formation capacity of MCEC. Secondly, we addressed the possibility whether D‐β‐hydroxybutyrate would exert its functions by inhibiting class I histone deacetylases (HDAC) as this has been shown by treating mice with high doses (Shimazu *et al*, 2013). In ECs, there are contradictory reports about the efficiency of D‐β‐hydroxybutyrate acting as a HDAC inhibitor (Chriett *et al*, 2019; Li *et al*, 2021). To rule out the possibility that increased angiogenic potential of MCEC would merely be the consequence of HDAC inhibition, MCEC were treated with the well‐established HDAC inhibitor butyrate (Shimazu *et al*, 2013). In contrast to D‐β‐hydroxybutyrate, butyrate treatment had no effect on the tube formation capacity, indicating that the observed effects are unlikely to be mediated through histone acetylation (Fig 3L and M). Lastly, D‐β‐hydroxybutyrate has been shown to act as a ligand for hydroxycarboxylic acid receptor‐2 (HCA2, also known as niacin receptor‐1 (NICAR1) or GPR109A) (Graff *et al*, 2016). We aimed at ruling out that the proangiogenic effect of D‐β‐hydroxybutyrate would be mediated through stimulation of HCA2. Therefore, MCEC were treated with niacin, an alternative ligand for HCA2. There were no differences in the angiogenic potential of MCEC in response to niacin when compared to control treatment, ruling out that niacin receptor signaling plays a substantial role in the observed phenotype (Fig 3L and M).

### SCOT is necessary for the pro‐angiogenic effects of ketone bodies

**Figure 4:**
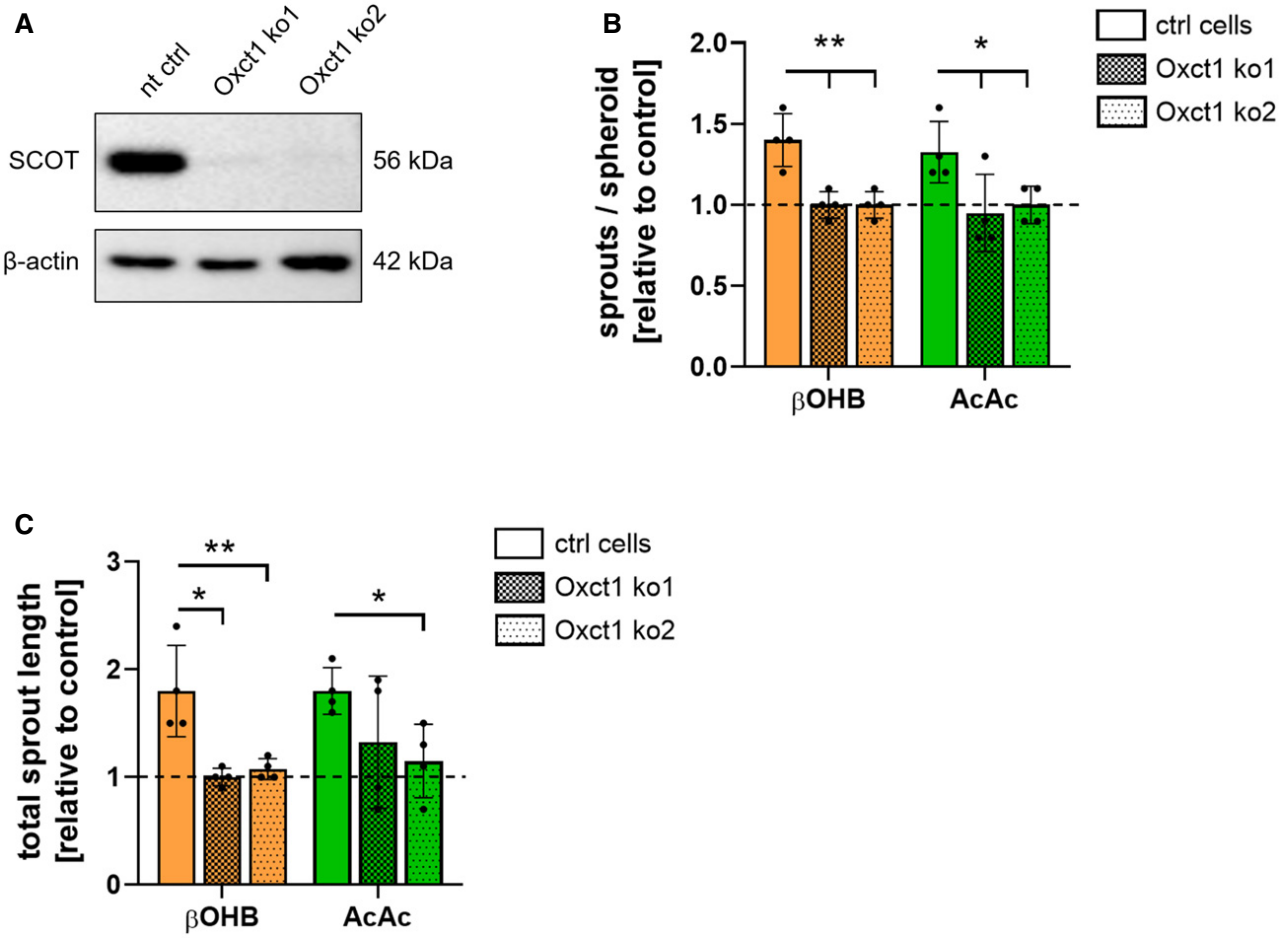
Ketone body treatment does not increase sprouting capacity in SCOT‐deficient cardiac endothelial cells ASCOT‐deficient murine cardiac endothelial cells were generated using the CRISPR/Cas9 technology by targeting the SCOT‐encoding gene *Oxct1*. A non‐targeting control construct was used to generate the non‐targeting control cells (nt ctrl). Immunoblot of SCOT and β‐actin in non‐targeting control cells (nt ctrl) and Oxct1 knockout cells (Oxct1 ko).B, CAngiogenic capacity of Oxct1 knockout cells in response to ketone body treatment was analyzed using a spheroid‐based sprouting assay. Spheroids were treated with media containing 30 mM D‐β‐hydroxybutyrate (βOHB), 30 mM acetoacetate (AcAc) or the respective controls (final concentration of reagents is diluted to approximately 10%) for 48 h. The average number of sprouts per spheroid and the accumulated sprout length was quantified. Data are presented as mean ± SD. *n* = 4; *P < 0.05; **P < 0.01; unpaired Student’s *t*‐test. Source data are available online for this figure.

The data presented so far indicate that oxidation of ketone bodies is subsequently promoting cell proliferation and tube formation in ECs. To further prove this, we generated SCOT‐deficient MCEC lines using CRISPR/Cas9 technology. This resulted in a complete loss of SCOT protein expression in MCEC (Fig 4A), which did not interfere with cell viability. However, SCOT‐deficient MCEC did not show an increased angiogenic potential in response to ketone body treatment in a spheroid‐based sprouting assay compared to control cells transfected with a non‐targeting plasmid construct (Fig 4B and C). In summary, the data reported thus far indicate that the oxidation of β‐hydroxybutyrate and acetoacetate is both mandatory and responsible for the increased proliferation and migration rates as well as the higher sprouting ability of ECs in response to elevated ketone body concentrations in the supernatant.

### Ketogenic diet alters gene expression of cardiac ECs in mice

**Figure 5:**
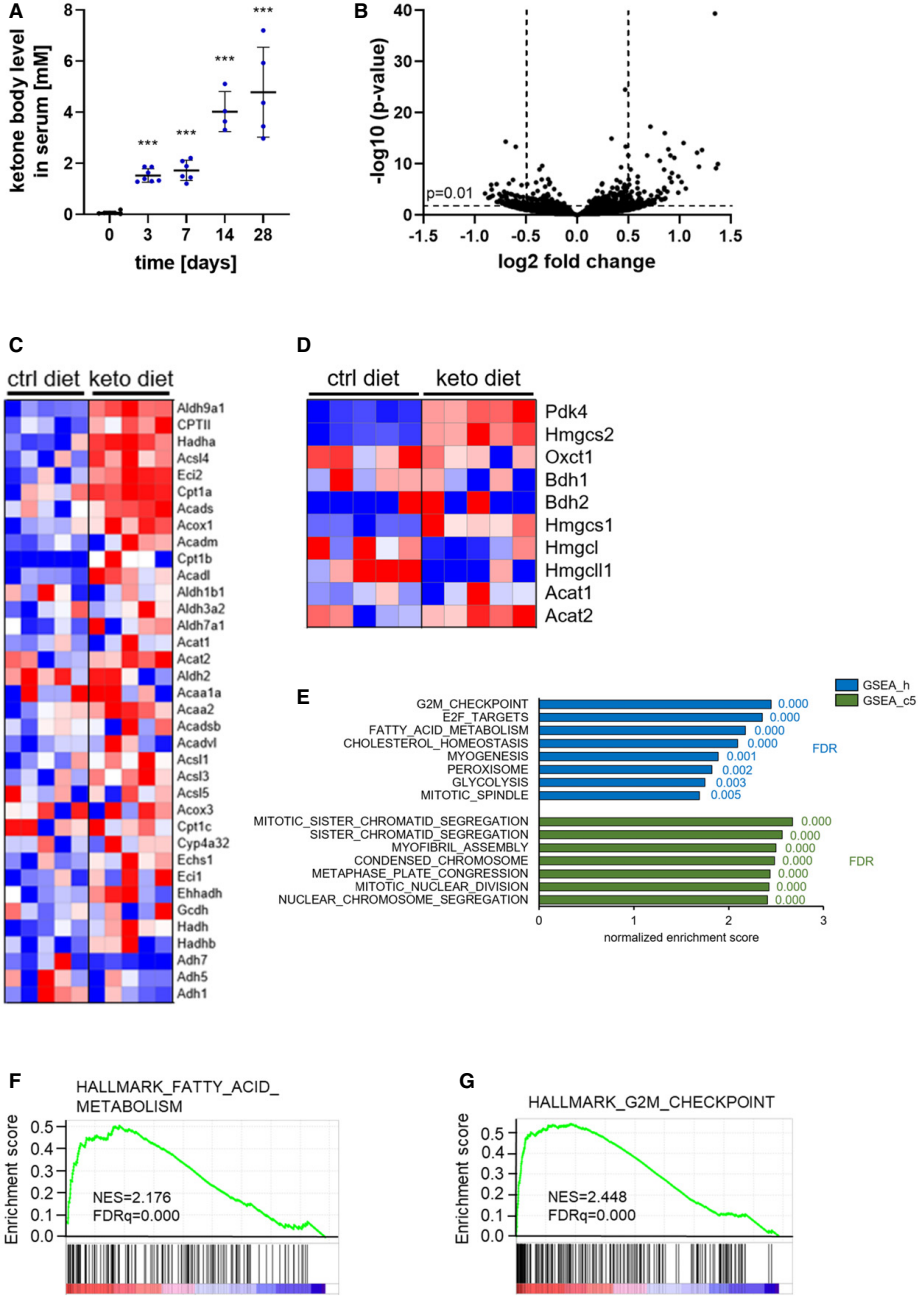
Ketogenic diet alters gene expression in cardiac endothelial cells towards a more proliferative signature AKetone body concentrations were measured in serum of adult C57Bl/6J mice that were fed a ketogenic diet for the indicated time periods.BRNA sequencing was performed on isolated cardiac endothelial cells of male C57Bl/6J mice that were fed a ketogenic diet or control diet for 2 weeks. Volcano plot showing differentially expressed genes as genes with a log2 fold change ≤ −0.5 or ≥ 0.5 (vertical dashed lines) and *P* ≤ 0.01 (horizontal dashed line).C, DHeatmaps showing expression levels of genes involved in KEGG pathways (C) fatty acid degradation and (D) ketone body metabolism in cardiac endothelial cells isolated from male C57Bl/6J mice that were fed a ketogenic diet or control diet for 2 weeks.EGene set enrichment analysis of hallmark (GSEA gene set h, blue) and gene ontology (GO)‐derived (GSEA gene set c5, green) gene sets enriched in cardiac endothelial cells of C57Bl/6J mice kept on a ketogenic diet compared to littermate animals kept on control diet.F, GEnrichment plots of the hallmark gene sets fatty acid metabolism and G2M checkpoint comparing expression pattern of cardiac endothelial cells isolated from C57Bl/6J mice kept on a ketogenic diet compared to littermate animals kept on control diet. Data are presented as mean ± SD. *n* ≥ 4; ***P < 0.001; unpaired Student’s *t*‐test.

**Figure EV3:**
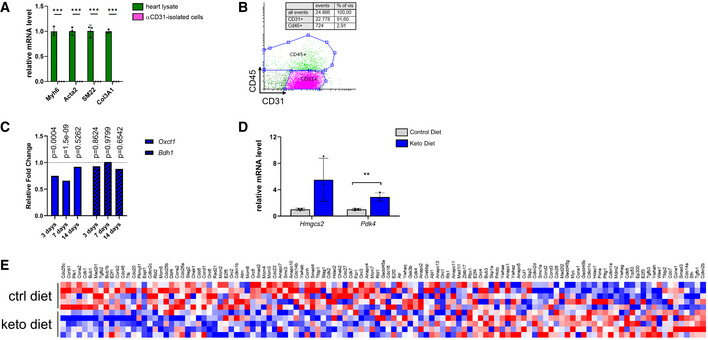
Cardiac endothelial cells isolated from mice fed a ketogenic diet Relative mRNA levels of cardiomyocyte, fibroblast, and smooth muscle cell marker genes in anti‐CD31‐isolated cells compared to levels in total heart lysates of C57Bl/6J mice.Flow cytometry analysis of anti‐CD31‐isolated cells from hearts of C57Bl/6J mice using the endothelial cell marker CD31 and the immune cell marker CD45.Relative fold changes of *Oxct1* (Scot) and *Bdh1* expression levels in mice fed a ketogenic diet relative to a control diet were obtained from RNAseq analyses.Relative mRNA levels of cardiac ECs isolated from mice fed a ketogenic or control diet confirming increased levels of pro‐proliferative genes *Hmgcs2* and *Pdk4*.Heat‐map showing expression levels of genes involved in regulation of cell cycle (KEGG). Data are presented as mean ± SD. *n* ≥ 3. Two‐tailed unpaired Student’s *t*‐test; **P < 0.01; ***P < 0.001.

We next investigated the effects of elevated serum ketone body concentrations on cardiac ECs in C57Bl/6J mice. As expected, administration of a high‐fat, very low‐carbohydrate ketogenic diet increased the ketone body concentration in blood (Fig 5A). To gain insight into the changes in gene expression caused by elevation of serum ketone body concentrations, cardiac CD31^+^ ECs were isolated (Fig EV3A and B) from mice that were fed a ketogenic diet or control diet and subjected to RNA‐sequencing. This revealed that feeding a ketogenic diet for 2 weeks changed the expression of around 150 genes in cardiac ECs when compared to cells of littermates kept on a control diet (*P*‐value ≤ 0.01, log2 fold change ≤ −0.5 or ≥ 0.5) (Fig 5B–D, Dataset EV1). Ketogenic diet led to no changes in BDH1 expression. However, expression of Oxct1 (SCOT) was significantly lower at 3 and 7 days after starting the diet, but no longer at 14 days (Fig EV3C). We performed a gene set enrichment analysis (GSEA) using the annotated hallmark gene sets from the molecular signature database (MSigDB) and observed a significant enrichment of genes involved in fatty acid metabolism (Fig 5E and F). The mitochondrial pyruvate dehydrogenase lipoamide kinase isozyme 4 (Pdk4) was significantly upregulated in cardiac ECs (Figs 5D and EV3D). Pdk4 inhibits the pyruvate dehydrogenase complex, thereby regulating the entry of glycolysis‐derived acetyl‐CoA into the TCA cycle and is also known to be upregulated in whole heart tissue lysates in response to ketogenic diet (Wentz *et al*, 2010). The upregulation of Pdk4 and Hmgcs2 expression in cardiac ECs during ketogenic diet (Fig 5D) was confirmed in a second cohort of mice (Fig EV3D).

Further GSEA showed an enrichment of genes involved in processes related to cell cycle progression and cell proliferation such as gap 2‐mitosis (G2/M) checkpoint and E2F targets (hallmark gene sets) as well as sister chromatid segregation (gene ontology‐derived, C5 gene sets) (Figs 5E and G, and EV3E). These findings indicate that elevated concentrations of ketone bodies not only induce cardiac EC proliferation *in vitro*, but might also do so in an *in vivo* setting.

### Cardiac EC proliferation in mice is transiently increased by ketogenic diet

**Figure 6:**
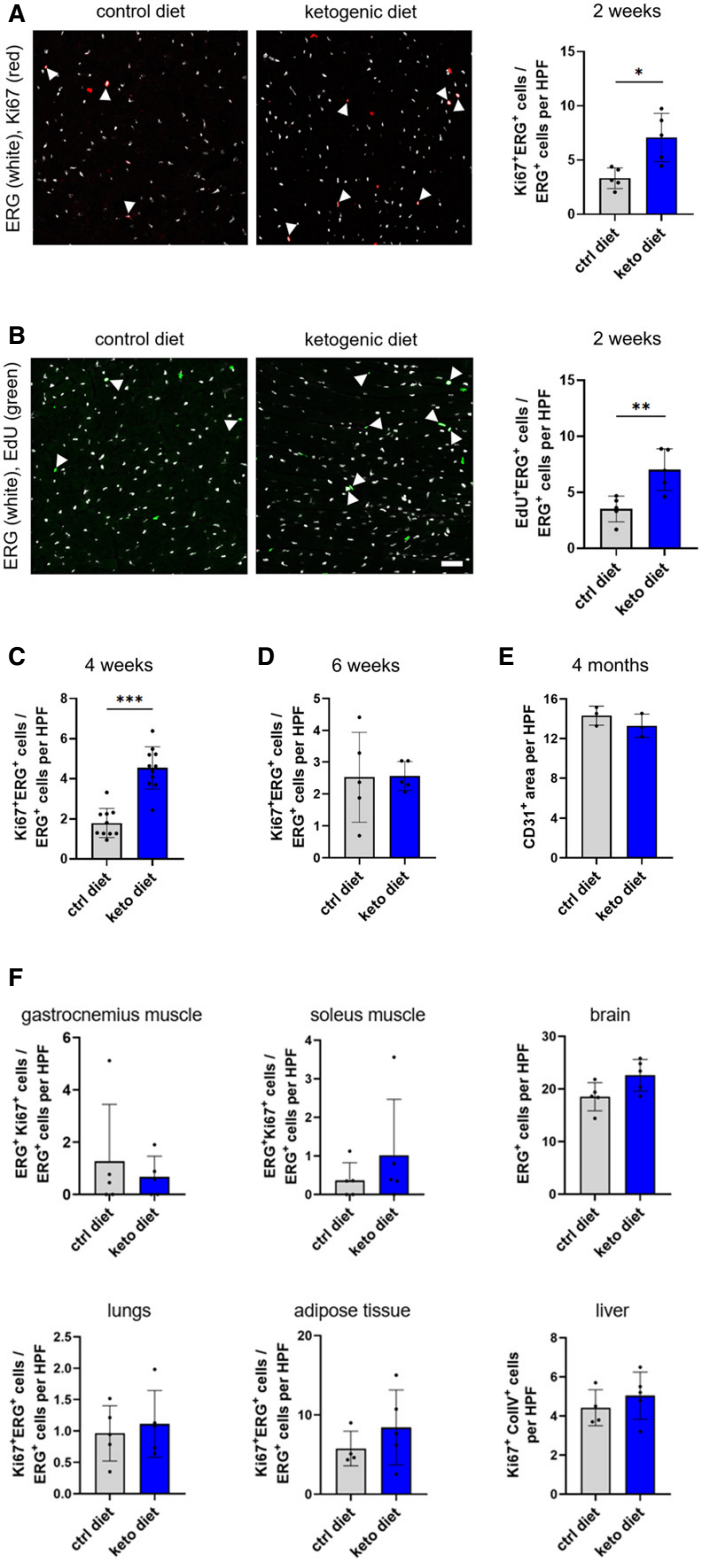
Ketogenic diet induces proliferation of cardiac endothelial cells in mice A, BRepresentative images of heart sections of animals kept on a ketogenic diet or control diet for 2 weeks stained against ERG and Ki67 or EdU. Double‐positive cells are indicated by arrowheads. Quantification is of double‐positive cells in heart sections per high power field (HPF).C, DQuantification of Ki67^+^/ERG^+^ cells in heart sections of mice kept on a ketogenic diet or control diet for 4 or 6 weeks.EQuantification of CD31^+^ area in heart sections of mice kept on a ketogenic diet or control diet for 4 months.FQuantification of Ki67^+^ endothelial cells in several organs of mice kept on a ketogenic diet or control diet for 4 weeks (gastrocnemius and soleus muscle) or 2 weeks (brain, lungs, subcutaneous adipose tissue, liver). Scale bar: 50 μm. Data are presented as mean ± SD. *n* ≥ 3; *P < 0.05; **P < 0.01; ***P < 0.001 unpaired Student’s *t*‐test.

**Figure EV4:**
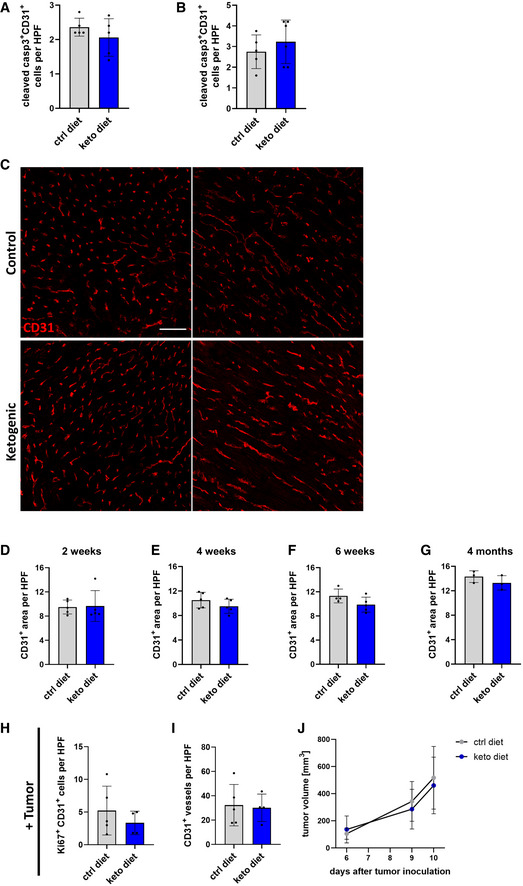
Endothelial cell apoptosis in hearts of mice receiving a ketogenic diet A, BQuantification of cleaved caspase 3^+^/CD31^+^ double‐positive cells per high power field (HPF) in heart sections of C57Bl/6J mice kept on a control diet or a ketogenic for (A) 2 weeks or (B) 4 weeks.CRepresentative images of heart sections of animals kept on the control or ketogenic diet for 4 weeks stained against CD31.D–GQuantification of CD31‐positive area per high power field (HPF) in heart sections of mice kept on control diet (ctrl diet) or ketogenic diet (keto diet) for 2 weeks / 4 weeks / 6 weeks / 4 months. Scale bar: 50 µm.HQuantification of Ki67^+^/CD31^+^ double‐positive cells per high power field (HPF) in B16F10 tumor sections of C57Bl/6J mice kept on a control diet or a ketogenic 10 days after tumor inoculation.IQuantification of CD31^+^ vessels per high power field (HPF) in B16F10 tumor sections of C57Bl/6J mice kept on a control diet or a ketogenic 10 days after tumor inoculation.JTumor volume of C57Bl/6J mice kept on a control diet or ketogenic diet. Data are presented as mean ± SD. *n* ≥ 3; statistical significance determined using unpaired Student’s *t*‐test.

Gene profiling revealed that administration of ketogenic diet alters the transcriptional landscape of cardiac ECs in a way to promote cell division. To test this hypothesis, we determined the amount of Ki‐67‐positive cardiac ECs in mice fed with control or ketogenic diet for 2 weeks. ECs were stained against erythroblast transformation‐specific‐related gene (ERG), a highly specific EC marker localized in the cell nucleus (Nikolova‐Krstevski *et al*, 2009). This allowed co‐detection of nuclear Ki‐67, a classical proliferation marker. The analysis revealed that there was a significant increase in the abundance of Ki‐67 positive ECs in hearts of mice kept on a ketogenic diet for 2 weeks when compared to animals fed with a control diet (Fig 6A). Incorporation of 5‐ethynyl‐2ʹ‐deoxyuridine (EdU) into newly synthesized DNA is an alternative method to detect proliferating cells. EdU was administered orally starting from the day of switching the diet until the end of the study. This assay showed higher numbers of EdU‐positive cardiac ECs in mice fed a ketogenic diet when compared to animals that had received a control diet (Fig 6B). No differences in the abundance of apoptotic cells were determined by detection of cleaved caspase 3‐positive (Fig EV4A).

Next, we tested whether ketogenic diet would increase the number of proliferating cardiac ECs when given over a longer period. First, the number of Ki‐67‐positive ECs was determined after 4 weeks of feeding the respective diets. Again, there was a pronounced increase in Ki‐67‐positive ECs but no increase in apoptotic ECs in mice receiving a ketogenic diet (Figs 6C and EV4B). However, in mice that received ketogenic diet for 6 weeks, we could not detect in the number of proliferating ECs between the two groups anymore (Fig 6D). This indicates that ketogenic diet leads to a transient increase in cardiac EC proliferation. Consistently, we could not detect differences in the cardiac vessel density of animals kept on a ketogenic diet when compared to littermate animals kept on a control diet at different time points for up to 4 months (Figs 6E and EV4C–G). In addition, the ratio of EC to non‐EC cardiac cells was not changed. As such, ketone bodies induce a transient increase in the basal proliferation rate of cardiac ECs which does not result in an expanded blood vessel network.

### Ketogenic diet does not promote EC proliferation in other organs

We wondered whether the observed transient effect of ketogenic diet on EC proliferation in the heart would also occur in other vascular beds. Therefore, the ratio of Ki‐67‐positive versus negative ECs was determined. This revealed, that 2 weeks of ketogenic diet increased EC proliferation only in the heart (Fig 6A and B) but not in skeletal muscle (gastrocnemius and soleus), brain, liver, lung, or white adipose tissue (Fig 6F).

EC turnover is low in the quiescent vasculature of organs in the adult vasculature. However, in tumors, there is a reactivation of the embryo‐fetal angiogenic program leading to high EC proliferation rates (Rafii *et al*, 2016; Augustin & Koh, 2017). To investigate whether ketone bodies would affect angiogenesis, B16 melanoma cells were implanted into the skin of C57Bl/6J mice, a model leading to rapid formation of new blood vessels. There were no effects on tumor EC proliferation or vessel density in mice fed a ketogenic diet. In addition, tumor growth rates were not different between both diet groups (Fig EV4H–J).

### Proliferation of cardiac ECs is increased after pressure overload‐induced cardiac hypertrophy in mice kept on a ketogenic diet

The data suggest that ketogenic diet transiently increases the proliferation capacity of ECs specifically in the heart. Therefore, we wondered whether this might be beneficial in the diseased heart. Hemodynamic overload in response to, for example, hypertension, aortic stenosis, or myocardial infarction, can be partly compensated by a hypertrophic response. Pathological cardiac hypertrophy induces maladaptive cardiac remodeling and dysfunction characterized by vascular rarefaction amongst others (Nakamura & Sadoshima, 2018).

**Figure EV5:**
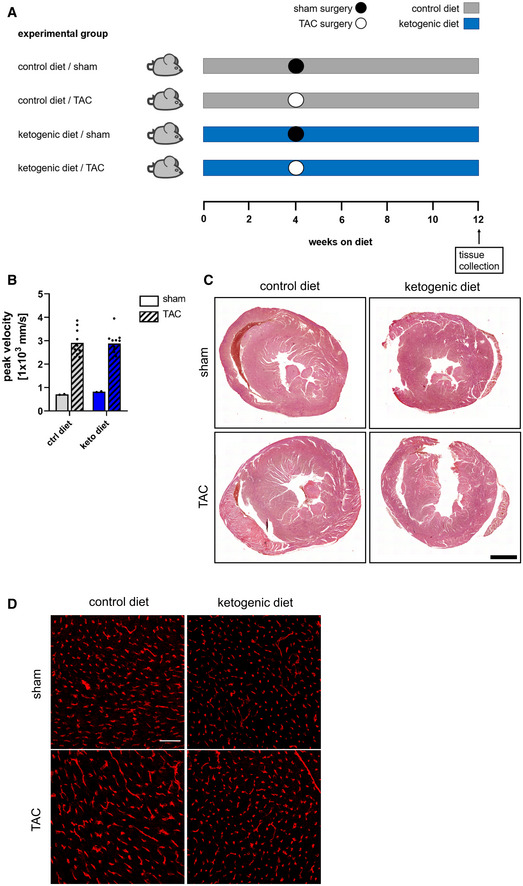
Schematic overview of experimental animal groups for transverse aortic constriction study and aortic flow rates Male and female C57Bl/6J mice were randomly assigned to a diet group at 8 weeks old. After 4 weeks on the respective diet, mice in each group underwent either transverse aortic constriction (TAC) or sham surgery. Mice were afterward kept on the respective diet for eight more weeks.Quantification of aortic blood flow rates within the stenosis of C57Bl/6J mice after TAC or sham surgery. Data are presented as mean *n* ≥ 2.Trichrome staining of heart sections from sham and TAC‐operated animals kept either on a control diet or ketogenic diet 8 weeks after surgery. Scale bar: 1 mm.Representative images of heart vasculature (CD31^+^ vessels) of mice kept on a control or ketogenic diet 8 weeks after TAC surgery. Scale bar: 50 µm. Data are presented as mean ± SD. Two‐tailed unpaired Student’s *t*‐test; **P < 0.01; ***P < 0.001.

**Figure 7:**
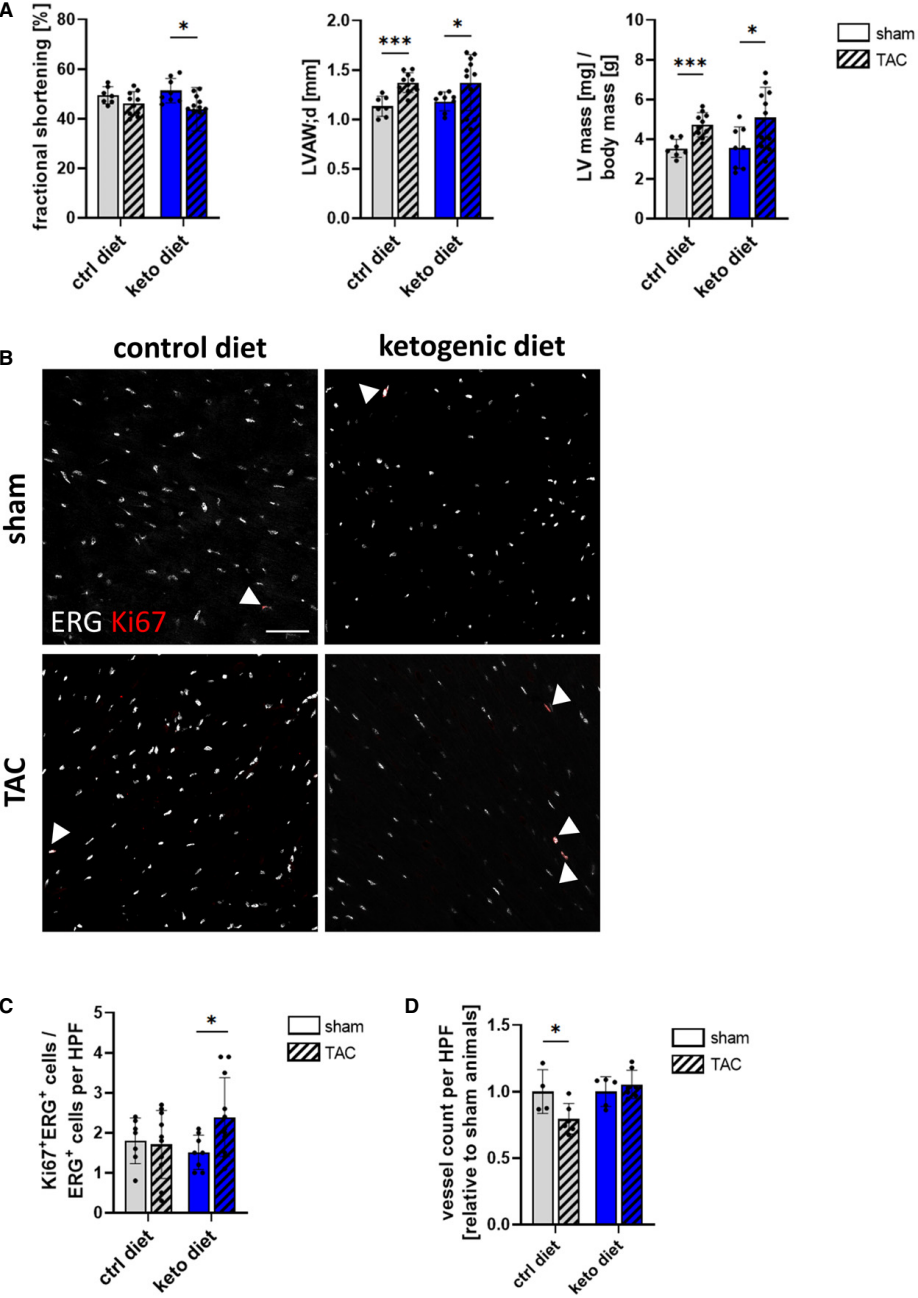
Ketogenic diet prevents vascular rarefaction in mice after transverse aortic constriction Parameters of cardiac function (fractional shortening, left ventricular end‐diastolic anterior wall thickness (LVAW;d), ratio of left ventricular (LV) mass to body mass) 8 weeks after transverse aortic constriction (TAC) in male and female C57Bl/6J mice receiving a control diet or ketogenic diet compared to sham animals kept on the same diet.Representative images of heart sections of mice kept on a control or ketogenic diet 8 weeks after TAC surgery stained against Ki67 and ERG.Quantification of Ki67^+^/ERG^+^ cells per high power field (HPF) in heart sections of mice kept on a control diet or a ketogenic diet 8 weeks after TAC surgery.Quantification of isolectin IB4‐positive vessels per high power field (HPF) in heart sections of mice kept on a control diet or a ketogenic diet 8 weeks after TAC surgery relative to sham animals from the respective diet group. Scale bar: 50 µm. Data are presented as mean ± SD. *n* ≥ 4; *P < 0.05; ***P < 0.001 unpaired Student’s *t*‐test.

We therefore investigated whether feeding a ketogenic diet in a prevention trial would affect vascular remodeling in an acute pressure overload model causing cardiac hypertrophy (Fig EV5A). Transverse aortic constriction (TAC) increased aortic flow rate within the stenosis in both diet groups to a similar degree (Fig EV5B) and led to thickening of the left ventricle 8 weeks after surgery in mice fed a control or ketogenic diet (Fig EV5C). Parameters for contractile cardiac function were unchanged between animals fed a control or ketogenic diet in the TAC‐operated and the sham group (Fig 7A). In the control diet group, there were no differences in the percentage of proliferating cardiac ECs between the sham and TAC‐operated animals (Fig 7B and C), indicating that, as expected, cardiac hypertrophy leads to vascular rarefaction. Indeed, mice on control diet showed lower blood vessel density in the hypertrophic left ventricular wall compared to sham‐operated mice (Figs 7D and EV5D). However, mice fed a ketogenic diet had higher rates of EC proliferation in the hypertrophic left ventricle upon TAC compared to sham mice also receiving ketogenic diet (Fig 7B and C). Consistently, blood vessel density was maintained between TAC and sham animals in the group fed with ketogenic diet whereas blood vessel density declined in the group fed with control diet (Figs 7D and EV5D).

Together the data indicate that ketone bodies increase the angiogenic potential of cardiac ECs which might help to maintain a dense blood vessel network in the heart in pathological conditions.

## Discussion

Ketone body metabolism is crucial for survival in situations of carbohydrate restriction. Here we report that vascular ECs, which are involved in the transport of these energy‐rich metabolites to ketone body‐oxidizing organs, oxidize ketone bodies via the TCA cycle. ECs display a great phenotypic plasticity that is tightly linked to their metabolic state (Potente & Carmeliet, 2017). The metabolic state of quiescent endothelial cells is characterized by high levels of anaerobic glycolysis as well as fatty acid oxidation. Activation of quiescent endothelial cells towards a proliferating or migrating phenotype requires metabolic adaptions (Wilhelm *et al*, 2016; Dumas *et al*, 2020). Angiogenic ECs further increase the rate of glycolysis, while fatty acid oxidation levels are decreased (De Bock *et al*, 2013). This highlights the importance of metabolic adaptions in regulating EC behavior that can even override genetic pre‐determination (Potente & Carmeliet, 2017).

Vascular ECs are continuously exposed to varying concentrations of ketone bodies in the blood. This raises the question whether the availability of ketone bodies affects EC behavior. We found that ketone body oxidation in ECs fuels energy as well as biomass production, resulting in increased proliferation, migration, and sprouting potential. This is in line with data from lymphatic ECs, where ketone body oxidation has comparably positive effects on lymphangiogenesis (Garcia‐Caballero *et al*, 2019). Mechanistically, the observed effects are dependent on ketone body oxidation via SCOT. Whether also signaling events through G protein‐coupled receptors such as HCAR2 or FFAR3 (Newman & Verdin, 2017) and epigenetic modification such as histone β‐hydroxybutyrylation or histone acetylation (Ruan & Crawford, 2018) are involved in regulating EC behavior in response to ketone body exposure needs to be evaluated. However, data presented in this study argue against it.

The heart represents a major consumer of ketone bodies (Puchalska & Crawford, 2017). Elevated levels of circulating ketone bodies by administration of a ketogenic diet increased the rates of EC proliferation exclusively in the heart of healthy adult mice. This induction was transient and did not affect cardiac vascular density. However, in a pathological model of cardiac hypertrophy, proliferation rates of cardiac endothelial cells were higher in mice receiving a ketogenic diet when compared to control diet‐fed mice in the long term. Ketone body oxidation rates are known to be increased in the failing murine and human heart (Lommi *et al*, 1996; Aubert *et al*, 2016; Bedi *et al*, 2016). Supplementation of ketone bodies to the failing heart, either by a ketogenic diet or by direct infusion, ameliorates pathological cardiac remodeling in animal models of heart failure (Horton *et al*, 2019). Likewise, elevating circulating ketone body levels by β‐hydroxybutyrate infusion improved cardiac function in a small cohort of heart failure patients (Nielsen *et al*, 2019). We did not observe a significant impairment of contractile function in our TAC model, consistent with compensated hypertrophy in the applied setting. Future studies will have to address whether ketogenic diet or administration of ketone bodies in prevention and intervention trials show beneficial outcome in various animal models of heart disease.

Notably, while cardiac vessel density decreased after TAC surgery in animals receiving a control diet, it was maintained in animals from the ketogenic diet group after TAC surgery. Vascular rarefaction is a characteristic sign of pathological cardiac hypertrophy associated with maladaptive cardiac remodeling and dysfunction (Nakamura & Sadoshima, 2018). It has been reported that the beneficial effects of ketone body supplementation during cardiac hypertrophy are attributable to ketone body oxidation in cardiomyocytes and that administration of ketone bodies enhances the respiratory efficiency of cardiomyocytes in the failing heart (Schugar *et al*, 2014; Uchihashi *et al*, 2017; Horton *et al*, 2019). Data presented within this study provide a rationale for further investigating the role of endothelial ketone body oxidation in the failing heart in maintaining a stable microvessel density, preventing vessel rarefication, and eventually ensuring sufficient transport of oxygen and nutrients to cardiomyocytes in pathological conditions.

## Materials and Methods

### Ethics approval

All animal procedures were performed in accordance with institutional and national regulations and approved by local committees for animal experimentation (RP Karlsruhe, DKFZ, Heidelberg University).

### Animal experiments

All animal procedures were performed in accordance with institutional and national regulations and approved by local committees for animal experimentation (RP Karlsruhe, DKFZ, Heidelberg University). Animals were housed under specific pathogen‐free barrier conditions at 21 ± 2°C with 60% humidity and a 12‐h light /dark rhythm. Wild‐type C57BL/6J mice were purchased from Janvier Labs. Eight‐week‐old mice were randomly assigned to the control diet or the ketogenic diet group and were offered the respective irradiated diet (D12359 with 10.5 kcal% protein, 77.9 kcal% carbohydrate, 11.6 kcal% fat; D17011501 with 10.4 kcal% protein, 0.1 kcal% carbohydrate, 89.5 kcal% fat; both from Research Diets) in addition to standard chow for 2 days. Afterward, mice were kept on either control diet or ketogenic diet only with *ad libitum* access to food and drinking water until the end of the experiment. A mixed cohort including male and female wildtype C57BL/6J mice was used for TAC experiments. Male wild‐type C57BL/6J mice were used for all other mouse experiments.

#### Cardiac EC proliferation

To analyze EC proliferation rates in the heart, EdU (Sigma‐Aldrich) dissolved in the drinking water at a final concentration of 0.2 mg/ml was orally administered to mice for 2 weeks from the day of switching the diet.

#### Tumor experiments

For tumor experiments, 1 × 10^6^ B16F10 melanoma cells in 100 µl PBS were subcutaneously injected into the abdominal flank of mice 2 weeks after switching the diet. Tumor growth was monitored over a time period of 10 days.

#### Transverse aortic constriction (TAC)

TAC was performed to investigate the effects of elevated ketone body serum concentrations on cardiac function during hypertrophy. Transthoracic echocardiography was performed on a Visual Sonics Vevo 2100 setup in awake mice to monitor heart function. Mice used in this study had received either a control diet or ketogenic diet for 4 weeks before surgery and were kept on the respective diet until the end of the study, 8 weeks after surgery. TAC surgeries were performed as previously described (Lehmann *et al*, 2014). Briefly, after mice had been anesthetized, lateral thoracotomy was performed. A 0.05 mm polypropylene suture was placed around the transverse aorta between the left carotid artery and the brachiocephalic artery. A 27‐gauge needle was placed parallel to the transverse aorta and the suture ligated against the needle. After removal of the needle, this created a constriction of 0.4 mm in diameter (stenosis). Sham animals underwent the same procedure; however, the transverse aorta was not ligated. Integrity of aortic constriction was confirmed by acquisition of transthoracic Doppler echocardiograms and subsequent analysis of blood flow patterns within the stenosis.

### Immunofluorescence

Mouse tissue was cryopreserved using O.C.T. and cut into 5 µm sections. Cryosections were fixed in ice‐cold methanol/acetone. After blocking in 10% goat serum with 0.1% Triton X‐100, sections were incubated with primary antibodies diluted in 10% goat serum at 4°C overnight. Following primary antibodies were used: anti‐CD31 (550274, BD Biosciences, 1:50), anti‐cleaved caspase 3 (9664, Cell Signaling Technology, 1:100), anti‐ColIV (2150‐1470, Bio‐Rad, 1:250), anti‐ERG (ab93513, Abcam, 1:1,000), anti‐α‐SMA (C6198, Sigma‐Aldrich, 1:200), anti‐Ki‐67 (14‐5698‐82, Thermo Fisher Scientific, 1:100) or FITC‐Isolectin B4 (L2895, Sigma‐Aldrich, 1:100). DAPI and fluorophore‐conjugated secondary antibodies were added before embedding the sections in fluorescent mounting medium (DAKO). Images were acquired using a confocal microscope (Zeiss LSM 700) and analysis was done using Fiji software.

### RNA‐sequencing of cardiac ECs

Heart tissue was digested in PBS containing 2 mg/ml Collagenase type II (Worthington), 2.4 mg/ml Dispase II (Sigma‐Aldrich) and 20 µl 1 M CaCl_2_ at 37°C for 1 h. After filtration, cardiac ECs were isolated using CD31 antibodies (550274, BD Biosciences) coupled to magnetic beads (Thermo Fisher Scientific). RNA was extracted using the RNeasy mini kit (Qiagen). Libraries were prepared with an input of 10 ng RNA per sample using the SMART‐Seq v4 Ultra Low Input RNA Kit for Sequencing (Takara). Samples were sequenced on a HiSeq 2000 v4 system (Ilumina) with a depth of 50 base pairs in single‐read mode. RNA sequencing data were processed using the DESeq2 package for detection of differentially expressed genes.

For gene set enrichment analysis (GSEA), a list containing all annotated genes detected by RNA‐sequencing with their corresponding log2 fold changes and *P*‐values was used (17,290 genes). Data were processed using the GSEA software (Broad Institute, Cambridge, MA, USA). Expression levels of genes involved in specific KEGG pathways in individual samples were visualized using heat maps generated with the Morpheus software (Broad Institute, Cambridge, MA, USA).

### Synthesis of acetoacetate

Acetoacetate was synthesized as described (Puchalska *et al*, 2019). In brief, 8 ml 1 M NaOH were added to 1 ml ethyl‐acetoacetate (Sigma‐Aldrich) while stirring at 60°C for 30 min. Using 50% HCl, the pH was adjusted to 7.5‐8. Synthesis of acetoacetate leads to generation of equimolar concentrations of ethanol. Therefore, ethanol was used in this concentration as control treatment for acetoacetate.

### Cell culture

Murine cardiac ECs (MCEC) were purchased from tebu‐bio and routinely tested for mycoplasma contamination. Cells were cultured in DMEM containing 1 g/l d‐glucose, 5% FCS, 5% HEPES, 100 units/ml penicillin and 100 μg/ml streptomycin. For experiments, MCEC were incubated until 80% confluent and starved for 1 h prior to the addition of reagents in DMEM containing 0.1% FCS. Reagents were added at indicated concentrations for either 24, 48, or 72 h, as indicated. Ketone body concentration in media was determined using a colorimetric total ketone body assay kit (Wako). Cell proliferation rates were determined using a chemiluminescent cell proliferation ELISA (Sigma‐Aldrich).

Human cardiac endothelial cells were purchased from Promocell and cultured in Endothelial Cell Growth Medium (Promocell). For experiments involving addition of recombinant proteins, cells were incubated in basal medium without the addition of growth factors or FCS. Cells were treated with recombinant VEGF (10 ng/ml for 6 h) or recombinant murine or human IL1b, IL‐6, or TNF‐α (50 ng/ml for 6 h). Serum‐starved cells were cultured for 24 h in basal medium containing 0.1% FCS and compared to cells cultured in basal medium containing 5% FCS. Hypoxia was induced upon addition of 100 µM Cobalt(II) chloride hexahydrate (Santa Cruz) for 24 h.

Adipose tissue ECs and HUVECs were isolated from biopsies or umbilical cords and collected from patients at the University Hospital Heidelberg. Collection was approved by the Institutional Review Board of the Medical Faculty of the University of Heidelberg. Informed consent was obtained from all subjects and experiments conformed to the principles set out in the WMA Declaration of Helsinki and the Department of Health and Human Services Belmont Report. Adipose tissue ECs were isolated from abdominal adipose tissue biopsies and collected from patients undergoing bariatric surgery at the University Hospital Heidelberg. Human umbilical cord endothelial cells (HUVEC) were freshly isolated as previously described (Adam *et al*, 2013). HUVEC isolated from 3 donors were pooled. Cells were cultured in Endopan‐3 medium (PAN Biotech).

For culture of primary murine ECs from C57BLJ mice, heart, lung, muscle, and adipose tissue were digested in PBS containing 2 mg/ml Collagenase type II (Worthington), 2 mg/ml Dispase II (Sigma‐Aldrich) at 37°C for 0.5–1 h. After 100 µM filtration, ECs were isolated using CD31 antibodies (550274, BD Biosciences) coupled to magnetic beads (Thermo Fisher Scientific). Isolated CD31+ cells were cultured in Endopan‐3 medium (PAN Biotech).

### Lentiviral transduction

SCOT‐deficient cells were generated using the CRISPR/Cas9 system. Hence, Oxct1 guide oligonucleotides were cloned into the pLentiCRISPRv2 vector according to Ran *et al* (2013). Following guide oligos were used (designed with Benchling software): construct 1: guide 1 forward: caccgacgaatgatctcctcatatg, guide 1 reverse: aaaccatatgaggagatcattcgtc, guide 2 forward: caccgtggatcgaagtaaaaggccc, guide 2 reverse: aaacgggccttttacttcgatccac; construct 2: guide 1 forward: caccgagggtatgggactctggtac, guide 1 reverse: aaacgtaccagagtcccataccctc, guide 2 forward: caccgtccaccggcacggatcctct, guide 2 reverse: aaacagaggatccgtgccggtggac. A non‐targeting pLenti construct was used as control. Lentiviral particles were produced in HEK293T cells and used to transduce MCEC.

### Real‐time cell analysis

Proliferation and migration of MCEC were measured real‐time using an xCELLigence device (Agilent) that monitors electrical impedance. For analysis of proliferation, 10,000 cells were added per well on an E‐plate (Agilent) and media contained 2% FCS and 10 mM acetoacetate, 10 mM β‐hydroxybutyrate (Sigma‐Aldrich) or the respective volumes of water or 1 M ethanol. Impedance was measured every 15 min for 24 h. Migration was analyzed using CIM‐plates (Agilent) with a pore size of 8 µm. 30,000 cells were added per well. Media in the upper chamber was serum‐free, while media in the lower chamber contained 2% FCS. 10 mM acetoacetate, 10 mM β‐hydroxybutyrate or the respective volumes of water or 1 M ethanol were added to the media in the upper and lower chamber. Electronic impedance in CIM plates was monitored every 15 min for a total of 24 h.

### Untargeted metabolomics

For stable isotope labeling, MCEC were incubated in DMEM containing 10 mM glucose and 2 mM glutamine with either 1 mM uniformly labeled sodium D‐[^13^C_4_]‐β‐hydroxybutyrate or 1 mM uniformly labeled [^13^C_4_]‐acetoacetate for 24 h. Samples were collected in methanol and extracted and analyzed as previously described (Puchalska *et al*, 2019). In brief, isotope‐labeled samples were extracted by performing three cycles of vortexing, freeze‐thawing and sonication. The protein‐free samples were analyzed by coupled liquid chromatography‐mass spectrometry. Liquid chromatography was performed using a Vanquish Horizon system (Thermo Fisher Scientific) with a Luna NH2 column (Phenomenex) with hydrophilic interaction liquid chromatography and mass spectrometry was performed on a Thermo Q Exactive Plus (Thermo Fisher Scientific) operated in negative mode. Data were processed using the Compound Discoverer 3.0 software. Lipid‐species identities are putative. TCA cycle intermediates, amino acids, and UDP species are identified by use of standards and MS/MS fragmentation pattern matching to open source databases.

### Quantification of TCA cycle intermediates

TCA cycle intermediates were quantified in MCEC treated with 2 mM acetoacetate or 2 mM D‐β‐hydroxybutyrate (Sigma‐Aldrich) or the respective controls for 24 h. Samples were extracted in methanol with sonication and the separation protocol was adapted from Uran *et al* (2007). In brief, samples were analyzed on an Acquity HSS T3 column (Waters) heated to 40°C connected to an Acquity H‐class UPLC system (Waters). The UPLC system was coupled to a QDa mass detector (Waters) in single ion record mode, using negative detector polarity and 0.8 kV capillary voltage. Data acquisition and processing was performed with the Empower3 software suite (Waters).

### Seahorse bioanalyzer

Mitochondrial respiration was measured using the Seahorse Bioanalyzer (Aligent Seahorse, XF96 Bioanlyzer) (Pike Winer & Wu, 2014). Briefly, MCECs were re‐seeded into XF96cell culture plate (Agilent Seahorse XF, XF96 FluxPack) at density of 25,000 cell per well in normal growth media (5% FCS in DMEM 1 g/l glucose) and allowed to adhere. The media was then exchanged to the stimulation media (0.1% FCS in DMEM 1 g/l glucose) containing the stated ketone bodies or respective vesicle controls and incubated for further 12 h. The media was then exchanged to the Agilent Seahorse XF Assay Medium (Agilent Seahorse, 102353‐100) supplemented with 10 mM glucose, 2 mM glutamine, 1 mM sodium pyruvate and incubated for 1 h prior to assay in a non‐CO_2_ incubator at 37°C. Injections of oligomycin (2 µM final), FCCP (1 μM final), and a combination of rotonone and antimycin‐A (0.5 μM final each) were diluted in the Agilent Seahorse XF Assay Medium and loaded onto ports A, B and C respectively. The bioanalyzer was calibrated and the assay was performed using Mito Stress Test protocol as suggested by the manufacturer (Agilent Seahorse Bioscience). The assay was run in one 96‐well plate with 16 replicates per condition. Seahorse Wave software was used to analyze metabolic data generated from both assays. The data from each assay were normalized to the total protein content in each well, as measured by the Bradford assay following addition of lysis buffer (100 mM sodium phosphate (pH7.4) + 0.1% Triton X100; 50 µl per well) and two cycles of freeze‐thawing.

### ATP measurements

The ATP content of the ECs treated with the stated ketone bodies or respective controls for 12 h was measured using the ATPlite Luminescence Assay System (Perkin Elmer), according the manufacturer’s instructions. The assay was run in one 96‐well plate with 8 replicates per condition.

UPLC analysis for the quantification of nucleotides including ATP was performed at the Metabolomics Core Technology Platform of Heidelberg University. 5x10^6^ cells were lysed in 0.37 ml ice‐cold 0.5 M perchloric acid with sonication. After neutralization with 86 µl ice‐cold buffer (2.5 M KOH, 1.5 M K_2_HPO_4_) samples were centrifuged (16,400 *g* for 10 min at 4°C) and filtered (0.2 µm filters). Analysis was done on an Aquity HSS T3 column (Waters) connected to an Acquity H‐class UPLC system. Column temperature was 40°C. Solvent A (50 mM potassium phosphate buffer, 8 mM tetrabutylammonium hydrogensulfate, pH 6.5). The elution gradient was as follows: after 2.6 min, 0% solvent B (acetonitrile in 70% solvent A) to 17 min with 77% solvent B, hold for 1 min at 77% solvent B, followed by return to 0% solvent B and conditioning of the column to initial conditions for 10 min. Nucleotides were detected using an Acquity PDA detector (Waters, 260 nm) and ultrapure standards (Sigma Aldrich).

### Spheroid‐based sprouting assay

MCEC were resuspended in growth medium containing 20% methocel (Sigma‐Aldrich). For spheroid formation, hanging drops containing approximately 200 cells were incubated for 24 h as described (Heiss *et al*, 2015). Spheroids were then resuspended in methocel with 20% FCS and mixed with an equal volume of collagen solution. After polymerization of the collagen matrix, 30 mM D‐β‐hydroxybutyrate, 30 mM L‐β‐hydroxybutyrate, 30 mM acetoacetate, 10 mM acetate, 1 mM butyrate, 1 mM octanoate, 1 mM niacin or an equal volume of ddH_2_O or 1 M ethanol was added in 100 µl basal DMEM. Please note that after diffusion into the matrix, concentration of reagents is diluted to approximately 10% of the mentioned concentration, since the ratio of medium to collagen matrix is approximately 1/9. Spheroids embedded in the collagen matrix were incubated for 48 h and then fixed with 1 ml 10% PFA per well. At least ten spheroids per treatment were imaged using an inverted microscope (Leica DM IRB) and sprout length was analyzed using Fiji software.

### Single‐cell transcriptome analysis

Publically available endothelial cell atlas database (https://endotheliomics.shinyapps.io/ec_atlas/) was accessed to extract t‐SNE plots for the expression of *Oxct1* in cardiac and non‐cardiac endothelial cells (Kalucka *et al*, 2020).

### cDNA synthesis and qPCR

cDNA was synthesized using a reverse transcriptase kit (Applied Biosystems). mRNA expression was analyzed in doublets using Sybr Green Master Mix (Thermo Fisher Scientific) by QuantStudio 3 (Thermo Fisher Scientific). Gene expression was normalized relative to the housekeeping gene *OAZ1* for human ECs or *Cph* for murine ECs using the 2^−ΔΔCt^ method. The following primers were used for qRT–PCR:

**Table:** 

Name	Forward primer	Reverse primer
*hOAZ1*	GAGCCGACCATGTCTTCATT	CTCCTCCTCTCCCGAAGACT
*hBDH1*	GAAAATGGAGCAAGACGCCC	GCATAAGTCCGACGGCCAAT
*hOXCT1*	GTGTCCAGTGCGAAAACCAA	ACACGCAGCCTGGTACAAAT
*mCph*	ATGGTCAACCCCACCGTG	TTCTTGCTGTCTTTGGAACTTTGTC
*mBdh1*	CGCACCGGAGTGTGTGTAAG	GGCACCAAGTTGTAAGACGC
*mOxct1*	CTGGAGTTTGAGGACGGCAT	TCCGCATCAGCTTCGTCTTT
*mPdk4*	GAGCTGTTCTCCCGCTACAG	CGGTCAGGCAGGATGTCAAT
*mHmgcs2*	AGAAATCCCTGGCTCGGTTG	CTTGGGCAGAGTGGTGAGAG

### Western blot analysis

Cells were lysed with Cell Lysis Buffer (9803S, Cell Signaling Technology) containing 1 mM PMSF. Whole tissue lysates were generated using bead mills with 100 µl lysis buffer per 50 mg of tissue. SDS–PAGE was employed to separate proteins which were then blotted on a nitrocellulose membrane. Membranes were blocked in 5% skim milk dissolve in TBS with 0.1% Tween 20. Membranes were incubated with primary antibodies diluted in 5% skim milk in TBST overnight at 4°C. Following primary antibodies were used: anti‐β‐Actin (A5441, Sigma‐Aldrich, 1:3,000), anti‐BDH1 (15417‐1‐AP, Proteintech for Fig 1B, 1:2,000 or ab193156, Abcam, 1:1,000 for all other Western blots), anti‐SCOT (12175‐1‐AP, Proteintech, 1:1,000), anti‐β‐Tubulin (2146, Cell Signaling Technology, 1:2,500), anti‐VCP (ab11433, Abcam, 1:2,000). HRP‐conjugated secondary antibodies (DAKO) were used and chemiluminescence was detected by Aceglow ECL Western Blotting Substrate (VWR International).

### Statistical analysis

Statistical analysis was performed using GraphPAD Prism 8.2.1 (GraphPAD Software Inc.). Statistical significance between groups was calculated using unpaired Student’s *t*‐test with *P*‐values < 0.05 considered statistically significant. Calculated *P*‐values are provided in the Appendix.

## Author contributions

**Eva‐Maria Weis:** Conceptualization; Investigation; Methodology; Writing—original draft. **Patrycja Puchalska:** Investigation; Methodology. **Alisa B Nelson:** Investigation; Methodology. **Jacqueline Taylor:** Investigation; Methodology. **Iris Moll:** Investigation; Methodology. **Sana Safatul Hasan:** Investigation; Methodology. **Matthias Dewenter:** Investigation; Methodology. **Marco Hagenmueller:** Investigation; Methodology. **Thomas Fleming:** Investigation; Methodology. **Gernot Poschet:** Investigation; Methodology. **Agnes Hotz‐Wagenblatt:** Investigation; Methodology. **Johannes Backs:** Methodology. **Peter A Crawford:** Conceptualization; Supervision; Methodology. **Andreas Fischer:** Conceptualization; Supervision; Funding acquisition; Methodology; Writing—original draft; Project administration; Writing—review & editing.

## Disclosure and competing interests statement

The authors declare that they have no conflict of interest.

## Supporting information



Appendix

Expanded View Figures PDF

Dataset EV1

Source Data for Figure 1

Source Data for Figure 4A

## Data Availability

All data generated or analyzed during this study are included in this published article. Raw RNA‐sequencing data have been deposited to Gene Expression Omnibus http://www.ncbi.nlm.nih.gov/geo/query/acc.cgi?acc=GSE191173 (accession number GSE191173).
