# Supplementary material for: Ketone body oxidation increases cardiac endothelial cell proliferation
Source: EMBO Mol Med. 2022 Feb 18;14(4):e14753. doi: 10.15252/emmm.202114753 (PMC8988203; doi:10.15252/emmm.202114753)
Supplement: Supplementary file 1 — Appendix [file EMMM-14-e14753-s002.pdf]

Appendix

Table of Content

Appendix Item	Pages
Appendix Fig. S1 & legend	2-3
Appendix Fig. S2 & legend	4
Appendix Table S1	5-8

Appendix Figure S1. Oxct1 expression in murine endothelial cells based on single cell RNA-sequencing.
(**A**) t-SNE plot showing the expression of Oxct1 in endothelial cells derived from different organs. Color scale: Purple - high expression; Yellow - low expression. (**B**) t-SNE plot showing the expression of Oxct1 in different sub-clusters of cardiac endothelial cells. Color scale: Purple - high expression; Yellow - low expression.

Appendix Figure S1

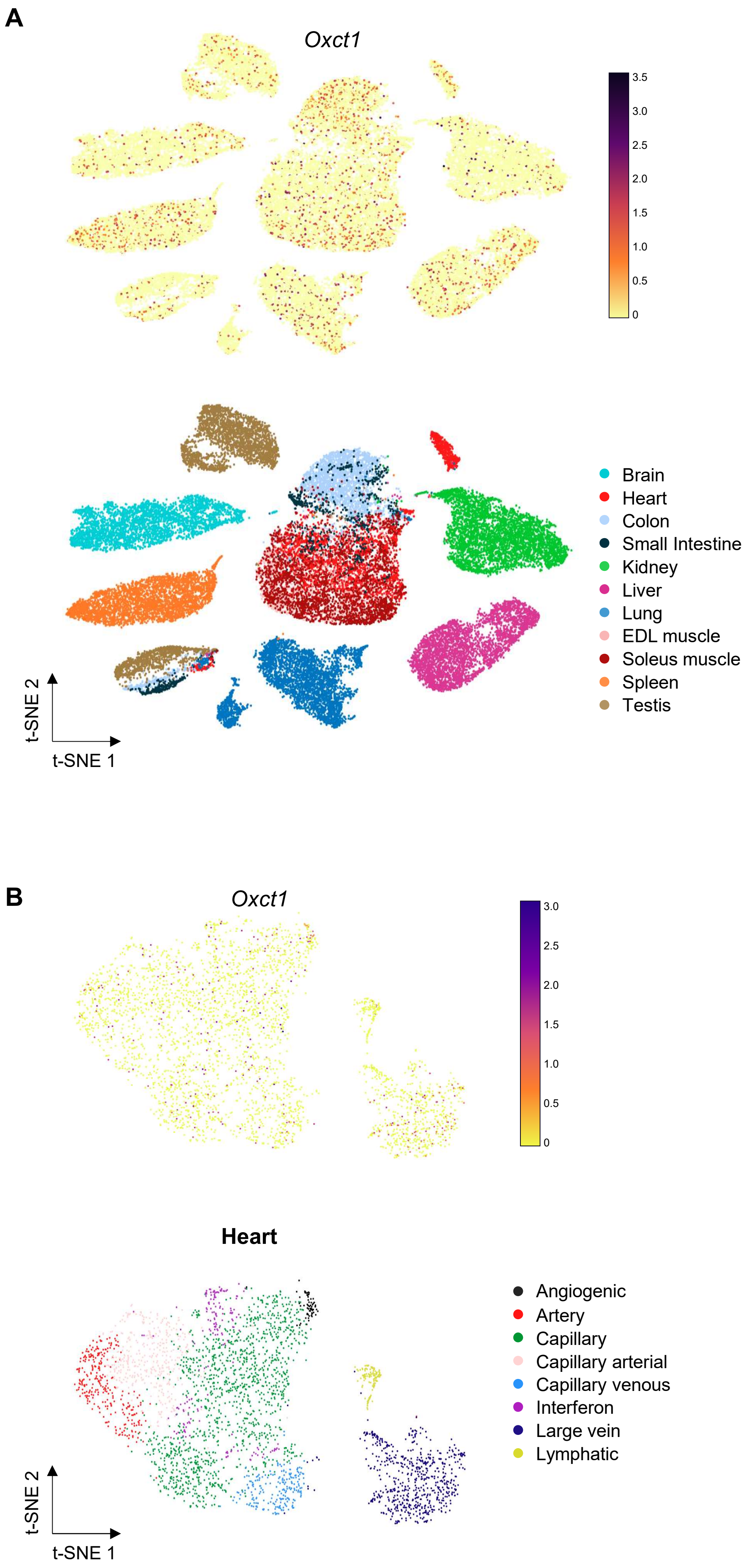

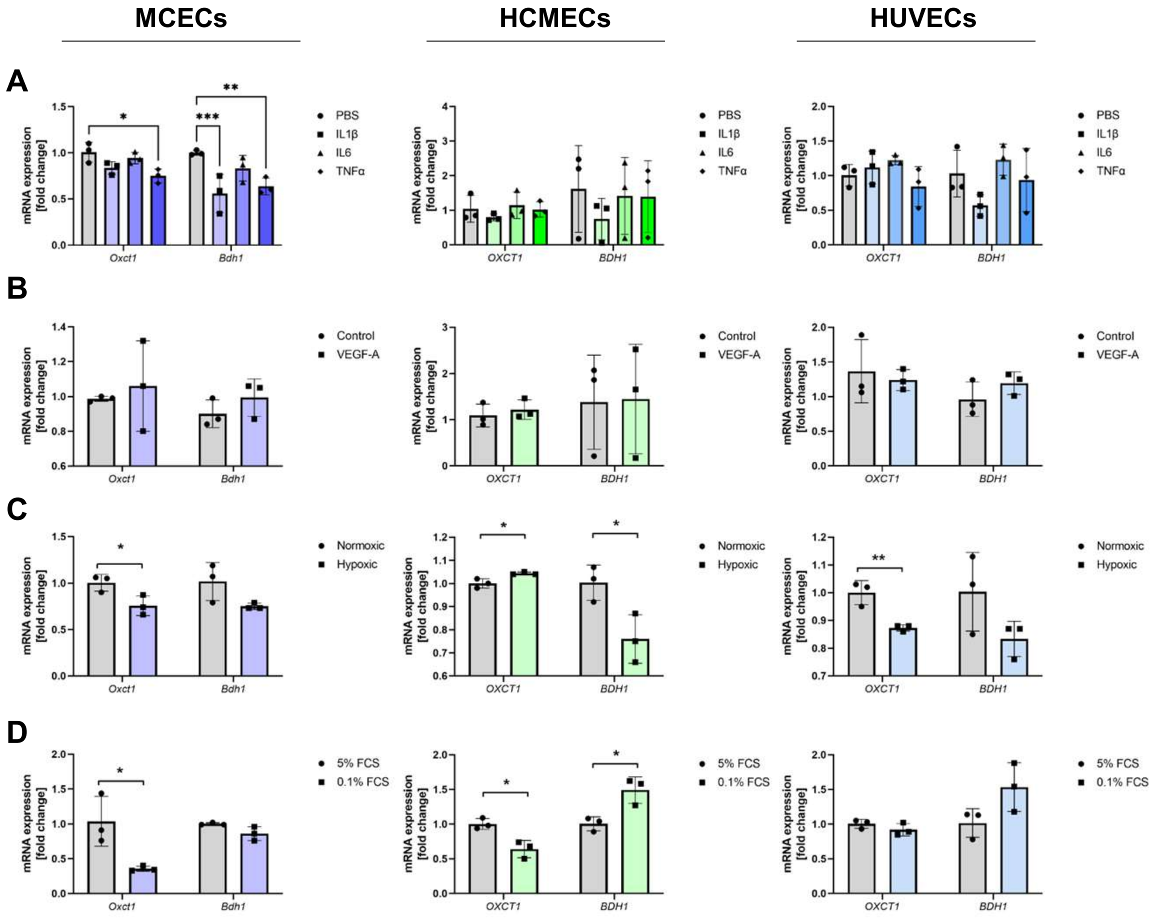

Appendix Figure S2.

Gene expression analysis of SCOT (OXCT) and BDH1 analyzed by qPCR in MCEC, HCMEC and HUVEC treated with (**A**) cytokines IL-1β, IL-6 or TNFα (**B**) recombinant VEGF or (**C**) cobalt-chloride to chemically induce hypoxia. (**D**) Gene expression was analyzed following 24 hours of serum starvation. Data are presented as mean ± SD. n=3; statistical significance determined using two-way ANOVA (A) or unpaired Student's t-test (B-D). *: $p<0.05$.

Appendix Table S1

Figure	Panel	Condition	P-value
1	I	24 vs 0 hours	0.0199
	J	24 vs 0 hours	<0.0001
2	A	citrate	0.000046
		α-ketoglutarate	0.554562
		succinate	0.119506
		fumarate	0.249687
		malate	0.05989
	B	citrate	0.000522
		α-ketoglutarate	0.046676
		succinate	0.857386
		fumarate	0.008569
		malate	0.021871
	C	citrate (βOHB)	5.3E-10
		citrate (AcAc)	4.2E-10
		α-ketoglutarate (βOHB)	0.019581
		α-ketoglutarate (AcAc)	2.71E-07
		L-malate (βOHB)	2.39E-11
		L-malate (AcAc)	2.92E-08
	D	Asparagine (βOHB)	3.65E-07
		Asparagine (AcAc)	2.25E-08
		L-aspartate (βOHB)	0.000187
		L-aspartate (AcAc)	2.53E-05
		L-glutamate (βOHB)	1.94E-10
		L-glutamate (AcAc)	9.97E-08
		Proline (βOHB)	8.91E-08
		Proline (AcAc)	5.23E-09
		Nervonic acid (βOHB)	0.095204
		Nervonic acid (AcAc)	0.000103
		1-palmitoylglycerol (βOHB)	1.04E-05
		1-palmitoylglycerol (AcAc)	1.38E-06
		Ceramide (d18:1/16:0) (βOHB)	0.000138
		Ceramide (d18:1/16:0) (AcAc)	0.000135
		Uridine monophosphate (βOHB)	1.3E-05
		Uridine monophosphate (AcAc)	1.56E-07
		Uridine 5'-diphosphate (βOHB)	2.71E-08
		Uridine 5'-diphosphate (AcAc)	1.15E-08
		UDP-N-acetylglucosamine (βOHB)	1.23E-09
		UDP-N-acetylglucosamine (AcAc)	8.13E-08
3	A	0 vs 0.1 mM	0.075292
		0 vs 1 mM	0.040979
		0 vs 10 mM	0.035186
	B	0 vs 0.1 mM	0.223811
		0 vs 1 mM	0.015466
		0 vs 10 mM	0.011592
	C	4 hours (H_20 vs βOHB)	0.020476
		8 hours (H_20 vs βOHB)	0.02311
		12 hours (H_20 vs βOHB)	0.01194
		16 hours (H_20 vs βOHB)	0.016379
		20 hours (H_20 vs βOHB)	0.012062
		24 hours (H_20 vs βOHB)	0.011128
	D	4 hours (EtOH vs AcAc)	0.105833
		8 hours (EtOH vs AcAc)	0.07712
		12 hours (EtOH vs AcAc)	0.071982
		16 hours (EtOH vs AcAc)	0.047972
		20 hours (EtOH vs AcAc)	0.037242
		24 hours (EtOH vs AcAc)	0.022273

	E	4 hours (H_20 vs βOHB)	0.006009
		8 hours (H_20 vs βOHB)	0.011297
		12 hours (H_20 vs βOHB)	0.007125
		16 hours (H_20 vs βOHB)	0.014617
		20 hours (H_20 vs βOHB)	0.051728
		24 hours (H_20 vs βOHB)	0.085801
	F	4 hours (EtOH vs AcAc)	0.012705
		8 hours (EtOH vs AcAc)	0.034425
		12 hours (EtOH vs AcAc)	0.005271
		16 hours (EtOH vs AcAc)	0.015564
		20 hours (EtOH vs AcAc)	0.047082
		24 hours (EtOH vs AcAc)	0.300952
	I	H_20 vs βOHB	0.0334
		EtOH vs AcAc	0.0467
	J	H_20 vs βOHB	0.0108
		EtOH vs AcAc	0.0003
	K	H_20 vs βOHB	0.0473
		EtOH vs AcAc	0.0051
4	B	Oxct1 ko1 vs control (βOHB)	0.004659
		Oxct1 ko1 vs control (AcAc)	0.048722
		Oxct1 ko2 vs control (βOHB)	0.004659
		Oxct1 ko2 vs control (AcAc)	0.026239
	C	Oxct1 ko1 vs control (βOHB)	0.010049
		Oxct1 ko1 vs control (AcAc)	0.194178
		Oxct1 ko2 vs control (βOHB)	0.015731
		Oxct1 ko2 vs control (AcAc)	0.018213
5	A	3 vs 0 days	4.07E-08
		7 vs 0 days	2.15E-06
		14 vs 0 days	6.35E-05
		28 vs 0 days	0.0007
6	A	keto vs control diet	0.0084
	B	keto vs control diet	0.0071
	C	keto vs control diet (4 weeks)	<0.0001
	D	keto vs control diet (6 weeks)	0.9618
	E	keto vs control diet (4 months)	0.3078
	F	keto vs control diet (gastrocnemius)	0.581946
		keto vs control diet (soleus)	0.365422
		keto vs control diet (brain)	0.053585
		keto vs control diet (lungs)	0.639473
		keto vs control diet (adipose tissue)	0.686375
		keto vs control diet (liver)	0.253563
7	A	fractional shortening: control diet (sham vs TAC)	0.134083
		fractional shortening: keto diet (sham vs TAC)	0.044592
		LVAW;d: control diet (sham vs TAC)	0.000238
		LVAW;d: keto diet (sham vs TAC)	0.023562
		LV mass: control diet (sham vs TAC)	0.000551
		LV mass: keto diet (sham vs TAC)	0.026048
	C	control diet (sham vs TAC)	0.810821
		keto diet (sham vs TAC)	0.031356
	D	control diet (sham vs TAC)	0.047
		keto diet (sham vs TAC)	0.49

EV1	B	H_2O vs 2mM βOHB	0.021
		EtOH vs 2mM AcAc	<0.001
	C	H_2O vs 2mM βOHB	0.099
		EtOH vs 2mM AcAc	0.006
	D	H_2O vs 2mM βOHB	0.017
		EtOH vs 2mM AcAc	<0.001
	E	H_2O vs 2mM βOHB	0.567
		EtOH vs 2mM AcAc	0.199
	F	H_2O vs 2mM βOHB	0.053
		EtOH vs 2mM AcAc	0.002
	G	H_2O vs 2mM βOHB	0.011
		EtOH vs 2mM AcAc	<0.001
	H	H_2O vs 2mM βOHB	0.121
		EtOH vs 2mM AcAc	0.024
EV2	A	0 vs 0.1 mM	0.0158
		0 vs 1 mM	0.0102
		0 vs 10 mM	0.0006
	B	0 vs 0.1 mM	0.0059
		0 vs 1 mM	0.0064
		0 vs 10 mM	0.00064
	C	0 vs 0.1 mM	0.012
		0 vs 1 mM	0.0005
		0 vs 10 mM	0.0041
	D	0 vs 0.1 mM	0.018
		0 vs 1 mM	0.0004
		0 vs 10 mM	0.016
	E	0 vs 0.1 mM	0.138
		0 vs 1 mM	0.0097
		0 vs 10 mM	0.028
	F	0 vs 0.1 mM	0.136
		0 vs 1 mM	0.048
		0 vs 10 mM	0.109
	H	Basal vs. VEGF	0.001
		Basal vs. 30 mM βOHB	0.056
EV3	A	Myh6 (heart lysate vs. αCD31-isolated cells)	<0.0001
		Acta2 (heart lysate vs. αCD31-isolated cells)	<0.0001
		SM22 (heart lysate vs. αCD31-isolated cells)	<0.0001
		Col3a1 (heart lysate vs. αCD31-isolated cells)	<0.0001
	C	Oxct1- 3 days (control vs ketogenic diet)	0.0004
		Oxct1- 7 days (control vs ketogenic diet)	1.5e-0.9
		Oxct1- 14 days (control vs ketogenic diet)	0.5262
		Bdh1- 3 days (control vs ketogenic diet)	0.8624
		Bdh1- 7 days (control vs ketogenic diet)	0.9799
		Bdh1- 14 days (control vs ketogenic diet)	0.6542
	D	Hmgcs2 - control vs ketogenic diet	0.076113
		Pdk4 - control vs ketogenic diet	0.007178

EV4	A	2 weeks - control vs ketogenic diet	0.2997
	B	4 weeks - control vs ketogenic diet	0.4248
	D	2 weeks - control vs ketogenic diet	0.9016
	E	4 weeks - control vs ketogenic diet	0.2823
	F	6 weeks - control vs ketogenic diet	0.1162
	G	4 months - control vs ketogenic diet	0.3078
	H	Ki67+CD31+ cells per HPF - control vs ketogenic diet	0.387122
	I	CD31+ vessels per HPF - control vs ketogenic diet	0.251
	J	6 days- tumor volume - control vs ketogenic diet	0.580729
		9 days- tumor volume - control vs ketogenic diet	0.598494
		10 days- tumor volume - control vs ketogenic diet	0.61933
Appendix Fig. S1	A	MCECs	
		Oxct1	
		PBS vs. IL1β	0.179
		PBS vs. IL6	0.8216
		PBS vs. TNFα	0.0292
		Bdh1	
		PBS vs. IL1β	0.0005
		PBS vs. IL6	0.1906
		PBS vs. TNFα	0.0025
		HCMECs	
		OXCT1	
		PBS vs. IL1β	0.9769
		PBS vs. IL6	0.9984
		PBS vs. TNFα	>0.9999
		BDH1	
		PBS vs. IL1β	0.5131
		PBS vs. IL6	0.9879
		PBS vs. TNFα	0.9833
		HUVECs	
		OXCT1	
		PBS vs. IL1β	0.9208
		PBS vs. IL6	0.6349
		PBS vs. TNFα	0.7939
		BDH1	
		PBS vs. IL1β	0.1208
		PBS vs. IL6	0.6859
		PBS vs. TNFα	0.9488
	B	MCECs	
		Oxct1	0.651295
		Bdh1	0.291537
		HCMECs	
		OXCT1	0.539925
		BDH1	0.944727
		HUVECs	
		OXCT1	0.671234
		BDH1	0.246606
	C	MCECs	
		Oxct1	0.037004
		Bdh1	0.090733
		HCMECs	
		OXCT1	0.022646
		BDH1	0.031708
		HUVECs	
		OXCT1	0.008247
		BDH1	0.13113
	D	MCECs	
		Oxct1	0.030547
		Bdh1	0.075225
		HCMECs	
		OXCT1	0.012862
		BDH1	0.017553
		HUVECs	
		OXCT1	0.258589
		BDH1	0.092038
