# Supplementary material for: Ketone body oxidation increases cardiac endothelial cell proliferation
Source: EMBO Mol Med. 2022 Feb 18;14(4):e14753. doi: 10.15252/emmm.202114753 (PMC8988203; doi:10.15252/emmm.202114753)
Supplement: Supplementary file 2 — Expanded View Figures PDF [file EMMM-14-e14753-s003.pdf]

Expanded View Figures

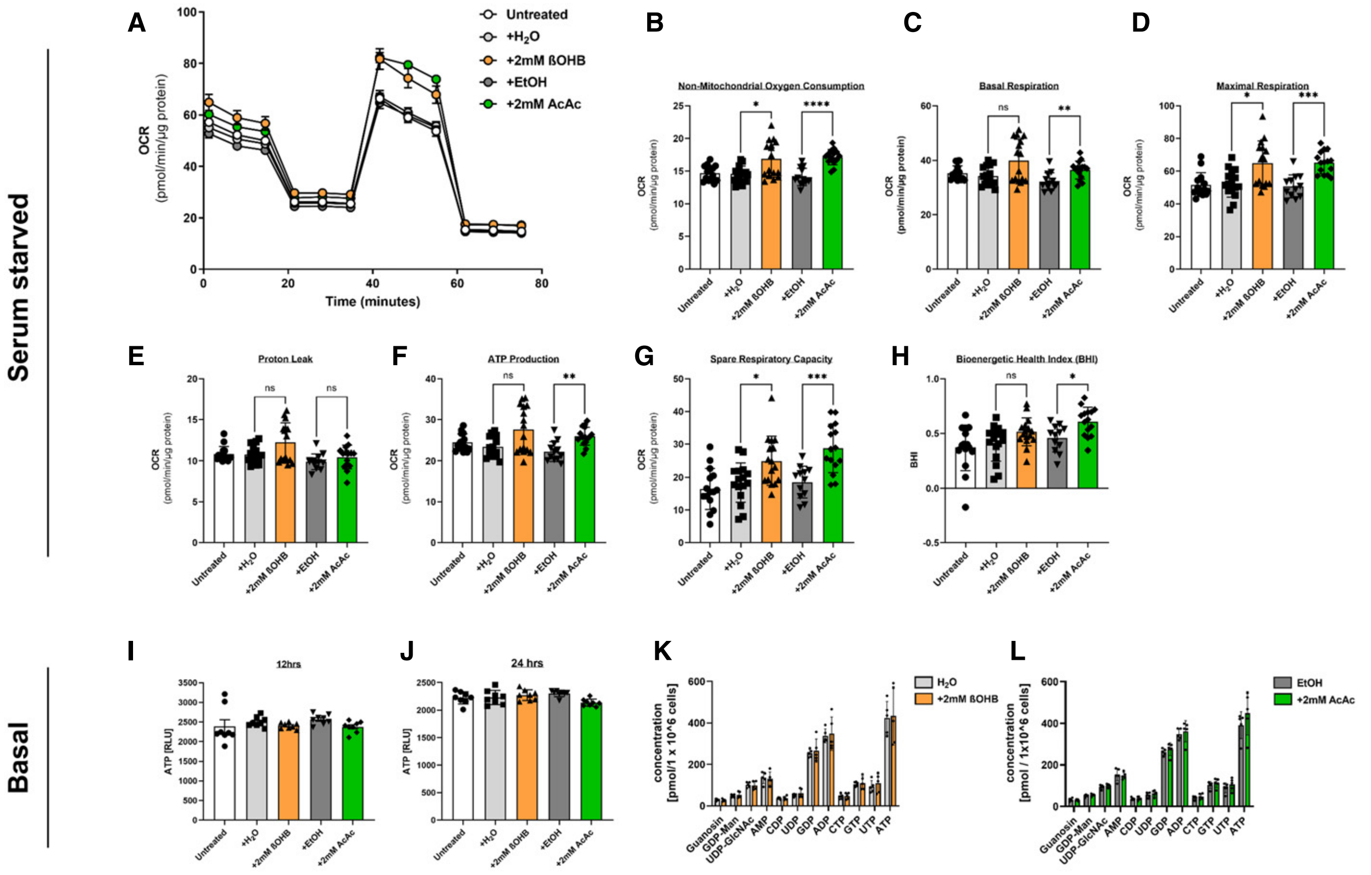

Figure EV1. Effects of ketone bodies on mitochondrial respiration.

Mitochondrial function of MCECs stimulated with ketone bodies was characterized using the Seahorse Bioanalyzer by measuring the rate of oxygen consumption (OCR) following sequential additions of oligomycin, FCCP and antimycin/rotenone (A) to the cells to determine non-mitochondrial oxygen consumption (B), basal respiration (C), maximal respiration (D), proton leakage (E), ATP production (F), spare respiratory capacity (G), and the bioenergetic health index (H), respectively.

A–H MCECs were starved for 1 h before addition of 2 mM R-β-hydroxybutyrate (βOHB) compared to H_2O and acetoacetate (AcAc) compared to ethanol for 24 h.

I–L MCECs were cultured in basal medium before addition of ketone bodies (2 mM) and solvent control. (I, J) Cellular ATP contents after 12 and 24 h. (K, L) Targeted metabolomics using UPLC to determine cellular nucleotides upon treatment with ketone bodies for 24 h. Data are presented as mean ± SD. One-way ANOVA using nonparametric (Kruskal–Wallis) test. ****$P < 0.0001$, ***$P < 0.001$, **$P < 0.01$, *$P < 0.05$, NS > 0.05.

Figure EV2. Effects of ketone body supplementation on sprouting potential.

A–C Cell counts of MCECs treated with 0.1, 1 or 10 mM R-β-hydroxybutyrate (βOHB) for 24, 48, or 72 h compared to treatment with H_2O (control).

D–F Cell counts of MCECs treated with 0.1, 1 or 10 mM acetoacetate (AcAc) for 24, 48, or 72 h compared to treatment with ethanol (control).

G Representative images of HUVEC spheroids treated with H_2O (control), recombinant VEGF-A165, 30 mM R-β-hydroxybutyrate (βOHB) for 72 h; scale bar: 50 µm.

H Quantification of the accumulated total sprout length per spheroid. $n = 4$.

I Relative absorbance of BrdU incorporated into DNA of HUVEC treated with 1, 4 or 10 mM R-β-hydroxybutyrate (βOHB), 0.1, 1 or 10 mM acetoacetate (AcAc) or a combination of both for 24 h. Data are presented as mean ± SD. $n \geq 2$. One-way ANOVA; *$P < 0.05$; **$P < 0.01$; ***$P < 0.001$.

▶

© 2022 The Authors

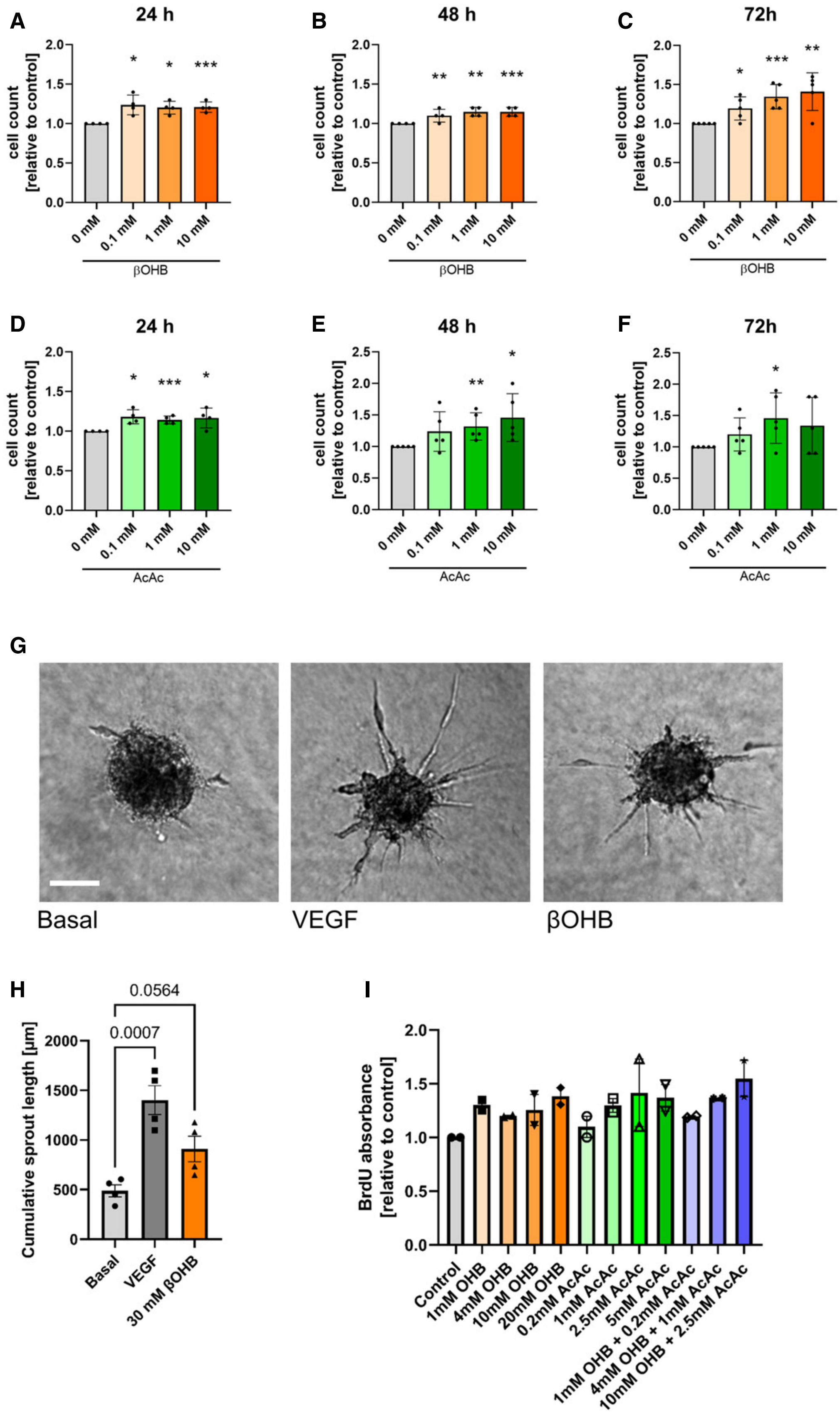

Figure EV2.

 © 2022 The Authors

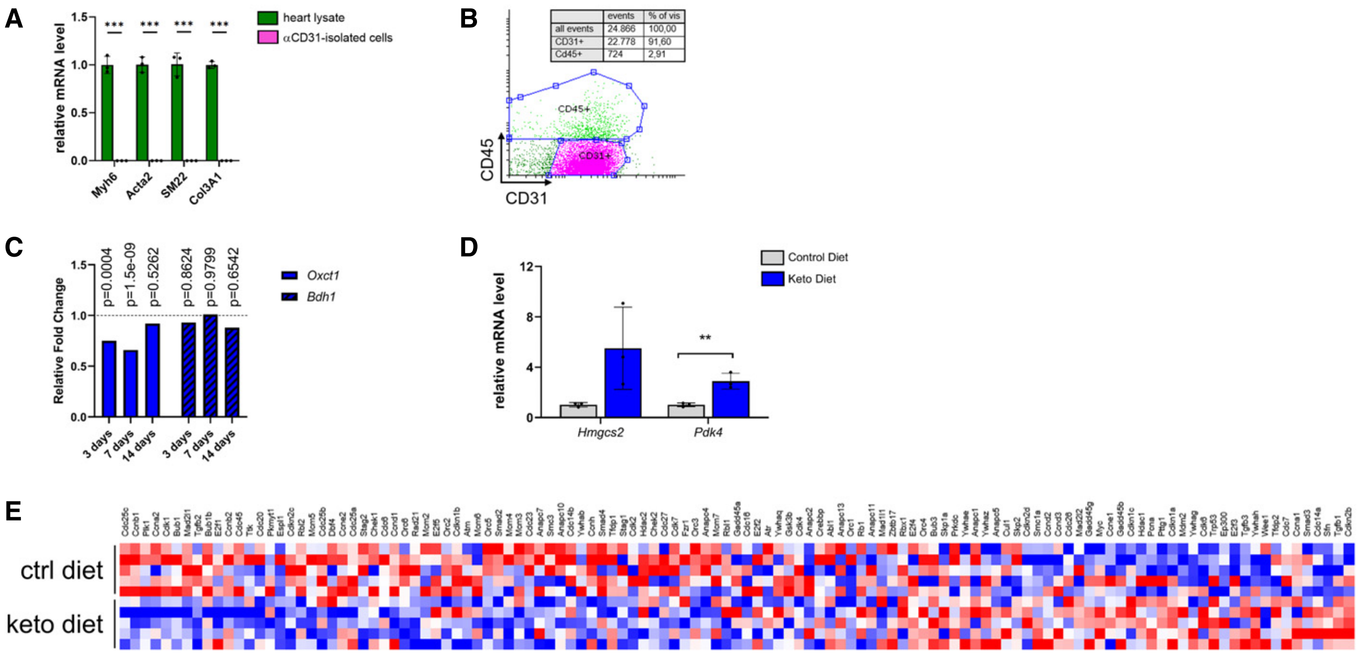

Figure EV3. Cardiac endothelial cells isolated from mice fed a ketogenic diet.

A Relative mRNA levels of cardiomyocyte, fibroblast, and smooth muscle cell marker genes in anti-CD31-isolated cells compared to levels in total heart lysates of C57Bl/6J mice.

B Flow cytometry analysis of anti-CD31-isolated cells from hearts of C57Bl/6J mice using the endothelial cell marker CD31 and the immune cell marker CD45.

C Relative fold changes of *Oxct1* (Scot) and *Bdh1* expression levels in mice fed a ketogenic diet relative to a control diet were obtained from RNAseq analyses.

D Relative mRNA levels of cardiac ECs isolated from mice fed a ketogenic or control diet confirming increased levels of pro-proliferative genes *Hmgcs2* and *Pdk4*.

E Heat-map showing expression levels of genes involved in regulation of cell cycle (KEGG). Data are presented as mean ± SD. $n \geq 3$. Two-tailed unpaired Student's *t*-test; $^{**}P < 0.01$; $^{***}P < 0.001$.

Figure EV4. Endothelial cell apoptosis in hearts of mice receiving a ketogenic diet.

A, B Quantification of cleaved caspase 3^+/CD31$^+$ double-positive cells per high power field (HPF) in heart sections of C57Bl/6J mice kept on a control diet or a ketogenic for (A) 2 weeks or (B) 4 weeks.

C Representative images of heart sections of animals kept on the control or ketogenic diet for 4 weeks stained against CD31.

D–G Quantification of CD31-positive area per high power field (HPF) in heart sections of mice kept on control diet (ctrl diet) or ketogenic diet (keto diet) for 2 weeks / 4 weeks / 6 weeks / 4 months. Scale bar: 50 µm.

H Quantification of Ki67$^+$/CD31$^+$ double-positive cells per high power field (HPF) in B16F10 tumor sections of C57Bl/6J mice kept on a control diet or a ketogenic 10 days after tumor inoculation.

I Quantification of CD31$^+$ vessels per high power field (HPF) in B16F10 tumor sections of C57Bl/6J mice kept on a control diet or a ketogenic 10 days after tumor inoculation.

J Tumor volume of C57Bl/6J mice kept on a control diet or ketogenic diet. Data are presented as mean ± SD. $n \geq 3$; statistical significance determined using unpaired Student's *t*-test.

▶

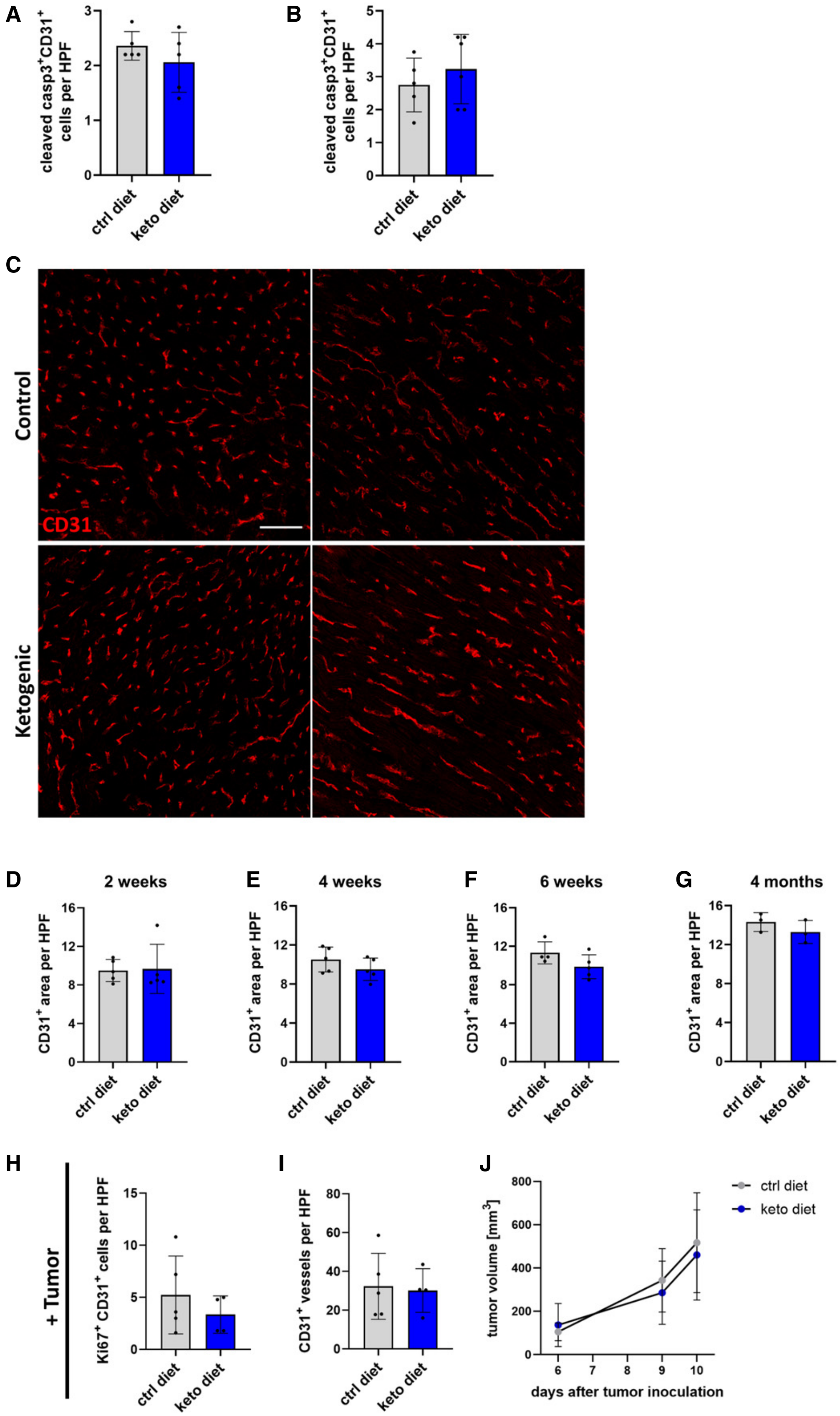

Figure EV4.

© 2022 The Authors

Figure EV5. Schematic overview of experimental animal groups for transverse aortic constriction study and aortic flow rates.

A Male and female C57Bl/6J mice were randomly assigned to a diet group at 8 weeks old. After 4 weeks on the respective diet, mice in each group underwent either transverse aortic constriction (TAC) or sham surgery. Mice were afterward kept on the respective diet for eight more weeks.

B Quantification of aortic blood flow rates within the stenosis of C57Bl/6J mice after TAC or sham surgery. Data are presented as mean $n \geq 2$.

C Trichrome staining of heart sections from sham and TAC-operated animals kept either on a control diet or ketogenic diet 8 weeks after surgery. Scale bar: 1 mm.

D Representative images of heart vasculature ($CD31^+$ vessels) of mice kept on a control or ketogenic diet 8 weeks after TAC surgery. Scale bar: 50 μm. Data are presented as mean ± SD. Two-tailed unpaired Student's *t*-test; $^{**}P < 0.01$; $^{***}P < 0.001$.

© 2022 The Authors

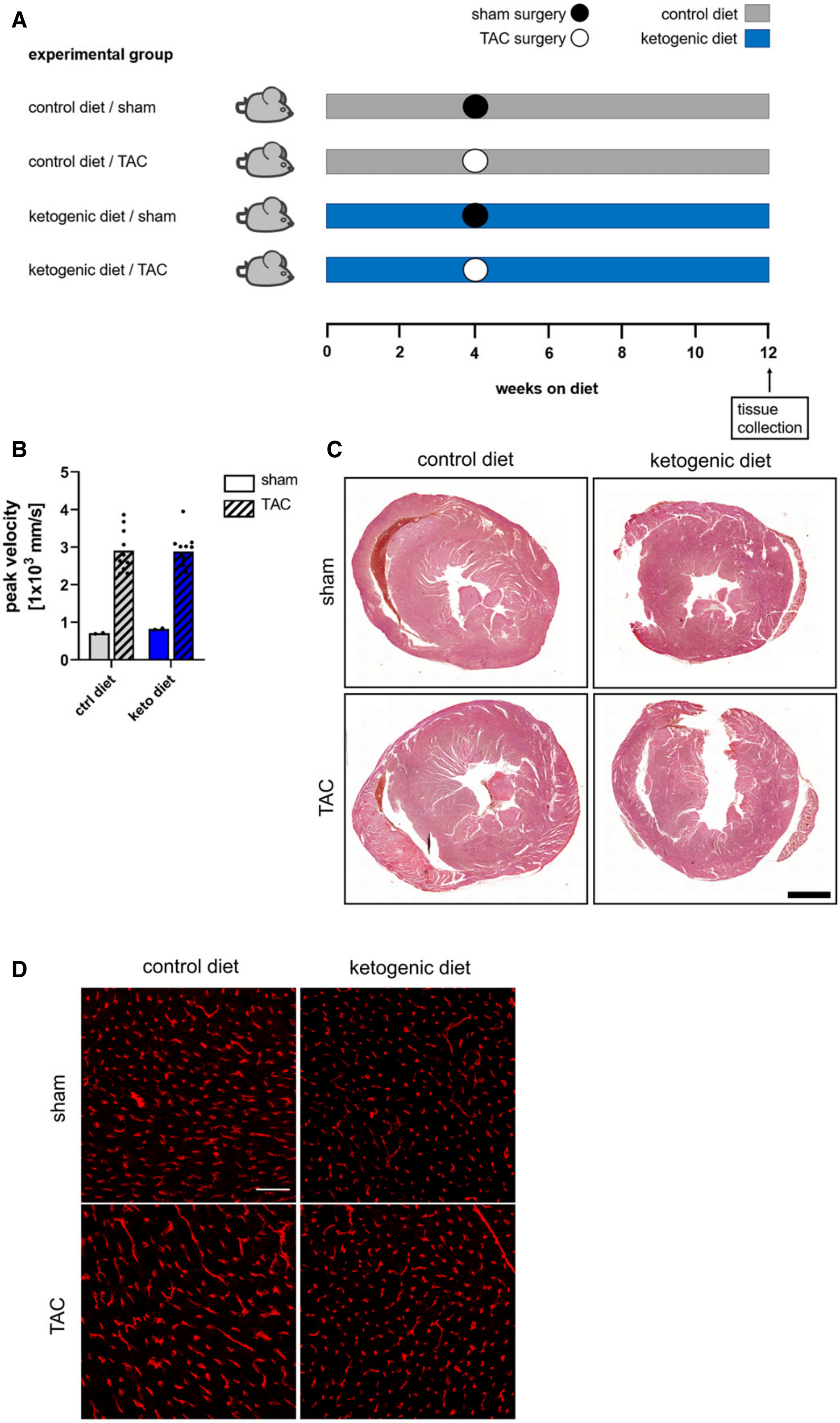

Figure EV5.

 © 2022 The Authors
