# Supplementary figures and images for: Ketone body oxidation increases cardiac endothelial cell proliferation
Source: EMBO Mol Med. 2022 Feb 18;14(4):e14753. doi: 10.15252/emmm.202114753 (PMC8988203; doi:10.15252/emmm.202114753)

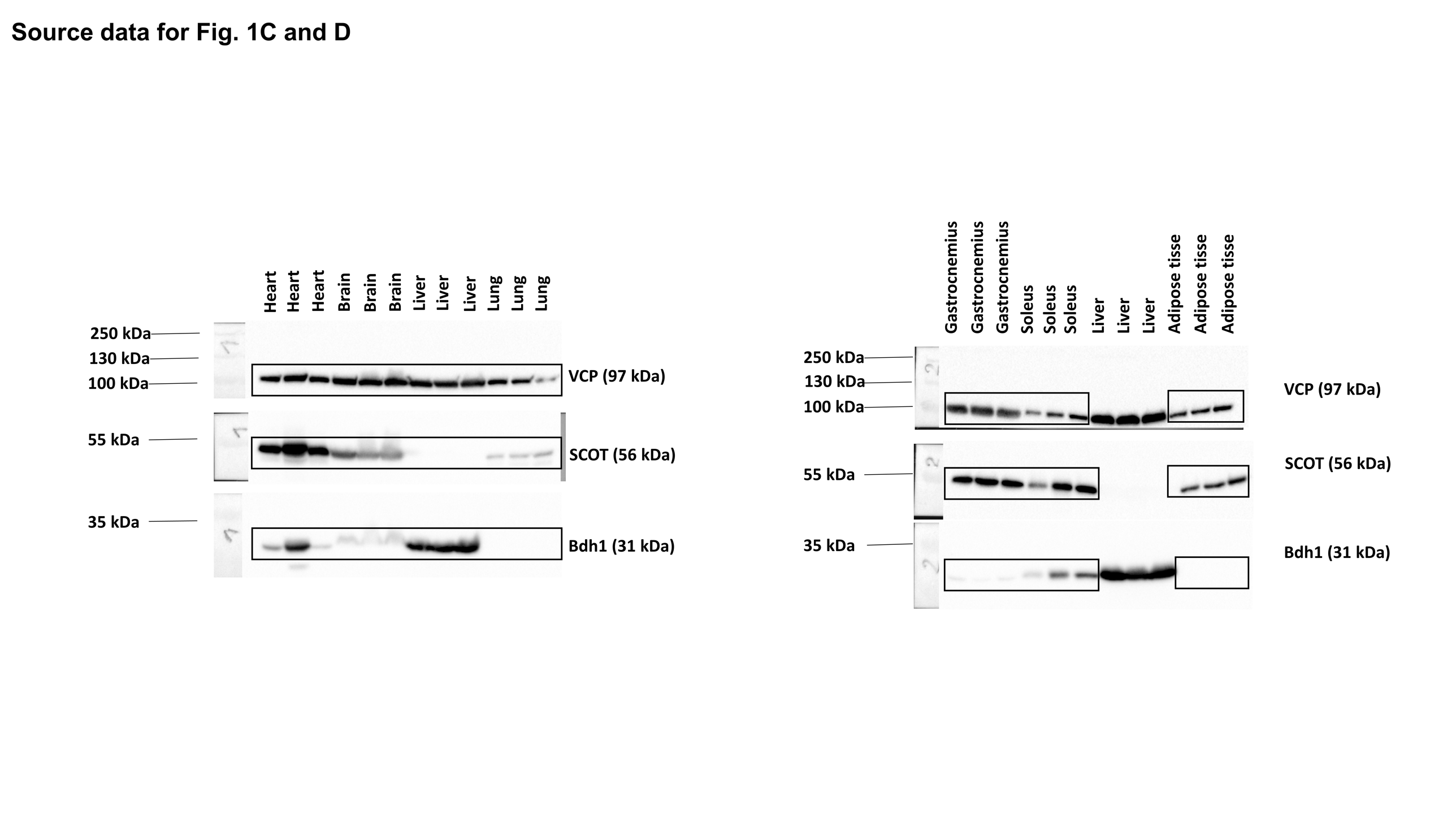

Supplement: Supplementary file 4 — Source Data for Figure 1 [file EMMM-14-e14753-s001.zip › EMM-2021-14753-V3-Figure_1C_1D_Source_Data-sd.tif]

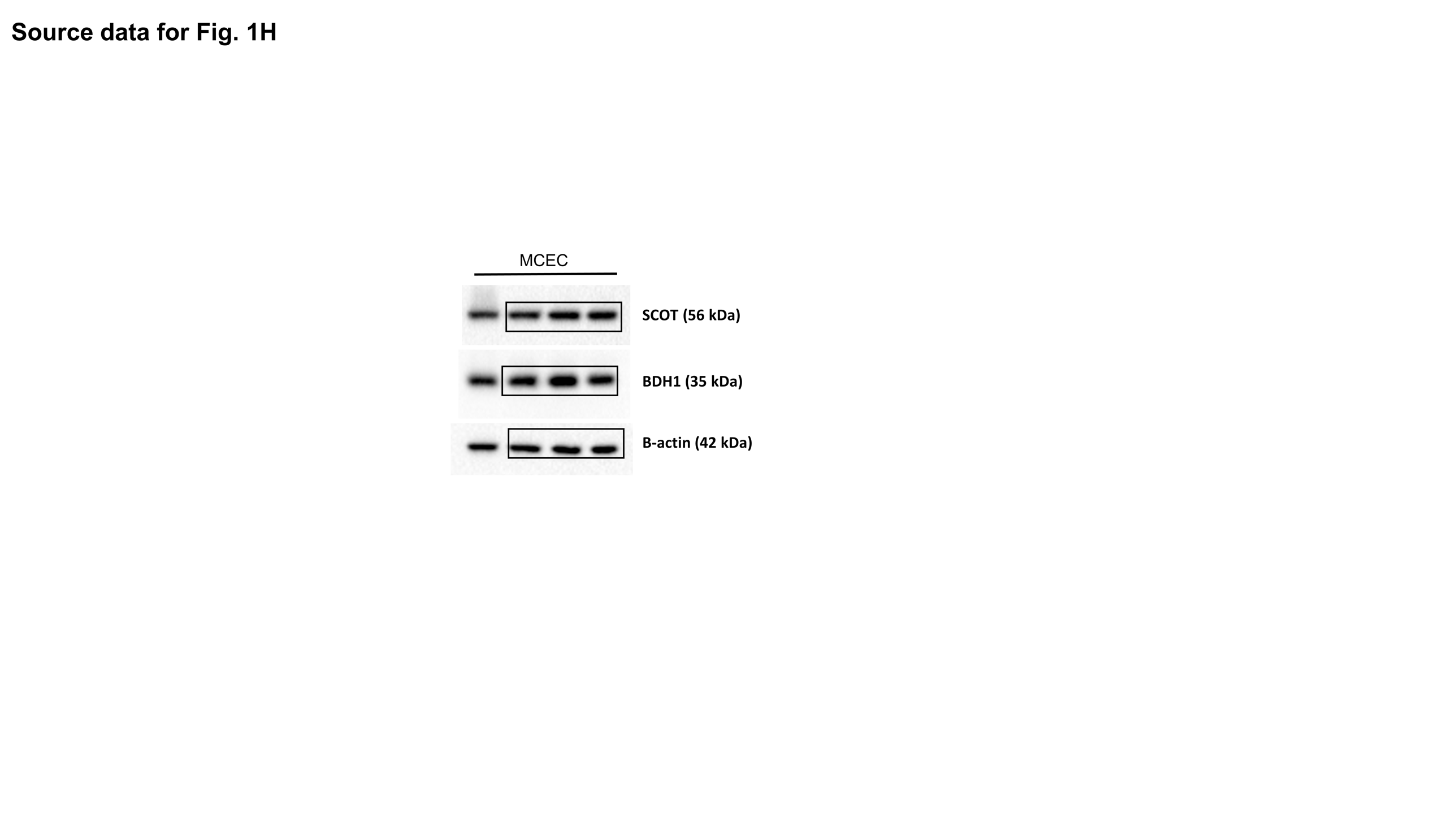

Supplement: Supplementary file 4 — Source Data for Figure 1 [file EMMM-14-e14753-s001.zip › EMM-2021-14753-V3-Figure_1H_Source_Data-sd.tif]

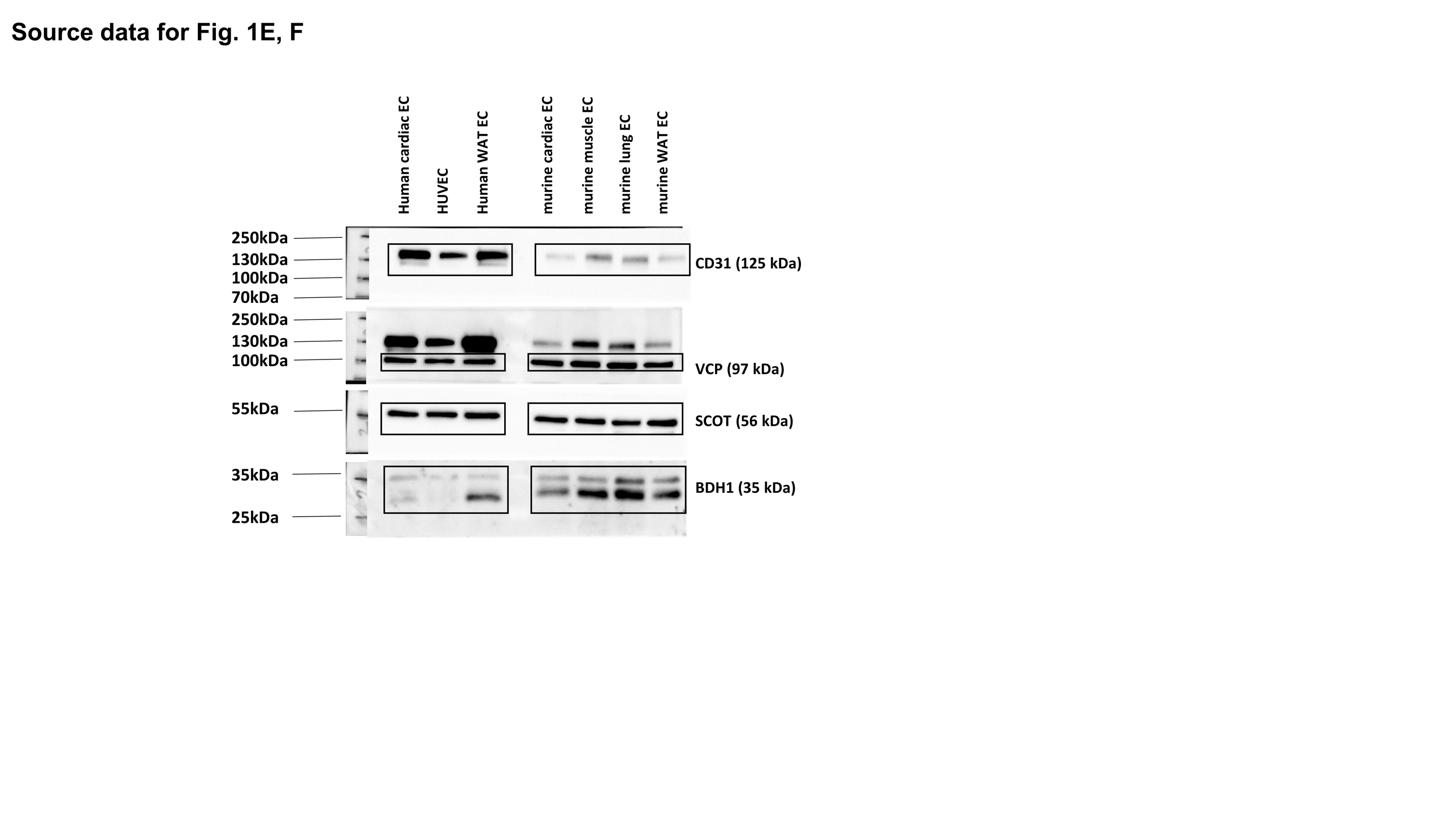

Supplement: Supplementary file 4 — Source Data for Figure 1 [file EMMM-14-e14753-s001.zip › EMM-2021-14753-V3-Figure_1E_1F_Source_Data-sd.tif]

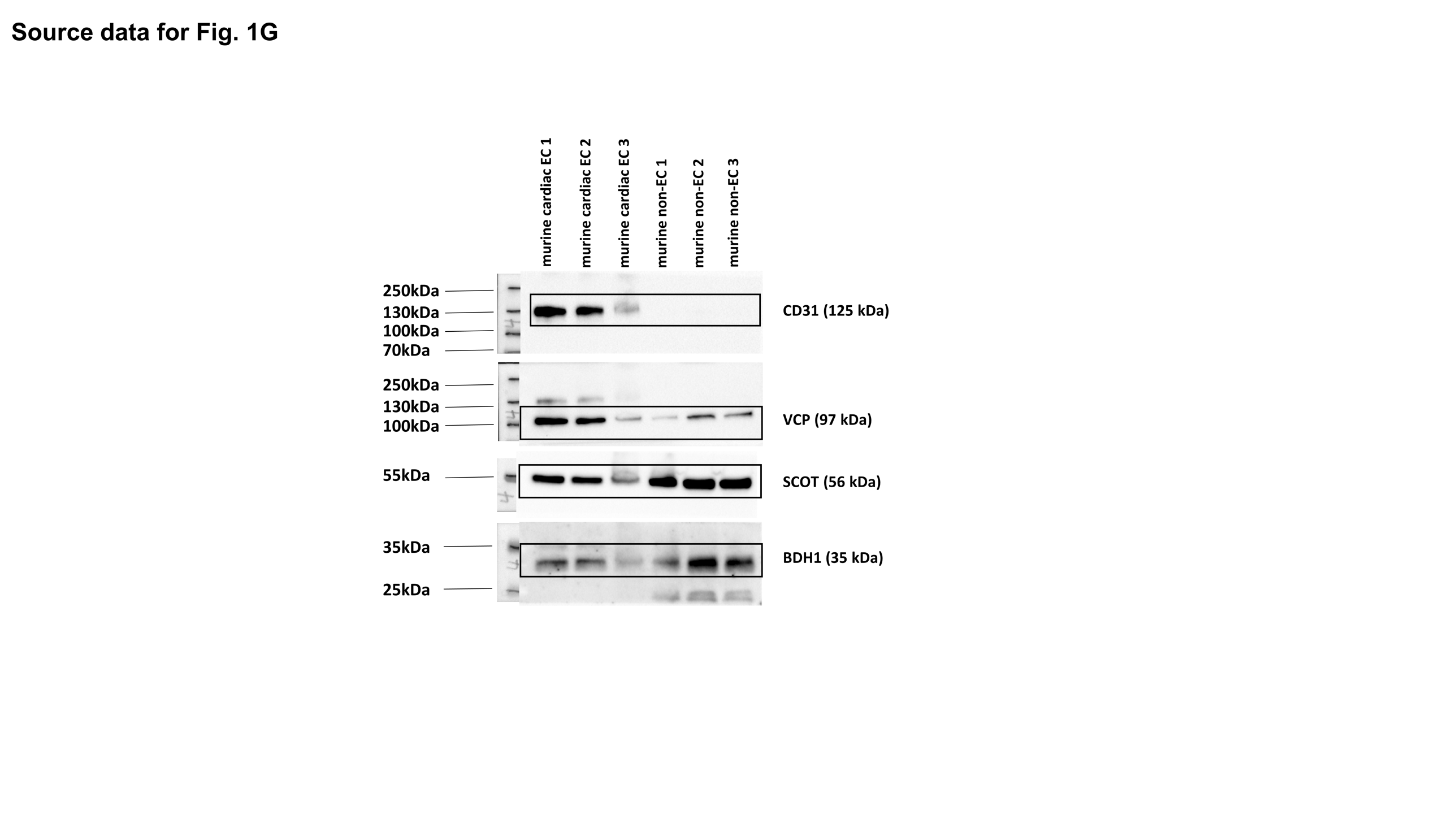

Supplement: Supplementary file 4 — Source Data for Figure 1 [file EMMM-14-e14753-s001.zip › EMM-2021-14753-V3-Figure_1G_Source_Data-sd.tif]

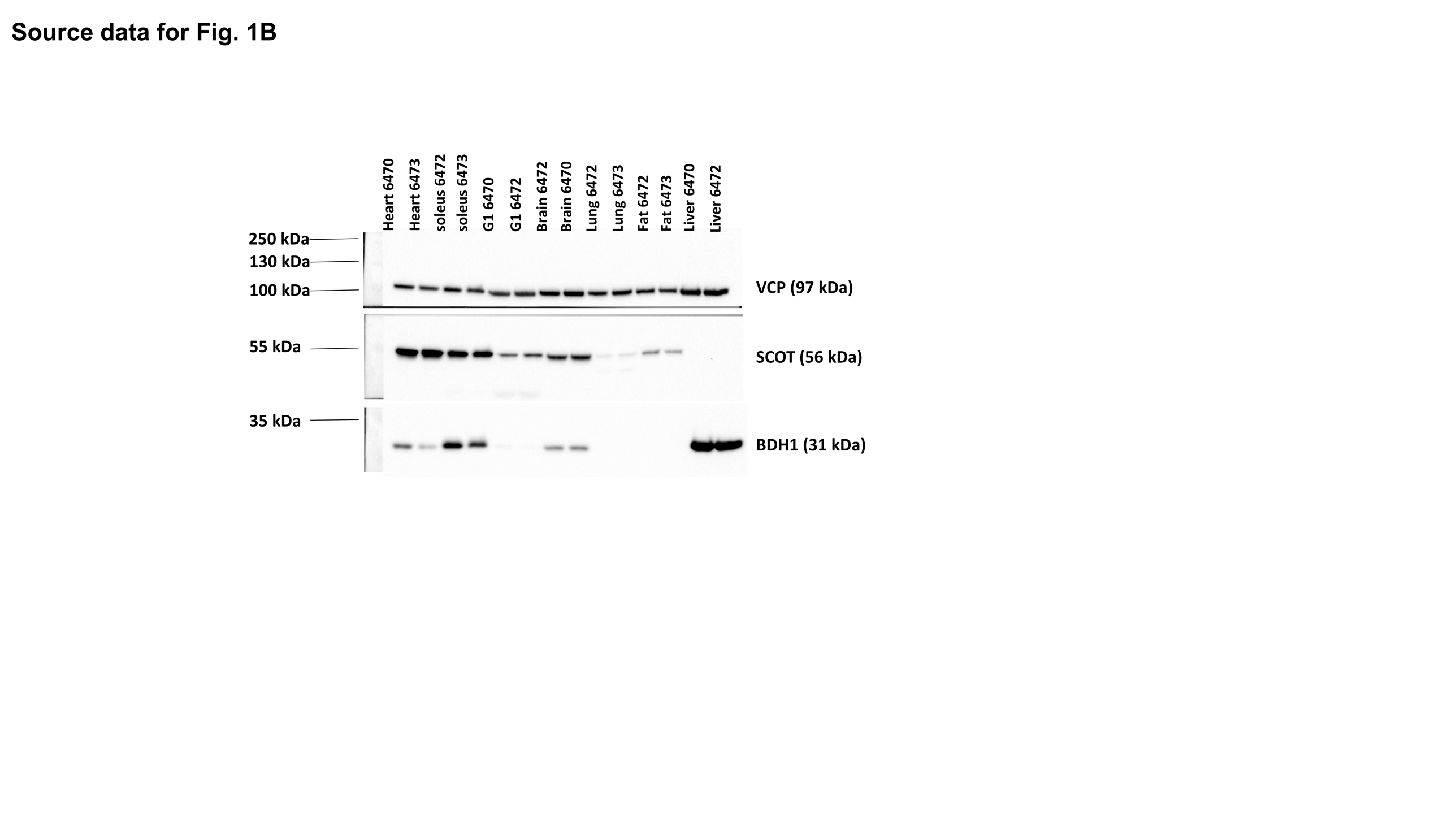

Supplement: Supplementary file 4 — Source Data for Figure 1 [file EMMM-14-e14753-s001.zip › EMM-2021-14753-V3-Figure_1B_Source_Data-sd.tif]

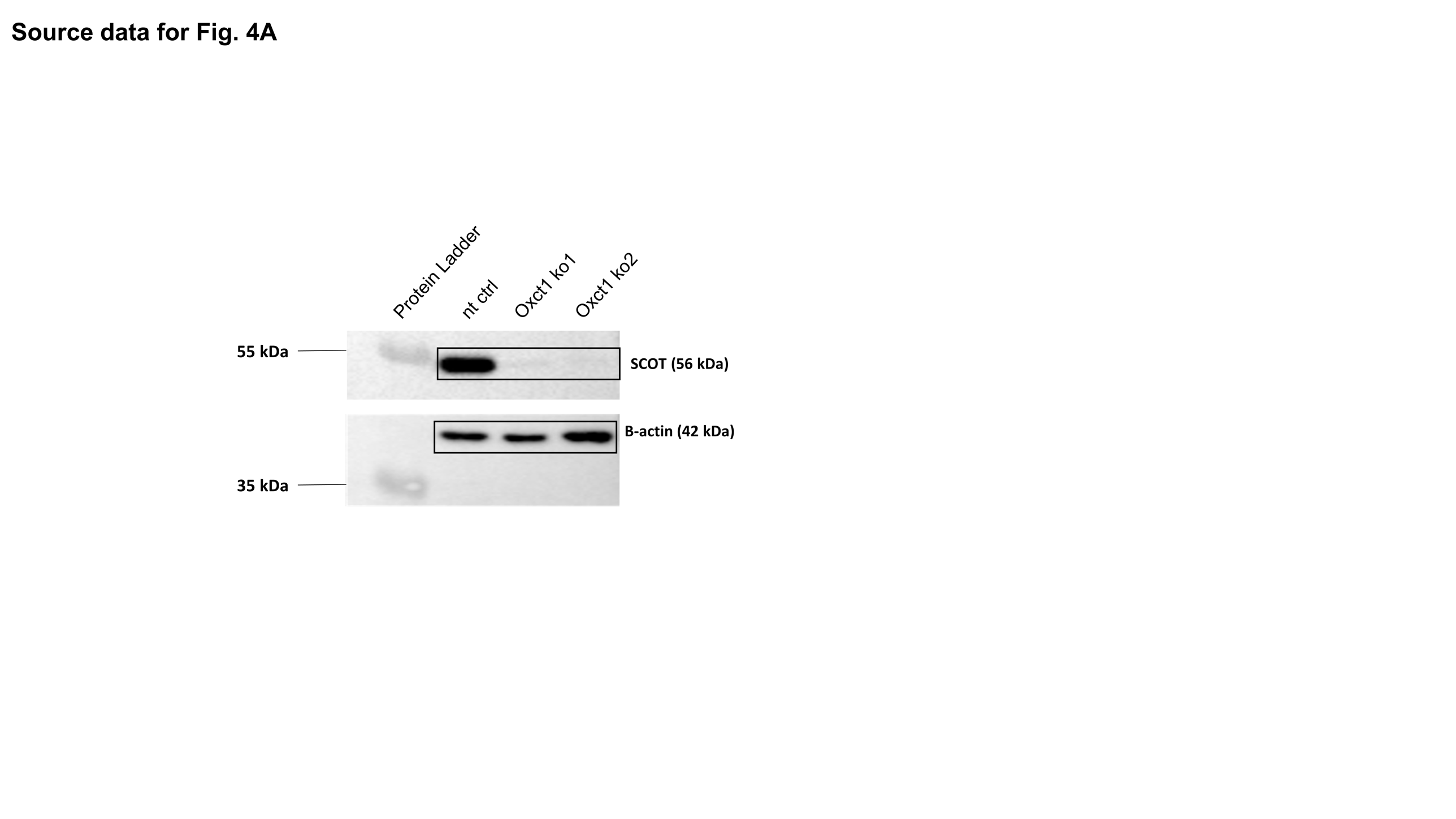

Supplement: Supplementary file 5 — Source Data for Figure 4A [file EMMM-14-e14753-s005.zip › emmm202114753-sup-0005-SDataFig4A.TIF]
