# Supplementary material for: Ketone body oxidation increases cardiac endothelial cell proliferation
Source: EMBO Mol Med. 2022 Feb 18;14(4):e14753. doi: 10.15252/emmm.202114753 (PMC8988203; doi:10.15252/emmm.202114753)
Supplement: Supplementary file 6 [file EMMM-14-e14753-s004.pdf]

EMBO Molecular Medicine

Ketone body oxidation increases cardiac endothelial cell proliferation

Eva-Maria Weis, Patrycja Puchalska, Alisa Nelson, Jacqueline Taylort, Iris Moll, Sana Hasan, Matthias Dewenter, Marco Hagenmueller, Thomas Fleming, Gernot Poschet, Agnes Hotz-Wagenblatt, Johannes Backs, Peter Crawford, and Andreas Fischer
DOI: 10.15252/emmm.202114753

Corresponding author: Andreas Fischer (a.fischer@dkfz.de)

Review Timeline:

Submission Date:	21st Jun 21
Editorial Decision:	20th Jul 21
Revision Received:	22nd Nov 21
Editorial Decision:	8th Dec 21
Revision Received:	20th Jan 22
Accepted:	24th Jan 22

Editor: Lise Roth

Transaction Report:

(Note: With the exception of the correction of typographical or spelling errors that could be a source of ambiguity, letters and reports are not edited. Depending on transfer agreements, referee reports obtained elsewhere may or may not be included in this compilation. Referee reports are anonymous unless the Referee chooses to sign their reports.)

20th Jul 2021

Dear Andreas,

Thank you for the submission of your manuscript to EMBO Molecular Medicine. We have now received feedback from the three reviewers who agreed to evaluate your manuscript. As you will see from the reports below, the referees acknowledge the interest of the study and are overall supporting publication of your work pending appropriate revisions.

Addressing the reviewers' concerns in full will be necessary for further considering the manuscript in our journal, and acceptance of the manuscript will entail a second round of review. EMBO Molecular Medicine encourages a single round of revision only and therefore, acceptance or rejection of the manuscript will depend on the completeness of your responses included in the next, final version of the manuscript. For this reason, and to save you from any frustrations in the end, I would strongly advise against returning an incomplete revision.

Revised manuscripts should be submitted within three months of a request for revision; they will otherwise be treated as new submissions, except under exceptional circumstances in which a short extension is obtained from the editor.

When submitting your revised manuscript, please carefully review the instructions that follow below. We perform an initial quality control of all revised manuscripts before re-review; failure to include requested items will delay the evaluation of your revision.

We require:

1) A .docx formatted version of the manuscript text (including legends for main figures, EV figures and tables). Please make sure that the changes are highlighted to be clearly visible.

2) Individual production quality figure files as .eps, .tif, .jpg (one file per figure). For guidance, download the 'Figure Guide PDF' (https://www.embopress.org/page/journal/17574684/authorguide#figureformat).

3) A .docx formatted letter INCLUDING the reviewers' reports and your detailed point-by-point responses to their comments. As part of the EMBO Press transparent editorial process, the point-by-point response is part of the Review Process File (RPF), which will be published alongside your paper.

4) A complete author checklist, which you can download from our author guidelines (https://www.embopress.org/page/journal/17574684/authorguide#submissionofrevisions). Please insert information in the checklist that is also reflected in the manuscript. The completed author checklist will also be part of the RPF.

5) It is mandatory to include a 'Data Availability' section after the Materials and Methods.
Note that the Data Availability Section is restricted to new primary datasets that are part of this study.

6) For data quantification: please specify the name of the statistical test used to generate error bars and P values, the number (n) of independent experiments (specify technical or biological replicates) underlying each data point and the test used to calculate p-values in each figure legend. The figure legends should contain a basic description of n, P and the test applied. Graphs must include a description of the bars and the error bars (s.d., s.e.m.).

7) We would also encourage you to include the source data for figure panels that show essential data. Numerical data should be provided as individual .xls or .csv files (including a tab describing the data). For blots or microscopy, uncropped images should be submitted (using a zip archive if multiple images need to be supplied for one panel). Additional information on source data and instruction on how to label the files are available at
.

8) Our journal encourages inclusion of *data citations in the reference list* to directly cite datasets that were re-used and obtained from public databases. Data citations in the article text are distinct from normal bibliographical citations and should directly link to the database records from which the data can be accessed. In the main text, data citations are formatted as follows: "Data ref: Smith et al, 2001" or "Data ref: NCBI Sequence Read Archive PRJNA342805, 2017". In the Reference list, data citations must be labeled with "[DATASET]". A data reference must provide the database name, accession number/identifiers and a resolvable link to the landing page from which the data can be accessed at the end of the reference. Further instructions are available at .

9) We replaced Supplementary Information with Expanded View (EV) Figures and Tables that are collapsible/expandable online. A maximum of 5 EV Figures can be typeset. EV Figures should be cited as 'Figure EV1, Figure EV2" etc... in the text and their

respective legends should be included in the main text after the legends of regular figures.
- For the figures that you do NOT wish to display as Expanded View figures, they should be bundled together with their legends in a single PDF file called *Appendix*, which should start with a short Table of Content. Appendix figures should be referred to in the main text as: "Appendix Figure S1, Appendix Figure S2" etc.
- Additional Tables/Datasets should be labeled and referred to as Table EV1, Dataset EV1, etc. Legends have to be provided in a separate tab in case of .xls files. Alternatively, the legend can be supplied as a separate text file (README) and zipped together with the Table/Dataset file.

See detailed instructions here:
.

10) The paper explained: EMBO Molecular Medicine articles are accompanied by a summary of the articles to emphasize the major findings in the paper and their medical implications for the non-specialist reader. Please provide a draft summary of your article highlighting
- the medical issue you are addressing,
- the results obtained and
- their clinical impact.
This may be edited to ensure that readers understand the significance and context of the research. Please refer to any of our published articles for an example.

11) For more information: There is space at the end of each article to list relevant web links for further consultation by our readers. Could you identify some relevant ones and provide such information as well? Some examples are patient associations, relevant databases, OMIM/proteins/genes links, author's websites, etc...

12) Every published paper now includes a 'Synopsis' to further enhance discoverability. Synopses are displayed on the journal webpage and are freely accessible to all readers. They include a short stand first (maximum of 300 characters, including space) as well as 2-5 one-sentences bullet points that summarizes the paper. Please write the bullet points to summarize the key NEW findings. They should be designed to be complementary to the abstract - i.e. not repeat the same text. We encourage inclusion of key acronyms and quantitative information (maximum of 30 words / bullet point). Please use the passive voice. Please attach these in a separate file or send them by email, we will incorporate them accordingly.

Please also suggest a striking image or visual abstract to illustrate your article as a PNG file 550 px wide x 300-600 px high.

13) As part of the EMBO Publications transparent editorial process initiative (see our Editorial at http://embomolmed.embopress.org/content/2/9/329), EMBO Molecular Medicine will publish online a Review Process File (RPF) to accompany accepted manuscripts.
In the event of acceptance, this file will be published in conjunction with your paper and will include the anonymous referee reports, your point-by-point response and all pertinent correspondence relating to the manuscript. Let us know whether you agree with the publication of the RPF and as here, if you want to remove or not any figures from it prior to publication.
Please note that the Authors checklist will be published at the end of the RPF.

EMBO Molecular Medicine has a "scooping protection" policy, whereby similar findings that are published by others during review or revision are not a criterion for rejection. Should you decide to submit a revised version, I do ask that you get in touch after three months if you have not completed it, to update us on the status.

I look forward to receiving your revised manuscript.

With my best wishes,

Lise

Lise Roth, PhD
Editor
EMBO Molecular Medicine

***** Reviewer's comments *****

Referee #1 (Comments on Novelty/Model System for Author):

Models are adequate
The study is novel, and it provides new insights in the metabolic control of EC function in an intratissue manner.

Referee #1 (Remarks for Author):

In this study, Weis et al. provide evidences that cardiac ECs oxidize ketone bodies which are then used for these cells to proliferate. Also, they demonstrate that cardiac ECs express the key enzymes required for their metabolization. Upon feeding mice with high-fat, low-carbohydrate ketogenic diet, cardiac ECs transiently proliferate in vivo which prevents vessel rarefication in a mouse model of heart hypertrophy.

The study is well designed, and the data provided support the main conclusions. However, the authors need to clarify several points as described below. My major comment is that authors should address why and when cardiac ECs use ketone bodies for oxidative purposes? This reviewer wonders whether it is simply an endothelial-related protective mechanism under physiological ketosis.

-Does ketone bodies oxidation in ECs result in an increase in ATP levels?

-Cardiac ECs express SCOT and BDH1 (both required to oxidase ketone bodies). The key question is whether this is unique of cardiac ECs, or all EC from any vascular bed express these enzymes.

-Authors demonstrate that supplementation of ketone bodies stimulates angiogenesis in vitro. However, this does not seem to be the case in vivo. Do the authors have an explanation for that?

-Are ketone bodies a regular source of energy of cardiac ECs, or this only occurs under specific pathophysiological conditions?

-Author claim that ketone bodies stimulate EC migration, but no data supporting this are provided.

- If Edu is administered orally starting from the day of switching diet, how do authors explain that after 6 weeks no Edu positive cells are detected?

-How do authors explain that there is an increase in ECs proliferation without changes in vessel density (in vivo)? Is it possible that the effect on ECs on proliferation is not an angiogenic response but instead a protective response to avoid vascular rarefication under pathophysiological ketosis.

Referee #2 (Comments on Novelty/Model System for Author):

My overall impression is that the manuscript looks relatively solid. The findings are not striking or unexpected but still potentially relevant. The reason why I am saying "potentially" is that several experimental approaches need further experimental detail or validation tp provide definitive insight.

The Carmeliet laboratory already published a paper on ketone bodies in lymphatic endothelial cells, which reported similar findings in these cells; however, this is not a major issue from my point of view. Blood and lymphatic endothelial differ in many ways, so reporting the similarities and differences is important for investigators in the field.

If appropriately revised, a short report in EMBO Molecular Medicine could be a suitable format for this paper.

Referee #2 (Remarks for Author):

The manuscript by Weis and colleagues describes a pathophysiological role of ketone bodies in the blood vasculature. Overall, the work looks relatively solid. The findings are not striking or unexpected but still potentially relevant. I have the following comments for the authors, which they want to address in a revised version of the manuscript.

Specific comments:

Figure 1B and related: The molecular weight markers should label the protein properly. As shown, it is unclear where the kDa

label is placed.

Figure 1E, F: Endothelial markers need to be shown to demonstrate the proposed cellular fraction is indeed endothelial in nature.

The authors should use labeled ketone bodies to confirm their oxidation in ECs.

Do other endothelial cells also break down ketone bodies, or is this specific for cardiac endothelial cells?

The authors state that endothelial cells use ketone bodies to generate biomass; however, this statement is just based on the finding the 13C label is found in lipids and amino acids. A more detailed analysis is needed to clarify whether these metabolites are used for biosynthesis or energy generation. And what about mitochondrial respiration? Is respiration increased in D-ß-hydroxybutyrate- or acetoacetate-stimulated cells?

Figure 3A-D: From the data shown, it is not completely clear whether cell growth, proliferation, or both are affected. Simply counting the cells would provide insight into the response to ketone bodies.

Figure 3G-J: These figure panels show a lot of processed data, making it impossible to assess the data. Representative images of the angiogenic assays should be delivered.

If the cellular effects of D-ß-hydroxybutyrate and acetoacetate rely on their oxidation in the TCA cycle, why then do other TCA cycle substrate not promote angiogenesis? Any explanations?

The gene expression changes in cardiac endothelial cells in response to the ketogenic diet are minute. In light of these subtle changes, it is essential to validate some of the described targets (e.g., PDK4).

Figure 5E: Which cell cycle genes are regulated by the ketogenic diet and in which direction. The enrichment analysis per se does not provide insight into whether the proliferation is increased. Genes that cause a cell cycle arrest could also drive the relative enrichment in this category.

The GSEA analyses in Figure 5F, G looks striking. However, given that overall gene expression are modest, one wonders how these gene set analysis results were obtained. Was the entire transcriptome taken into consideration or only a subset of genes? This should be explained in the Methods part of the paper.

Figure 6A, B.: The tissue sections should be labeled for a pan-endothelial marker to illustrate the tissue architecture. Along these lines, is the microvascular density altered in mice on a ketogenic diet? In which part of the cardiac muscle was the analysis performed? Left or right ventricle?

Figure 7: Again, original data is scarce in this figure. Representative images of the heart and its vasculature need to be shown for different treatment groups.

Referee #3 (Remarks for Author):

The present interesting manuscript is well written and structured. In this manuscript the authors present novel data regarding the metabolism of cardiac endothelial cells. Specifically, the authors found that cardiac endothelial cells are capable of oxidizing ketone bodies and that ketone bodies might promote cardiac endothelial cell proliferation, migration and angiogenic sprouting. Moreover, ketogenic diet is transiently increasing cardiac endothelial cell proliferation in vivo. In addition, in a model of pressure overload-induced cardiac hypertrophy ketogenic diet maintained vessel density. The data are interesting and novel and provide insight on the metabolism of cardiac endothelial cells. However, the data are incomplete in the present form and there are some additional issues, which have to be experimentally addressed by the authors.

Major points:
1
a) I suggest to the authors to comparatively study the expression of SCOT and BDH1 in murine and human endothelial cells from different tissues.

b) How is the expression of SCOT and BDH1 regulated in endothelial cells ?
- Is starvation in vitro and ketogenic diet in vivo affecting the expression of SCOT and BDH1 expression in endothelial cells from heart and other tissues ?
- How do angiogenic growth factors, inflammatory cytokines and hypoxia affect the expression of SCOT and BDH1 in cardiac and non-cardiac endothelial cells ?

#2
Which is the effect of ketone bodies on murine and human non-cardiac endothelial cells regarding proliferation, migration and angiogenic sprouting ?

#3
Which is the molecular explanation for the proposed differential response of cardiac endothelial cells to ketogenic diet in comparison to non-cardiac endothelial cells in regard to proliferation ? Are cardiomyocyte-derived factors involved in this differential response of cardiac endothelial cells ?

#4
The authors demonstrated that ketogenic diet over a longer period (for six weeks) did not result in increased proliferation of cardiac endothelial cells. Therefore, I suggest to the authors to study the effects of a ketogenic diet over 6 weeks on the expression of SCOT and BDH1 in endothelial cells isolated from mice hearts.

Weis et al., Ketone body oxidation increases cardiac endothelial cell proliferation.

Referee #1 (Comments on Novelty/Model System for Author):
Models are adequate
The study is novel, and it provides new insights in the metabolic control of EC function in an intratissue manner.

(Remarks for Author):

In this study, Weis et al. provide evidences that cardiac ECs oxidize ketone bodies which are then used for these cells to proliferate. Also, they demonstrate that cardiac ECs express the key enzymes required for their metabolization. Upon feeding mice with high-fat, low-carbohydrate ketogenic diet, cardiac ECs transiently proliferate in vivo which prevents vessel rarefication in a mouse model of heart hypertrophy.

The study is well designed, and the data provided support the main conclusions. However, the authors need to clarify several points as described below. My major comment is that authors should address why and when cardiac ECs use ketone bodies for oxidative purposes? This reviewer wonders whether it is simply an endothelial-related protective mechanism under physiological ketosis.

We thank the reviewer for her/his very positive assessment of our manuscript.

-Does ketone bodies oxidation in ECs result in an increase in ATP levels?
This is an interesting question. We cultured ECs for Seahorse analysis and we observed that ketone bodies could fuel the mitochondrial electron transfer chain (Fig. EV1) under serum starved conditions. The presence of ketone bodies in the culture medium led to increased maximal mitochondrial respiration and ATP production (Fig. EV1D and F). We did not expect higher basal ATP levels as ATP cannot be stored properly. Indeed, measuring ATP by a luciferase assay as well as by UPLC revealed that ATP levels were not changed upon adding ketone bodies to the culture medium (Fig. EV1I and J). We also measured AMP, ADP and several other nucleotides. All of these were not changed after adding ketone bodies to the culture medium (Fig. EV1K and L). In summary, the data suggest that ketone bodies can be an alternative source for the generation of biomass (Fig. 2) and ATP in ECs.

-Cardiac ECs express SCOT and BDH1 (both required to oxidase ketone bodies). The key question is whether this is unique of cardiac ECs, or all EC from any vascular bed express these enzymes.
This is an outstanding question which was also raised by the other two referees. To address this, we performed several experiments. First, we isolated primary ECs from murine adipose tissue, skeletal muscle and cardiac muscle. We also used primary human umbilical vein ECs (HUVEC), adipose tissue ECs and cardiac microvascular ECs. All of these ECs expressed SCOT and BDH1 (Fig. 1F). Secondly, we analyzed the single cell RNA sequencing atlas of endothelial cells from eleven mouse organs (published by Peter Carmeliet' group in *Cell* 2020;180(4):764-779.e20. PMID: 32059779). BDH1 was not detectable in this atlas, which might be due to the fact that droplet-based sequencing was employed. The sequencing depth is not as high as with SMARTseq protocols therefore transcripts with low expression

strength are often not detected. There was however SCOT expression in ECs of all eleven organs. In the heart, SCOT expression was detected in all EC types such as arterial, venous, capillary (Appendix Fig. S1). Thirdly, we tested whether ketone bodies promote sprouting angiogenesis also in human umbilical vein endothelial cells (HUVEC) and indeed this was the case (Fig. EV2). In summary, the data suggest that ketone body oxidation in not unique to cardiac ECs. However, in vivo ketogenic diet only promoted EC proliferation in the heart (Fig 6F). This is why we focused the paper on cardiac ECs.

-Authors demonstrate that supplementation of ketone bodies stimulates angiogenesis in vitro. However, this does not seem to be the case in vivo. Do the authors have an explanation for that?

We do not have a concrete answer for this question. However, one probable explanation could be the role of organ-derived factors on ECs. In this case, cardiomyocytes-derived factors could drive differential response in cardiac ECs compared to other tissues. Another reason could be that it is very difficult to stimulate angiogenesis in an adult mouse. Multiple trials failed to e.g. stimulate cardiac angiogenesis after heart infarction by the application of potent proangiogenic growth factors like VEGF. However, also in the tumor model which requires angiogenesis for rapid tumor growth we could not see an effect by ketogenic diet (Fig. EV4 H- J). However, in this context there are already numerous pro-angiogenic factors present that addition of another one probably makes no difference. In summary, we think that the local microenvironment prevents vessel sprouting in organs of the adult mouse which can only be overcome by a strong stimulus such as wounding. However, when using such models, there is already such a high amount of pro-angiogenic factors present that the addition of a single metabolite has no further measurable effect. We speculate that ketone bodies might be helpful in preventing vessel regression, e.g. the data of the heart hypertrophy model (Fig. 7D) indicates this, however, more sophisticated studies will be needed to clarify this in the future.

-Are ketone bodies a regular source of energy of cardiac ECs, or this only occurs under specific pathophysiological conditions?

This of course is an important question to be addressed in future studies. We are not aware of any publication showing that ECs can oxidize ketone bodies at all. This will be the first paper describing it.

Our new data using Seahorse technology to measure mitochondrial respiration show that only upon starvation the addition of ketone bodies to the culture medium increases ATP production. This was not observed under culture conditions in which other nutrients are available (Fig. EV1). As such, one could speculate that ketone bodies only become an important source for energy in circumstances in which other nutrients are limited.

-Author claim that ketone bodies stimulate EC migration, but no data supporting this are provided.

The data showing EC migration are shown in Fig. 3E and F.

- If Edu is administered orally starting from the day of switching diet, how do authors explain that after 6 weeks no Edu positive cells are detected?

We apologize for not having written this clear enough. There are indeed Edu-positive cells after 6 weeks (and also at the other time points) at all conditions. What we wanted to say is that there were no significant differences in the number of EdU-positive ECs between the

two groups (ketogenic vs. control). The text in the results section was changed accordingly. Please also refer to Fig. 6A-D.

-How do authors explain that there is an increase in ECs proliferation without changes in vessel density (in vivo)? Is it possible that the effect on ECs on proliferation is not an angiogenic response but instead a protective response to avoid vascular rarefication under pathophysiological ketosis.

As already mentioned, it is very difficult to increase vessel density in an adult organism such as mice by simply adding vascular growth factors. The landmark study by the Eli Keshet group published in *Science* a few weeks ago showed however that it is possible to prevent vessel rarefication during ageing. As such, it could very well be that oxidation of ketone bodies might help to prevent vessel rarefication (e.g. in the heart hypertrophy experiment Fig. 7D). However, this needs further investigation. Based on this reviewer's very valuable comment we added this idea to the last sentence of the Discussion.

Referee #2 (Remarks for Author):

My overall impression is that the manuscript looks relatively solid. The findings are not striking or unexpected but still potentially relevant. The reason why I am saying "potentially" is that several experimental approaches need further experimental detail or validation tp provide definitive insight.

The Carmeliet laboratory already published a paper on ketone bodies in lymphatic endothelial cells, which reported similar findings in these cells; however, this is not a major issue from my point of view. Blood and lymphatic endothelial differ in many ways, so reporting the similarities and differences is important for investigators in the field.

If appropriately revised, a short report in EMBO Molecular Medicine could be a suitable format for this paper.

Remarks for Author:

The manuscript by Weis and colleagues describes a pathophysiological role of ketone bodies in the blood vasculature. Overall, the work looks relatively solid. The findings are not striking or unexpected but still potentially relevant. I have the following comments for the authors, which they want to address in a revised version of the manuscript.

We thank the reviewer for her/his very positive judgement of our manuscript.

Figure 1B and related: The molecular weight markers should label the protein properly. As shown, it is unclear where the kDa label is placed.

We added the kDa label for the expected size of the protein. In the revised version, we have also uploaded the original uncropped Western blots including the molecular size markers (Source data file).

The authors should use labeled ketone bodies to confirm their oxidation in ECs.

The authors state that endothelial cells use ketone bodies to generate biomass; however, this statement is just based on the finding the 13C label is found in lipids and amino acids. A more detailed analysis is needed to clarify whether these metabolites are used for biosynthesis or energy generation.

If the cellular effects of D-ß-hydroxybutyrate and acetoacetate rely on their oxidation in the TCA cycle, why then do other TCA cycle substrate not promote angiogenesis? Any explanations?

These comments, separated as different thoughts in the reviewer comments, are addressed together.
Our metabolite labeling and total metabolite pool data in Fig. 2 support the notion that ketone bodies contribute to numerous oxidative and anabolic pathways. The relatively greater penetration of labeling of most metabolite pools from [13C]AcAc, compared to [13C]BOHB, suggests the canonical procession of BOHB through a BDH1-dependent step before AcAc, which then proceeds toward acetyl-CoA in mitochondria (via SCOT) or the cytoplasm (via AACS; https://pubmed.ncbi.nlm.nih.gov/28178565/). That both BOHB and AcAc have a comparable impact on MCEC proliferation, migration, and sprouting (Fig. 3) supports the notion that mitochondrial ketone metabolism is required for these effects, and not cytoplasmic AACS-dependent effects. Moreover, that L-BOHB does not support sprouting also underscores the requirement for mitochondrial metabolism, because L-BOHB is not oxidized like D-BOHB, because L-BOHB is not a substrate for mitochondrial BDH1, which oxidizes D-BOHB to AcAc while reducing NAD+ to NADH.

Our observations are entirely consistent with the previous findings that lymphatic endothelial cell (LEC) proliferation, migration and vessel sprouting are impaired in the absence of mitochondrial SCOT (https://www.nature.com/articles/s42255-019-0087-y). As we observed in MCECs, TCA cycle intermediate pools were augmented by ketone bodies in LECs. This concordance is together supportive of the notion that mitochondrial conversion of ketone bodies to acetyl-CoA is required to provoke these phenotypes.

Measuring the impact of ketone bodies on mitochondrial respiration is of interest (and we did perform this experiment *see below*, but irrespective of the outcome of this conceptually interesting experiment (i.e., whether ketones increase cellular JO2 or not), this experiment will not address how procession of ketone body carbon through mitochondrial acetyl-CoA, into the TCA cycle, and subsequent cataplerotic efflux of these intermediates out into supporting amino acid and lipid production is a concordant theme for ketone bodies in MCECs (our study), LECs (https://www.nature.com/articles/s42255-019-0087-y), and tissue macrophages (https://www.cell.com/cell-metabolism/fulltext/S1550-4131(18)30647-8). In each of these studies, ketone bodies, and not other substrates, were uniquely required to drive the observed phenotypes. Indeed the reason why ketone body-derived acetyl-CoA, and not fat or glucose-derived acetyl-CoA, is able to provoke the observed phenotypes is not fully clear. Ketone bodies quickly elevate mitochondrial [acetyl-CoA], a characteristic that is shared with fatty acids, but not glucose. However, conversion of

ketones to acetyl-CoA also inhibits the contribution of fatty-acid-derived acetyl-CoA to the TCA cycle (https://pubmed.ncbi.nlm.nih.gov/25353003/, https://pubmed.ncbi.nlm.nih.gov/30668551/). Thus, among the effects of the contribution of ketones to the TCA cycle is their metabolic competition with fat, with consequential metabolic, redox, and signaling effects, which again would not be reported by measuring JO2 alone (https://pubmed.ncbi.nlm.nih.gov/28178565/). Future studies that mechanistically dissect the unique ways in which mitochondrial ketone metabolism influences cellular processes that extend beyond mitochondrial energy homeostasis.

Do other endothelial cells also break down ketone bodies, or is this specific for cardiac endothelial cells?

This is an outstanding question which was also raised by the other two referees. To address this, we performed several experiments. First, we isolated primary ECs from murine adipose tissue, skeletal muscle and cardiac muscle. We also used primary human umbilical vein ECs (HUVEC), adipose tissue ECs and cardiac microvascular ECs. All of these ECs expressed SCOT and BDH1 (Fig. 1F). Secondly, we analyzed the single cell RNA sequencing atlas of endothelial cells from eleven mouse organs (published by Peter Carmeliet' group in *Cell* 2020;180(4):764-779.e20. PMID: 32059779). BDH1 was not detectable in this atlas, which might be due to the fact that droplet-based sequencing was employed. The sequencing depth is not as high as with SMARTseq protocols therefore transcripts with low expression strength are often not detected. There was however SCOT expression in ECs of all eleven organs. In the heart, SCOT expression was detected in all EC types such as arterial, venous, capillary (Appendix Fig. S1). Thirdly, we tested whether ketone bodies promote sprouting angiogenesis also in human umbilical vein endothelial cells (HUVEC) and indeed this was the case (Fig. EV2). In summary, the data suggest that ketone body oxidation in not unique to cardiac ECs. However, in vivo ketogenic diet only promoted EC proliferation in the heart (Fig. 6F). This is why we focused the paper on cardiac ECs.

And what about mitochondrial respiration? Is respiration increased in D-ß-hydroxybutyrate- or acetoacetate-stimulated cells?

As already mentioned in the response to reviewer #1, we did not expect higher basal ATP levels as ATP cannot be stored properly but of course it could well be that ECs use ketone bodies instead of glucose or fatty acids to generate ATP. Indeed, measuring ATP by a luciferase assay as well as by UPLC revealed that ATP levels were not changed upon adding ketone bodies to the culture medium (Fig. EV1I and J). We also measured AMP, ADP and several other nucleotides. All of these were not changed after adding ketone bodies to the culture medium (Fig. EV1K and L).

However, when we cultured ECs for Seahorse analysis we observed that ketone bodies can fuel the mitochondrial electron transfer chain (Fig. EV1). The presence of ketone bodies in the culture medium led to increased maximal mitochondrial respiration and ATP production (Fig. EV1D and F). In summary, the data suggest that ketone bodies can be an alternative source for the generation of biomass (Fig. 2) and ATP in ECs.

Figure 3A-D: From the data shown, it is not completely clear whether cell growth, proliferation, or both are affected. Simply counting the cells would provide insight into the response to ketone bodies.

The xCelligence technology allows such discrimination. Nevertheless, we added data by counting the cells on the plate and this also showed that both ketone bodies increase cell absolute EC numbers after 24, 47 and 72 hours of culture (Fig. EV2A-F).

Figure 3G-J: These figure panels show a lot of processed data, making it impossible to assess the data. Representative images of the angiogenic assays should be delivered.
We have added representative images of the angiogenesis assay (Fig. 3G and H).

The gene expression changes in cardiac endothelial cells in response to the ketogenic diet are minute. In light of these subtle changes, it is essential to validate some of the described targets (e.g., PDK4).
We have tested gene expression in isolated cardiac ECs from an independent experiment and validated the regulation of Pdk4 and Hmgcs2 (Fig. EV3D).

Figure 5E: Which cell cycle genes are regulated by the ketogenic diet and in which direction. The enrichment analysis per se does not provide insight into whether the proliferation is increased. Genes that cause a cell cycle arrest could also drive the relative enrichment in this category.
We now show these genes as a heat map so that both the genes per se and their regulation can be seen (Fig. EV3E).

The GSEA analyses in Figure 5F, G looks striking. However, given that overall gene expression are modest, one wonders how these gene set analysis results were obtained. Was the entire transcriptome taken into consideration or only a subset of genes? This should be explained in the Methods part of the paper.
This information was added to the Methods part.

Figure 6A, B.: The tissue sections should be labeled for a pan-endothelial marker to illustrate the tissue architecture. Along these lines, is the microvascular density altered in mice on a ketogenic diet? In which part of the cardiac muscle was the analysis performed? Left or right ventricle?
CD31 staining (a pan endothelial marker) of the left ventricle is shown in Fig. EV4C.

Figure 7: Again, original data is scarce in this figure. Representative images of the heart and its vasculature need to be shown for different treatment groups.
We have added these images (Fig. EV5C and D).

Referee #3 (Remarks for Author):

The present interesting manuscript is well written and structured. In this manuscript the authors present novel data regarding the metabolism of cardiac endothelial cells. Specifically, the authors found that cardiac endothelial cells are capable of oxidizing ketone bodies and that ketone bodies might promote cardiac endothelial cell proliferation, migration and angiogenic sprouting. Moreover, ketogenic diet is transiently increasing cardiac endothelial cell proliferation in vivo. In addition, in a model of pressure

overload-induced cardiac hypertrophy ketogenic diet maintained vessel density. The data are interesting and novel and provide insight on the metabolism of cardiac endothelial cells. However, the data are incomplete in the present form and there are some additional issues, which have to be experimentally addressed by the authors.

We thank the reviewer for her/his very positive judgement of our manuscript.

Major points:
# 1
a) I suggest to the authors to comparatively study the expression of SCOT and BDH1 in murine and human endothelial cells from different tissues.
This is an outstanding question which was also raised by the other two referees. To address this, we performed several experiments. First, we isolated primary ECs from murine adipose tissue, skeletal muscle and cardiac muscle. We also used primary human umbilical vein ECs (HUVEC), adipose tissue ECs and cardiac microvascular ECs. All of these ECs expressed SCOT and BDH1 (Fig. 1F). Secondly, we analyzed the single cell RNA sequencing atlas of endothelial cells from eleven mouse organs (published by Peter Carmeliet' group in *Cell* 2020;180(4):764-779.e20. PMID: 32059779). BDH1 was not detectable in this atlas, which might be due to the fact that droplet-based sequencing was employed. The sequencing depth is not as high as with SMARTseq protocols therefore transcripts with low expression strength are often not detected. There was however SCOT expression in ECs of all eleven organs. In the heart, SCOT expression was detected in all EC types such as arterial, venous, capillary (Appendix Fig. S1). Thirdly, we tested whether ketone bodies promote sprouting angiogenesis also in human umbilical vein endothelial cells (HUVEC) and indeed this was the case (Fig. EV2). In summary, the data suggest that ketone body oxidation in not unique to cardiac ECs. However, in vivo ketogenic diet only promoted EC proliferation in the heart (Fig. 6F). This is why we focused the paper on cardiac ECs.

b) How is the expression of SCOT and BDH1 regulated in endothelial cells ?
- Is starvation in vitro and ketogenic diet in vivo affecting the expression of SCOT and BDH1 expression in endothelial cells from heart and other tissues ?
- How do angiogenic growth factors, inflammatory cytokines and hypoxia affect the expression of SCOT and BDH1 in cardiac and non-cardiac endothelial cells ?
Ketogenic diet led to no changes in BDH1 expression. However, expression of Oxct1 (SCOT) was significantly lower at 3 and 7 days after starting the diet, but no longer at 14 days of ketogenic diet (Fig. EV3C).
To test for potential upstream regulators of Oxct1 and Bdh1, immortalized murine cardiac ECs (MCEC) as well as primary human cardiac microvascular ECs and HUVECs were treated with inflammatory cytokines (IL1b, IL6, TNFa), pro-angiogenic VEGF-A, cultured under normoxic vs. hypoxic conditions and starved vs. FCS-treated conditions. However, these experiments did not lead to the identification of an upstream regulatory factor (Appendix Fig. S2).

#2
Which is the effect of ketone bodies on murine and human non-cardiac endothelial cells regarding proliferation, migration and angiogenic sprouting ?
The data on murine cardiac ECs is shown in Fig. 3. In addition, we performed angiogenesis assays and BrdU incorporation assays with human umbilical vein ECs (HUVECs) (Fig. EV2G-I).

This showed that ketone bodies stimulate angiogenesis also in these cells which were isolated from a very different organ of a different species. As such, the pro-angiogenic effect is not specific for the heart, at least under *in vitro* conditions.
The establishment of angiogenesis assays using other organ-specific ECs is difficult and very time consuming. As the single cell data analysis of 11 different organs showed SCOT expression in all ECs (Appendix Fig. S1) it is very likely that all of these are in principle capable to use these as a source for biomass and angiogenesis. Given, the in vivo effects are predominantly in the heart (Fig 6F), we focused the paper on cardiac ECs.

#3

Which is the molecular explanation for the proposed differential response of cardiac endothelial cells to ketogenic diet in comparison to non-cardiac endothelial cells in regard to proliferation ? Are cardiomyocyte-derived factors involved in this differential response of cardiac endothelial cells ?

This is an outstanding question and we do not have an answer. Indeed, we speculate that local factors, such as cardiomyocyte secreted factors control the proliferation of ECs. In principle, ECs in the adult organism are in a deep quiescent state. As mentioned in the response to reviewer #1, it is very difficult to stimulate angiogenesis in an adult mouse. Multiple trials failed to e.g. stimulate cardiac angiogenesis after heart infarction by the application of potent proangiogenic growth factors like VEGF. However, also in the tumor model which requires angiogenesis for rapid tumor growth we could not see an effect by ketogenic diet (Fig. EV4 H- J). However, in this context there are already numerous pro-angiogenic factors present that the addition of another one probably makes no difference. In summary, we think that the local microenvironment prevents vessel sprouting in organs of the adult mouse which can only be overcome by a strong stimulus such as wounding. However, when using such models, there is already such a high amount of pro-angiogenic factors present that the addition of a single metabolite has no further measurable effect. We speculate that ketone bodies might be helpful in preventing vessel regression, e.g. the data of the heart hypertrophy model (Fig. 7D) indicates this, however, more sophisticated studies will be needed to clarify this in the future.

#4

The authors demonstrated that ketogenic diet over a longer period (for six weeks) did not result in increased proliferation of cardiac endothelial cells. Therefore, I suggest to the authors to study the effects of a ketogenic diet over 6 weeks on the expression of SCOT and BDH1 in endothelial cells isolated from mice hearts.

At 6 weeks, we do not see significant changes in endothelial Bdh1 and SCOT expression levels due to ketogenic diet. Given that the Editor gave us three months for the revision of our paper and that we do not have a valid permit to keep animals on ketogenic diet beyond this time point we cannot perform this experiment. Given the time points we already provide in this paper we do not expect to gain important additional information when keeping mice longer on ketogenic diet. This is also based on the fact that feeding this diet over longer periods leads to weight gain and this itself might change gene expression patterns etc.

8th Dec 2021

Dear Andreas,

Thank you for the submission of your revised manuscript to EMBO Molecular Medicine. We have now received the enclosed reports from the three referees who re-reviewed your manuscript. As you will see, they are supportive of publication, and I am therefore pleased to inform you that we will be able to accept your manuscript, once the following minor points will be addressed:

1/ Main manuscript text
- Please remove the yellow highlights in the text, accept the changes, and only keep in track changes mode any new modification.
- Material and methods:
o Cells: please indicate whether the cells were tested for mycoplasma contamination.
o Animal studies: please indicate gender of the mice.
o Human samples: please include the full statement that informed consent was obtained from all subjects and that the experiments conformed to the principles set out in the WMA Declaration of Helsinki and the Department of Health and Human Services Belmont Report.
- Data Availability Section: Primary datasets produced in this study need to be deposited in an appropriate public database, and the accession numbers and database listed under 'Data Availability' (i.e. raw RNAseq data should be deposited in databases such as GEO). Please update the checklist accordingly.

2/ Figures:
- Please indicate in legends the exact p= values, not a range. You may provide a supplemental table with all exact p-values if you prefer.
- Please remove the Dataset EV legend from the main manuscript file and insert it into the Dataset sheet in a separate tab.
- Please remove the Appendix legends from the main manuscript file and insert it into the Appendix file with a Table of Content.
- Fig EV5B contains error bars based on n=2, but the panel shows variance and statistics. Could you please clarify?

3/ Thank you for providing Source Data. Please upload them as one file per figure, and kindly check the labeling of the blots.

4/ Checklist:
Section B/5: could you please clarify "mostly"
Section E: due to the use of human samples, please fill in parts 11/12
Section F18/19: please update (See above).

5/ Thank you for providing a synopsis text. I included minor modifications, please let me know if you agree with the following, or amend as you see fit:
Vascular endothelial cells are shown to be capable of taking up and oxidizing ketone bodies, which enhances cell proliferation, migration and vessel sprouting.
- Expression of SCOT, the key enzyme for ketone body oxidation, was detected in endothelial cells from different vascular beds.
- Endothelial cells can oxidize ketone bodies to generate acetyl-CoA, biomass and ATP.
- Ketone bodies stimulate proliferation and tube formation of cultured endothelial cells.
- Ketogenic diet transiently increases endothelial cell proliferation in the heart and prevents capillary rarefication in a model of cardiac hypertrophy.

Please resize your synopsis picture (550 px wide x 300-600 px high) and make sure the text remains legible.

6/ As part of the EMBO Publications transparent editorial process initiative (see our Editorial at http://embomolmed.embopress.org/content/2/9/329), EMBO Molecular Medicine will publish online a Review Process File (RPF) to accompany accepted manuscripts.
This file will be published in conjunction with your paper and will include the anonymous referee reports, your point-by-point response and all pertinent correspondence relating to the manuscript. Let us know whether you agree with the publication of the RPF.
Please note that the Authors checklist will be published at the end of the RPF.

I look forward to receiving your revised manuscript.

With my best wishes,

Lise

Lise Roth, PhD
Editor
EMBO Molecular Medicine

*** PLEASE NOTE *** As part of the EMBO Publications transparent editorial process initiative (see our Editorial at https://www.embopress.org/doi/pdf/10.1002/emmm.201000094), EMBO Molecular Medicine will publish online a Review Process File to accompany accepted manuscripts.

In the event of acceptance, this file will be published in conjunction with your paper and will include the anonymous referee reports, your point-by-point response and all pertinent correspondence relating to the manuscript. If you do NOT want this file to be published, please inform the editorial office at contact@embomolmed.org.

To submit your manuscript, please follow this link:

Link Not Available

*Additional important information regarding Figures

Each figure should be given in a separate file and should have the following resolution:
Graphs 800-1,200 DPI
Photos 400-800 DPI
Colour (only CMYK) 300-400 DPI"

Figures are not edited by the production team. All lettering should be the same size and style; figure panels should be indicated by capital letters (A, B, C etc). Gridlines are not allowed except for log plots. Figures should be numbered in the order of their appearance in the text with Arabic numerals. Each Figure must have a separate legend and a caption is needed for each panel.

*Additional important information regarding figures and illustrations can be found at https://bit.ly/EMBOPressFigurePreparationGuideline. See also figure legend preparation guidelines: https://www.embopress.org/page/journal/17574684/authorguide#figureformat

The system will prompt you to fill in your funding and payment information. This will allow Wiley to send you a quote for the article processing charge (APC) in case of acceptance. This quote takes into account any reduction or fee waivers that you may be eligible for. Authors do not need to pay any fees before their manuscript is accepted and transferred to our publisher.

***** Reviewer's comments *****

Referee #1 (Remarks for Author):

The authors have addressed all my comments. With the new data the manuscript shows very convincingly that ketone bodies oxidation is relevant for cardiac endothelial cell proliferation. There are no further comments.

Referee #2 (Comments on Novelty/Model System for Author):

The revised manuscript is slightly improved. However, I am somewhat disappointed by the rather superficial and minimalistic response to the reviewer comments. Overall, the work is publishable but not with highest priority.

Referee #2 (Remarks for Author):

Weis et al. provide a revised version of their manuscript, which is slightly improved and, in general, publishable.

Referee #3 (Remarks for Author):

No additional comments.

The authors performed the requested editorial changes.

24th Jan 2022

Dear Andreas,

Thank you for sending the revised files. I am pleased to inform you that your manuscript is accepted for publication and is now being sent to our publisher to be included in the next available issue of EMBO Molecular Medicine!

We would like to remind you that as part of the EMBO Publications transparent editorial process initiative, EMBO Molecular Medicine will publish a Review Process File online to accompany accepted manuscripts. If you do NOT want the file to be published or would like to exclude figures, please immediately inform the editorial office via e-mail.

Congratulations on your nice study!

With my best wishes,

Lise

Lise Roth, Ph.D
Scientific Editor
EMBO Molecular Medicine

Follow us on Twitter @EmboMolMed
Sign up for eTOCs at embopress.org/alertsfeeds

EMBO PRESS

YOU MUST COMPLETE ALL CELLS WITH A PINK BACKGROUND ➜

PLEASE NOTE THAT THIS CHECKLIST WILL BE PUBLISHED ALONGSIDE YOUR PAPER

Corresponding Author Name: Andreas Fischer

Journal Submitted to: EMBO Molecular Medicine
Manuscript Number: EMM-2021-14753

USEFUL LINKS FOR COMPLETING THIS FORM

http://www.antibodypedia.com
http://1degreebio.org
http://www.equator-network.org/reporting-guidelines/improving-bioscience-research-repor
http://grants.nih.gov/grants/olaw/olaw.htm
http://www.mrc.ac.uk/Ourresearch/Ethicsresearchguidance/Useofanimals/index.htm
http://ClinicalTrials.gov
http://www.consort-statement.org
http://www.consort-statement.org/checklists/view/32-consort/66-title
http://www.equator-network.org/reporting-guidelines/reporting-recommendations-for-tumo
http://datadryad.org
http://figshare.com
http://www.ncbi.nlm.nih.gov/gap
http://www.ebi.ac.uk/ega
http://biomodels.net/
http://biomodels.net/miriam/
http://jjj.biochem.sun.ac.za
https://osp.od.nih.gov/biosafety-biosecurity-and-emerging-biotechnology/
http://www.selectagents.gov/

Reporting Checklist For Life Sciences Articles (Rev. June 2017)

This checklist is used to ensure good reporting standards and to improve the reproducibility of published results. These guidelines are consistent with the Principles and Guidelines for Reporting Preclinical Research issued by the NIH in 2014. Please follow the journal's authorship guidelines in preparing your manuscript.

A- Figures
1. Data
The data shown in figures should satisfy the following conditions:

- the data were obtained and processed according to the field's best practice and are presented to reflect the results of the experiments in an accurate and unbiased manner.
- figure panels include only data points, measurements or observations that can be compared to each other in a scientifically meaningful way.
- graphs include clearly labeled error bars for independent experiments and sample sizes. Unless justified, error bars should not be shown for technical replicates.
- if n< 5, the individual data points from each experiment should be plotted and any statistical test employed should be justified
- Source Data should be included to report the data underlying graphs. Please follow the guidelines set out in the author ship guidelines on Data Presentation.

2. Captions

Each figure caption should contain the following information, for each panel where they are relevant:

- a specification of the experimental system investigated (eg cell line, species name).
- the assay(s) and method(s) used to carry out the reported observations and measurements
- an explicit mention of the biological and chemical entity(ies) that are being measured.
- an explicit mention of the biological and chemical entity(ies) that are altered/varied/perturbed in a controlled manner.

- the exact sample size (n) for each experimental group/condition, given as a number, not a range;
- a description of the sample collection allowing the reader to understand whether the samples represent technical or biological replicates (including how many animals, litters, cultures, etc.).
- a statement of how many times the experiment shown was independently replicated in the laboratory.
- definitions of statistical methods and measures:
  - common tests, such as t-test (please specify whether paired vs. unpaired), simple $\chi 2$ tests, Wilcoxon and Mann-Whitney tests, can be unambiguously identified by name only, but more complex techniques should be described in the methods section;
  - are tests one-sided or two-sided?
  - are there adjustments for multiple comparisons?
  - exact statistical test results, e.g., P values = x but not P values < x;
  - definition of 'center values' as median or average;
  - definition of error bars as s.d. or s.e.m.

Any descriptions too long for the figure legend should be included in the methods section and/or with the source data.

In the pink boxes below, please ensure that the answers to the following questions are reported in the manuscript itself. Every question should be answered. If the question is not relevant to your research, please write NA (non applicable). We encourage you to include a specific subsection in the methods section for statistics, reagents, animal models and human subjects.

B- Statistics and general methods

Please fill out these boxes ➜ (Do not worry if you cannot see all your text once you press return)

1.a. How was the sample size chosen to ensure adequate power to detect a pre-specified effect size?	We did not use any statistical test to pre-determine sample size. The sample size was chosen based on previous experience.
1.b. For animal studies, include a statement about sample size estimate even if no statistical methods were used.	We did not use any statistical test to pre-determine sample size. The sample size was chosen based on previous experience.
2. Describe inclusion/exclusion criteria if samples or animals were excluded from the analysis. Were the criteria pre-established?	The criteria for animal experiments were pre-established by the Ethics Committee. Mice showing signs of severe pain, weight loss more than 20%, insufficient eating and drinking, apathy had to be sacrificed and were excluded from the analysis.
3. Were any steps taken to minimize the effects of subjective bias when allocating animals/samples to treatment (e.g. randomization procedure)? If yes, please describe.	No
For animal studies, include a statement about randomization even if no randomization was used.	We did not use any randomization procedure.
4.a. Were any steps taken to minimize the effects of subjective bias during group allocation or/and when assessing results (e.g. blinding of the investigator)? If yes please describe.	No blinding was done.
4.b. For animal studies, include a statement about blinding even if no blinding was done	No blinding was done.
5. For every figure, are statistical tests justified as appropriate?	Yes
Do the data meet the assumptions of the tests (e.g., normal distribution)? Describe any methods used to assess it.	Yes, data sets were checked for normal distribution using Shapiro-Wilk test.
Is there an estimate of variation within each group of data?	Yes, variance in data sets is represented as standard deviation.

Is the variance similar between the groups that are being statistically compared?	Statistics were performed using GraphPad Prism software which helped to perform the appropriate statistical tests and correction for each individual experiment.

C- Reagents

6. To show that antibodies were profiled for use in the system under study (assay and species), provide a citation, catalog number and/or clone number, supplementary information or reference to an antibody validation profile. e.g., Antibodypedia (see link list at top right), 1DegreeBio (see link list at top right).	This information is included in Materials and Methods section under subheading Western Blot analysis.
7. Identify the source of cell lines and report if they were recently authenticated (e.g., by STR profiling) and tested for mycoplasma contamination.	HUVECs and adipose tissue ECs were freshly isolated from umbilical cords and adipose tissue biopsies in our lab. MCECs were routinely tested for mycoplasma contamination.

* for all hyperlinks, please see the table at the top right of the document

D- Animal Models

8. Report species, strain, gender, age of animals and genetic modification status where applicable. Please detail housing and husbandry conditions and the source of animals.	Animals were housed under specific pathogen-free barrier conditions at 21 ± 2 °C with 60 % humidity and a 12-hour light /dark rhythm. Wild type C57BL/6J mice were purchased from Janvier Labs.
9. For experiments involving live vertebrates, include a statement of compliance with ethical regulations and identify the committee(s) approving the experiments.	All animal procedures were performed in accordance with institutional and national regulations and approved by local committees for animal experimentation (RP Karlsruhe, DKFZ, Heidelberg University).
10. We recommend consulting the ARRIVE guidelines (see link list at top right) (PLoS Biol. 8(6), e1000412, 2010) to ensure that other relevant aspects of animal studies are adequately reported. See author guidelines, under 'Reporting Guidelines'. See also: NIH (see link list at top right) and MRC (see link list at top right) recommendations. Please confirm compliance.	We confirm compliance with the guidelines.

E- Human Subjects

11. Identify the committee(s) approving the study protocol.	Institutional Review Board of the Medical Faculty of the University of Heidelberg
12. Include a statement confirming that informed consent was obtained from all subjects and that the experiments conformed to the principles set out in the WMA Declaration of Helsinki and the Department of Health and Human Services Belmont Report.	Adipose tissue ECs and HUVECs were isolated from biopsies or umbilical cords and collected from patients at the University Hospital Heidelberg. Collection was approved by the Institutional Review Board of the Medical Faculty of the University of Heidelberg. Informed consent was obtained from all subjects and experiments conformed to the principles set out in the WMA Declaration of Helsinki and the Department of Health and Human Services Belmont Report.
13. For publication of patient photos, include a statement confirming that consent to publish was obtained.	NA
14. Report any restrictions on the availability (and/or on the use) of human data or samples.	There are no restrictions to report.
15. Report the clinical trial registration number (at ClinicalTrials.gov or equivalent), where applicable.	NA
16. For phase II and III randomized controlled trials, please refer to the CONSORT flow diagram (see link list at top right) and submit the CONSORT checklist (see link list at top right) with your submission. See author guidelines, under 'Reporting Guidelines'. Please confirm you have submitted this list.	NA
17. For tumor marker prognostic studies, we recommend that you follow the REMARK reporting guidelines (see link list at top right). See author guidelines, under 'Reporting Guidelines'. Please confirm you have followed these guidelines.	NA

F- Data Accessibility

18: Provide a "Data Availability" section at the end of the Materials & Methods, listing the accession codes for data generated in this study and deposited in a public database (e.g. RNA-Seq data: Gene Expression Omnibus GSE39462, Proteomics data: PRIDE PXD000208 etc.) Please refer to our author guidelines for 'Data Deposition'.<br><br>Data deposition in a public repository is mandatory for:<br>a. Protein, DNA and RNA sequences<br>b. Macromolecular structures<br>c. Crystallographic data for small molecules<br>d. Functional genomics data<br>e. Proteomics and molecular interactions	We have attached a supplementary spreadsheet containing differentially expressed genes analysed from RNAseq data along with p-values. Raw data: Gene Expression Omnibus GSE191173
19. Deposition is strongly recommended for any datasets that are central and integral to the study; please consider the journal's data policy. If no structured public repository exists for a given data type, we encourage the provision of datasets in the manuscript as a Supplementary Document (see author guidelines under 'Expanded View' or in unstructured repositories such as Dryad (see link list at top right) or Figshare (see link list at top right).	We have provided our dataset as a supplementary document.
20. Access to human clinical and genomic datasets should be provided with as few restrictions as possible while respecting ethical obligations to the patients and relevant medical and legal issues. If practically possible and compatible with the individual consent agreement used in the study, such data should be deposited in one of the major public access-controlled repositories such as dbGAP (see link list at top right) or EGA (see link list at top right).	NA
21. Computational models that are central and integral to a study should be shared without restrictions and provided in a machine-readable form. The relevant accession numbers or links should be provided. When possible, standardized format (SBML, CellML) should be used instead of scripts (e.g. MATLAB). Authors are strongly encouraged to follow the MIRIAM guidelines (see link list at top right) and deposit their model in a public database such as Biomodels (see link list at top right) or JWS Online (see link list at top right). If computer source code is provided with the paper, it should be deposited in a public repository or included in supplementary information.	NA

G- Dual use research of concern

22. Could your study fall under dual use research restrictions? Please check biosecurity documents (see link list at top right) and list of select agents and toxins (APHIS/CDC) (see link list at top right). According to our biosecurity guidelines, provide a statement only if it could.	NA
